# In Vitro and In Silico Evaluation of New 1,3,4-Oxadiazole Derivatives of Pyrrolo[3,4-*d*]pyridazinone as Promising Cyclooxygenase Inhibitors

**DOI:** 10.3390/ijms22179130

**Published:** 2021-08-24

**Authors:** Krzysztof Peregrym, Łukasz Szczukowski, Benita Wiatrak, Katarzyna Potyrak, Żaneta Czyżnikowska, Piotr Świątek

**Affiliations:** 1 Department of Medicinal Chemistry, Faculty of Pharmacy, Wroclaw Medical University, Borowska 211, 50-556 Wroclaw, Poland; krzysztof.peregrym@student.umed.wroc.pl; 2 Department of Pharmacology, Faculty of Medicine, Wroclaw Medical University, Mikulicza-Radeckiego 2, 50-345 Wroclaw, Poland; benita.wiatrak@umed.wroc.pl (B.W.); katarzyna.potyrak@student.umed.wroc.pl (K.P.); 3 Department of Inorganic Chemistry, Wroclaw Medical University, Borowska 211, 50-556 Wroclaw, Poland; zaneta.czyznikowska@umed.wroc.pl

**Keywords:** anti-inflammatory activity, 1,3,4-oxadiazole, molecular docking, double pharmacophore approach, pyridazinone, antioxidants, Mannich bases, cyclooxygenase

## Abstract

Since long-term use of classic NSAIDs can cause severe side effects related mainly to the gastroduodenal tract, discovery of novel cyclooxygenase inhibitors with a safe gastric profile still remains a crucial challenge. Based on the most recent literature data and previous own studies, we decided to modify the structure of already reported 1,3,4-oxadiazole based derivatives of pyrrolo[3,4-*d*]pyridazinone in order to obtain effective COX inhibitors. Herein we present the synthesis, biological evaluation and molecular docking studies of 12 novel compounds with disubstituted arylpiperazine pharmacophore linked in a different way with 1,3,4-oxadiazole ring. None of the obtained molecules show cytotoxicity on NHDF and THP-1 cell lines and, therefore, all were qualified for further investigation. In vitro cyclooxygenase inhibition assay revealed almost equal activity of new derivatives towards both COX-1 and COX-2 isoenzymes. Moreover, all compounds inhibit COX-2 isoform better than Meloxicam which was used as reference. Anti-inflammatory activity was confirmed in biological assays according to which title molecules are able to reduce induced inflammation within cells. Molecular docking studies were performed to describe the binding mode of new structures to cyclooxygenase. Investigated derivatives take place in the active site of COX, very similar to Meloxicam. For some compounds, promising druglikeness was calculated using in silico predictions.

## 1. Introduction

Compounds containing five-membered rings with one or more heteroatoms are extensively investigated in terms of their various biological activity. This characteristic moiety can be distinguished in the structure of numerous drugs, for example, in popular anti-inflammatory and analgesic agents such as Oxaprozin (oxazole), Fentiazac, Meloxicam (thiazole) or Celecoxib (pyrazole).

**Figure 1:**
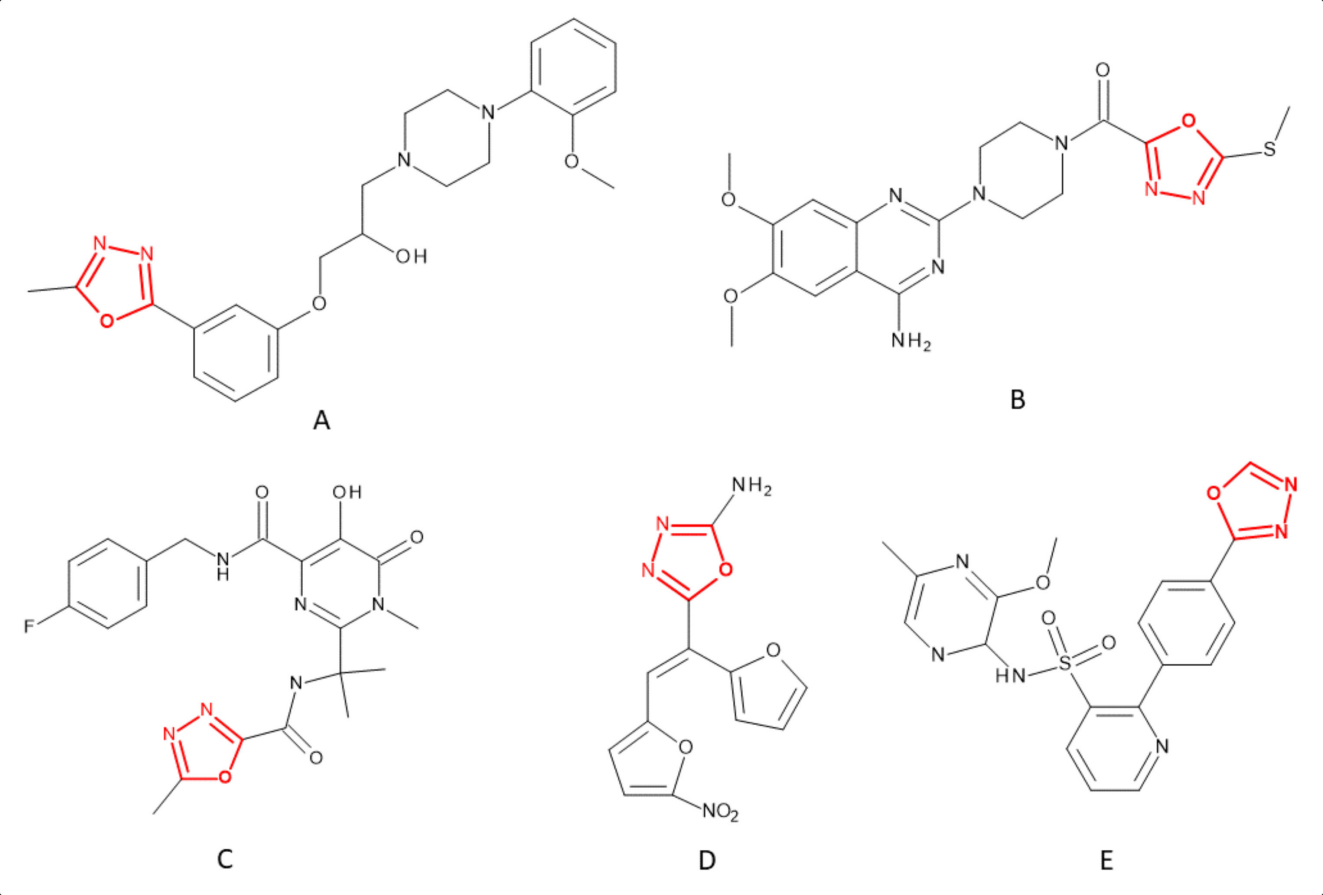
Chemical structures of some approved 1,3,4-oxadiazole ring-based drugs: Furamizole (**A**), Nesapidil (**B**), Raltegravir (**C**), Tiodazosin (**D**) and Zibotentan *(***E**).

When considering such five-membered pharmacophores, the 1,3,4-oxadiazole ring is probably one of the most important moieties in contemporary medicinal chemistry and drug design. According to the literature, compounds containing this structure have been reported to exert diverse and promising biological activities, such as antibacterial [1] and antimycobacterial [2], antifungal [3], antidiabetic [4], anticonvulsant [5], anticancer, anti-inflammatory and analgesic [6,7,8,9]. Needless to say, in the structure of currently available medicines, along with some still being developed and investigated, the 1,3,4-oxadiazole ring can be noticed in, e.g., Furamizole [10], Nesapidil [11], Raltegravir [12], Tiodazosin [13] and Zibotentan [14] (Figure 1).

In drug development, the 1,3,4-oxadiazole ring serves as a pharmacophore which enhances biological activity. Moreover, this moiety can play the role of a useful bioisostere of free carboxylic group. Such alternation has already been performed on many compounds in order to modify their pharmacological properties. Beta-site amyloid precursor protein cleaving enzyme 1 (BACE1) inhibitors, promising drugs in treatment of Alzheimer’s disease (not introduced yet), were modified in this way in order to improve blood–brain permeability due to increased lipophilicity [15]. A similar effect of enhanced cell-membrane penetrability was observed in the case of angiotensin II receptor type 1 (AT1) blockers (ARBs). It has been reported that replacement of the tetrazole ring with 1,3,4-oxadiazole in Candesartan improved oral bioavailability and in vivo activity [16]. Additionally, it has to be highlighted that the introduction of 1,3,4-oxadiaozole moiety might significantly diminish severe adverse effects which are related to the presence of free carboxylic group. This effect is especially well marked in reference to non-steroidal and anti-inflammatory drugs (NSAIDs) and their derivatives. NSAIDs are a wide group of medicines used in treatment of different inflammatory diseases and pain. They act as inhibitors of cyclooxygenase (COX), a membrane-bound enzyme which exists in three isoforms (COX-1, COX-2 and COX-3) responsible for conversion of arachidonic acid to prostaglandins (PGs) and thromboxane (TX) [17,18,19,20,21]. Therefore, COX plays a crucial role in the initiation and control of inflammation and pain. Unfortunately, NSAIDs are known for their various side effects related to COX inhibition, because PGs also have many physiological functions and play an important role in maintenance of homeostasis, especially in the gastrointestinal and cardiovascular system [17,18,19,20,21,22,23,24,25,26,27]. COX-1 is responsible for synthesis of prostaglandin I_2_ (PGI_2_), named as prostacyclin, which is essential in the production of cytoprotective mucus and bicarbonate. Additionally, free carboxylic group, present in most of those drugs, causes direct irritation in contact with mucosa cells due to the effect of ionic trap. NSAIDs do not become ionized when surrounded by an acidic stomach environment, but they dissociate easily after entering epithelial cells. Hence, for years many, new structures were being put under investigation in order to achieve and introduce new drugs devoid of gastrotoxicity, which strongly limits long-term treatment with NSAIDs. Initially, based on the theory that desired physiological effects are related to COX-1 isoform activity, while inflammation is under control of COX-2 dependent mediators, COX-2 selective inhibitors—COXIBs—have been introduced. Unfortunately, despite good anti-inflammatory activity, COXIBs were proven to increase the risk of cardiovascular events, which resulted in the worldwide market withdrawal of Rofecoxib [22,23,24,25,26,27,28]. Yet there is still a huge need for discovery of new potent cyclooxygenase inhibitors [22,23,24]. Replacement of free acidic group, characteristic for NSAIDs, with 1,3,4-oxadiazole is one of the most popular and promising synthetic approaches in medicinal chemistry nowadays [15,16]. Such modification performed on, e.g., Diclofenac [29] or Ibuprofen [30], allows to obtain potent anti-inflammatory agents with significantly decreased gastrotoxicity and increased COX-2 affinity.

In our previous paper, we have reported the synthesis of 1,3,4-oxadiazole based derivatives of pyrrolo[3,4-*d*]pyridazinone [31,32]. Introduction of 1,3,4-oxadiazole moiety to the pyrrolo[3,4-*d*]pyridazinone core was aimed to improve COX-2 affinity and reduce gastrotoxicity of investigated structures. Additionally, arylpiperazine pharmacophore was introduced into title structures via two different ways. As a result, we have received two series of compounds. The first were new Mannich base type derivatives, while the second were molecules with oxoethyl linker inspired by Dogruer’s theory, which claims that carbonyl group connected to the alkyl chain in the structure might strengthen its anti-inflammatory and analgesic activity [33,34].

When considering previously reported molecules, different unsubstituted or monosubstituted arylpiperazine/arylpiperidine residues could be distinguished in their structure. Investigated derivatives revealed promising cyclooxygenase inhibitory activity—some of them acted as specific COX-2 inhibitors, the other ones were selective towards this enzyme [31,32]. Taking into account that the binding pocket of isoenzyme COX-2 is bigger than that of COX-1 [35], we decided to introduce spacious, double substituted arylpiperazine pharmacophore in order to improve the COX-2/COX-1 inhibitory ratio of titled compounds. Therefore, in the current study, the derivatives bearing dimethyl or dihalo substituents in different positions of phenyl ring were designed, synthesized and then investigated. By such modification, we wanted to determine the impact of the double substituted arylpiperazine pharmacophore on compound activity and selectivity towards cyclooxygenase isoenzymes. Herein we wish to report the synthesis and comprehensive in vitro and in silico investigations of two series of novel pyrrolo[3,4-*d*]pyridazinone derivatives.

## 2. Results and Discussion

### 2.1. Chemistry

**Scheme 1:**
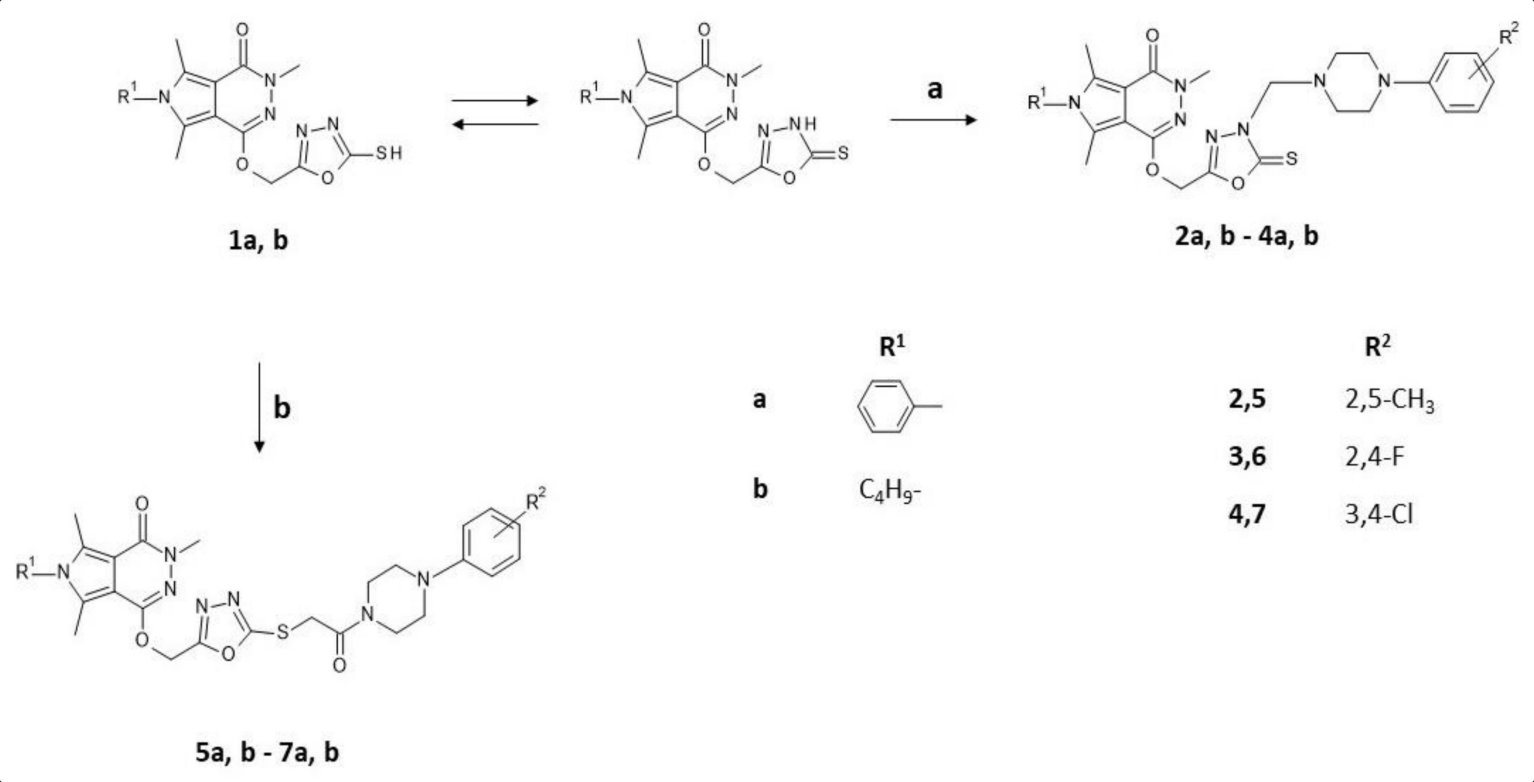
Synthesis and structures of final compounds **2a, b–7a, b**. Reagents and conditions: (**a**) C_2_H_5_OH, 37% HCHO, arylpiperazine, RT, stirring 5 h; (**b**) C_2_H_5_OH, C_2_H_5_ONa, 1-(2-chloro-1-oxoethyl) 4-arylpiperazine, reflux 5 h.

As a continuation of our previous investigations, the aim of this study was the synthesis and biological evaluation of new 1,3,4-oxadiazole based pyrrolo[3,4-*d*]pyridazinone derivatives **2a**, **b**–**7a**, **b**. Presented compounds were inspired not only by the structure–activity relationship of different anti-inflammatory compounds reported in the literature, but also by our results regarding the simultaneous introduction of double substituted arylpiperazine residue as a second pharmacophore, alongside with the 1,3,4-oxadiazole moiety incorporated into biheterocyclic scaffold of pyrrolo[3,4-*d*]pyridazinone. Two series of compounds were synthesized as shown in Scheme 1 (structures of new compounds are shown in Table S1 in Supplementary).

The compounds **1a, b** were synthesized according to our previously reported procedure [32]. In the first step, we conducted the formation of Mannich base type derivatives **2a, b**–**4a, b** through one-step reaction of **1a, b** with appropriate disubstituted 4-phenylpiperazine and 37% solution of formaldehyde in anhydrous ethanol, as it has already been described [32]. Subsequently, in order to achieve structures **5a, b**–**7a, b,** the reaction of **1a, b** was carried out in ethanol, alongside with the presence of sodium ethoxide and suitable 1-(2-chloro-1-oxoethyl)-4-disubstituted phenylpiperazine, as described in our earlier paper [31]. The formed precipitates were filtered off, washed with ethanol and purified by crystallization from this solvent. Progress of the syntheses was monitored by thin layer chromatography technique (TLC). Structures of final compounds were confirmed by spectroscopic studies—^13^C NMR, ^1^H NMR, MS and FT-IR. Mannich base type compounds **2a, b**–**4a, b** formation was verified by the presence of a peak registered around δ 5.07–5.09 ppm in ^1^H NMR spectra and near δ 70.11–70.56 ppm in the ^13^C NMR, which indicates occurrence of the methylene linker. Similarly, structures **5a, b**–**7a, b** were also verified through ^13^C NMR spectra, as peaks signal near δ 164.60–164.71 ppm for carbonyl (C=O) were registered, while typical signal for C=S bond (δ 177.89–178.90 ppm) is missing. Lack of this peak indicates that the compounds were formed by *S*-alkylation. Moreover, another two-proton singlet in the range of δ 4.38–4.42 ppm in ^1^H NMR spectra was registered. It can be associated with methylene group within 2-oxoethyl linker. Additionally, in the ^1^H NMR spectra of final compounds, signals of piperazine protons were recorded in range of δ 2.92–3.17 ppm for **2a, b**–**4a, b** and δ 2.89–3.84 for **5a, b**–**7a, b**.

### 2.2. Biological Evaluation

#### 2.2.1. Cytotoxicity Estimation

**Figure 2:**
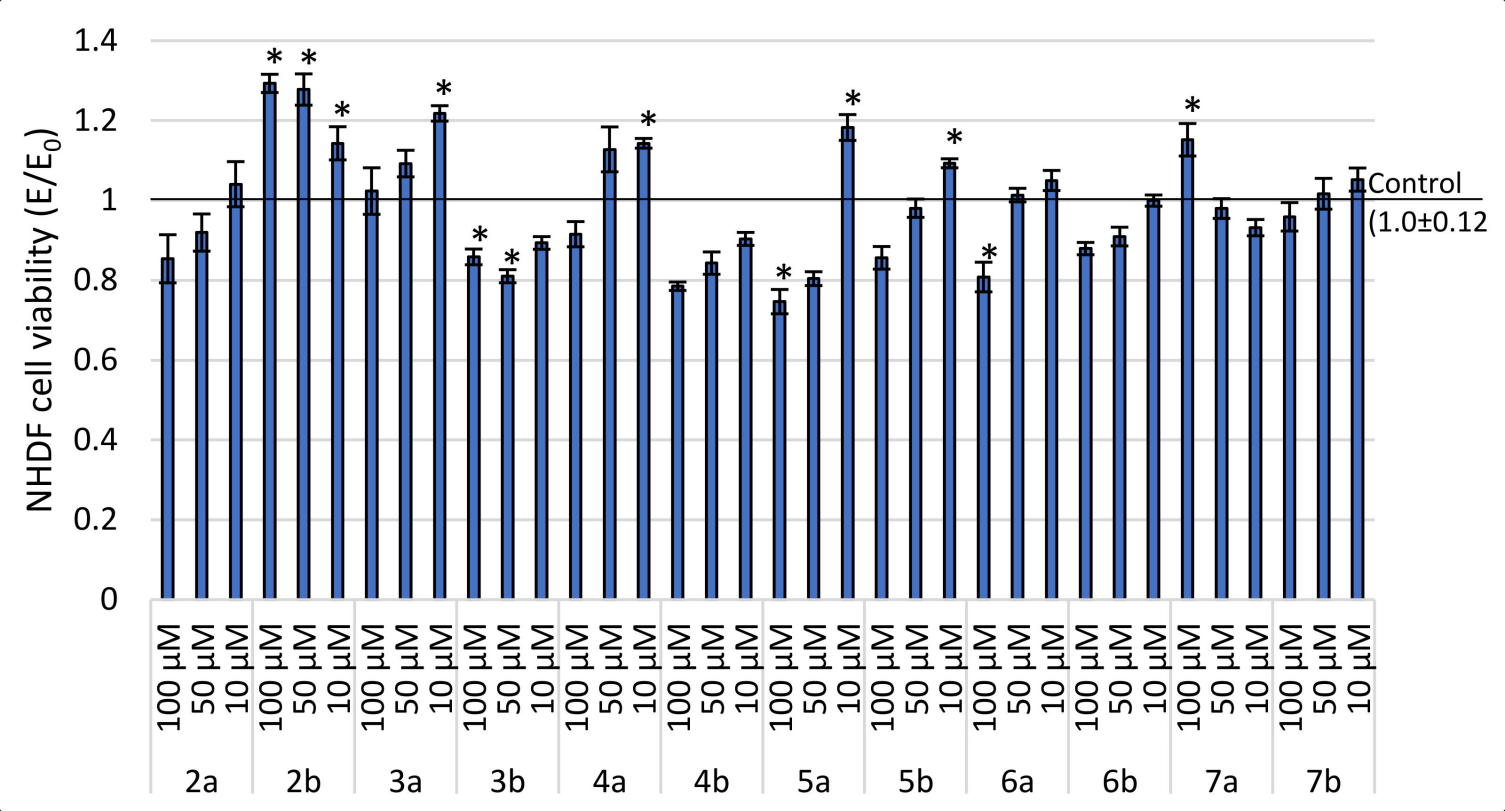
Impact of investigated compounds on normal human dermal fibroblasts measured in MTT assay; * p < 0.05—significant difference compared to control without tested compounds.

To select appropriate compounds for further evaluation, MTT assay was performed in order to estimate cytotoxicity of investigated derivatives. The assay was carried out using normal human dermal fibroblasts (NHDF) cell line. No decrease in viability of 30% or more was detected, thus no compound was classified to possess cytotoxic potential, although derivative **5a** caused a statistically significant highest decrease of 26%; however, when considering the lowest concentration, in this case the viability was improved much over negative control with statistical significance (Figure 2). Such improvement of cell viability over negative control level was also observed with statistical significance after exposure to compound **2b** in all concentrations, to compounds **3a, 4a, 5b** in the lowest and compound **7a** in the highest concentration, suggesting possible proliferative activity. Nevertheless, all compounds were qualified for further biological evaluation.

#### 2.2.2. In Vitro COX Inhibition Assay

In order to measure the ability of investigated derivatives to inhibit COX-1 and COX-2 activity, we performed in vitro COX inhibition study using Cayman’s COX Colorimetric Inhibitor Screening Assay Kit (cat.no. 701050). Every sample used was prepared in concentration of 100 μM in triplicate. Firstly, incubation was carried out (2 min, RT) and then Varioskan LUX microplate reader (Thermo Fisher Scientific, Waltham, MA, USA), at 590 nm wavelength, was used to estimate peroxidase activity. The results were obtained as IC_50_ values. The reference compounds used were Meloxicam, Celecoxib and Diclofenac. Results for COX-1 and COX-2 isoenzymes affinity are presented in Table 1.

All of 12 investigated final products **2**–**7** showed very similar inhibitory activity and almost equal affinity to both isoenzymes, in contrast to their precursors **1a** and **1b**, which inhibited COX-2 stronger than COX-1. It should be noted that the IC_50_ values determined for the precursors **1a**, **b** and products **2**–**7** against COX-2 were similar.

The COX-2/COX-1 selectivity ratio lower than 1.0 was observed in the case of nine examined molecules, where for compound **5a** it was the lowest, thus making it the most selective among all 12 compounds. Although the investigated derivatives did not show significant selectivity towards COX-2 isoenzyme, all have similar affinity to inducible form of cyclooxygenase as Meloxicam and present similar selectivity ratio to this reference drug. At the same time, activity towards COX-1 was comparable to Celecoxib. Although all values obtained are very close to equal, compound **2b** appears to be the most active in terms of COX-1 and **4a** in terms of COX-2.

Compared to our previous studies, the influence of introduced disubstituted phenyl ring could possibly diminish the selectivity towards COX-2 isoenzyme. With such modification, the nature of linker between arylpiperazine pharmacophore and 1,3,4-oxadiazole moiety seems to play a secondary role in the case of the binding mode of title compounds to the active site of COX. Still, the impact of oxadiazole presence is visible due to promising COX inhibitory activity of new derivatives.

#### 2.2.3. Cyclooxygenase Molecular Docking Study

Literature data revealed that both COX-1 and COX-2 isoenzymes have a similar molecular weight and ligand binding site. Their inhibitors show a slightly different mode of binding due to the amino acids sequence homology close to 65%. The replacement of the side chain of Ile523 for smaller Val523 and the changes of Tyr355 conformation influence the formation of additional binding pocket in COX-2, which includes Leu352, Ser353, Tyr355, Phe518 and Val523 amino acid residues. These differences determine the selectivity of the ligands and can be an important factor in design process [36].

In the present paper, the binding mode of the newly synthesized pyrrolo[3,4-*d*]pyridazinone derivatives to the active center of both COX-1 and COX-2 was predicted by using molecular docking protocol and compared with the position of Meloxicam and Diclofenac. The docking procedure was validated by the redocking of Meloxicam to the crystal structure of both enzymes. The results obtained the docking accuracy with the RMSD less than 1.5. Being aware that scoring functions used in the docking algorithms give only approximate values of free energy of binding, results were validated with biological activity measurements. Obtained data can be found in Table 2a,b. The binding affinity of ligands is related directly to the Gibbs energy of binding and is expressed by the following formula:

Δ*G_bind_* = [Δ*G _intermolecular_* + Δ*G _internal_* + Δ*G _tors_*] − Δ*G _unbound_*

Intermolecular interaction energy (Δ*E_int_*) is the sum of van der Waals, hydrogen bonding, desolvation and electrostatic terms between the inhibitor and the binding site of protein.

Δ*E_int_* = [Δ*E_vdw_* + Δ*E_hbond_* + Δ*E_desolv_*] + Δ*E_el_*

According to data received from in vitro and molecular docking study, all compounds can bind to the binding center of both COX-1 and COX-2 isoenzymes. The differences in potency of binding estimated during the docking study was not crucial. The free energy of binding (ΔG binding) for complexes with COX-1 ranges from −7.1 kcal/mol to −11.2 kcal/mol and for complexes with COX-2 from −10.3 kcal/mol to −12.9 kcal/mol. As can be seen, van der Waals forces and hydrogen bonding are the main origin of stabilization of protein–ligand complexes.

A detailed analysis of the binding mode of ligands to the active site of both enzymes is presented in Tables S6 and S7 in Supplementary and below.

**Figure 3:**
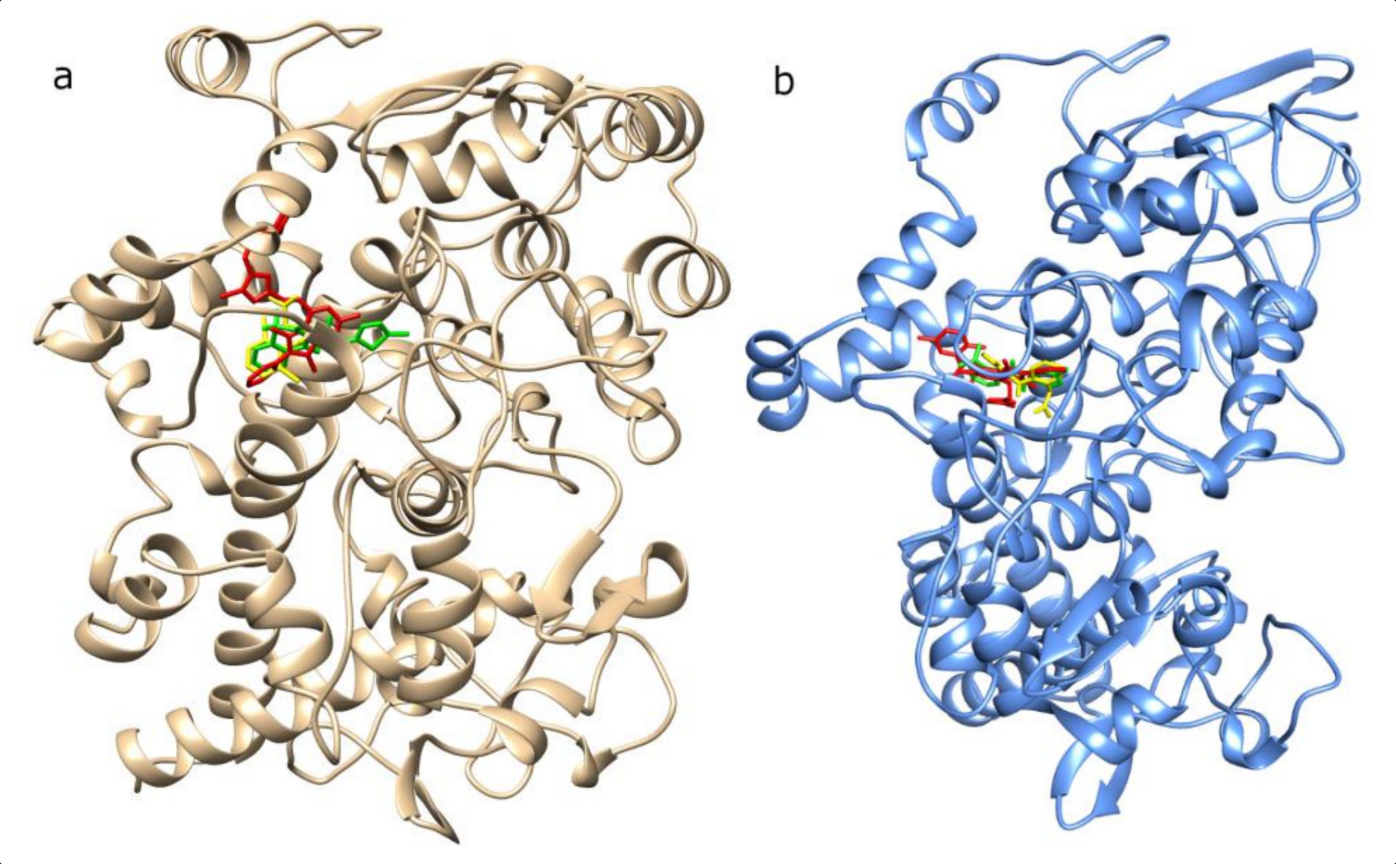
The binding mode of **2a** (red), Meloxicam (green) and Diclofenac (yellow) in the active center of (**a**) COX-1 and (**b**) COX-2.

When considering the influence of structural differences of compounds on their binding manner to the active site of the enzyme, the potency of binding of all 12 compounds was similar. As is presented in Table S5 in Supplementary Materials, most of the considered inhibitors can interact similarly to the classic NSAIDs, such as Diclofenac, forming hydrogen bonds with Arg120, Tyr355 and Ser350 (**2a** –**b, 3a, 4a, 5a, 6a**–**b**, **7b**) [37,38,39,40,41]. Moreover, except for **4a**, all compounds, similar to Meloxicam, penetrate the hydrophobic pocket of COX-2 formed by Ser353, Leu384, Tyr385, Trp387, Val523 and Met522 [42]. The binding mode of **2a** in comparison to Meloxicam and Diclofenac in the active center of both isoenzymes is presented on Figure 3 below and in Table S5 in Supplementary.

Compound **2a** binding to the COX-1 can form two hydrogen bonds with Tyr355, similar to Diclofenac. The pyrrolo[3,4-*d*]pyridazinone part of the molecule is localized in the hydrophobic pocket and interacts via van der Waals interactions with Leu117, Leu352, Met522, Ile523, Gly526, Ser530, Leu531, Leu534 and Leu535. Its phenyl substituent can interact with Ile345 and Val349 via π-alkyl and π-σ interactions, respectively. The 1,3,4-oxadiazole moiety binds mainly through alkyl and π-alkyl interactions with Met113, Val116, Leu357 and Leu359. Phenylpiperazine group form π-cation interactions with Arg83 and Arg120 (see Supplementary Figure S1a in Table S5). When considering COX-2 binding pocket, phenylpiperazine moiety of **2a** binds as Meloxicam to the cavity including Arg120, Tyr355, Val523, Gly526, Ala527, Ser530 and Leu531, which probably arises due to the conformation of Tyr355. Both pyrrolo[3,4-*d*]pyridazinone and 1,3,4-oxadiazole moieties interact mainly by alkyl and π-alkyl interactions with Leu93, Met113, Val116, Leu117, Phe357, Leu359, Leu531, Leu534 and Met535. Data are presented in Figure S1a, Table S5 in Supplementary.

As determined, **2b** forms van der Waals interactions with Ser353, Tyr385, Gly526, Ala527, Ser530 and Leu531 with COX-1. In this case, two hydrogen bonds are created with Arg120 and Tyr355 amino acid residues. Π-cation interactions of pyrrolo[3,4-*d*]pyridazinone and 1,3,4-oxadiazole rings with Arg120 are also present. As can be seen, **2b** takes orientation in the binding site of COX-2 (Supplementary Figure S2b in Table S5), similar to **2a** and **3b**. Van der Waals and alkyl interactions play dominant role in COX-2-inhibitor complex stabilization.

As presented in Figure S3a, Table S5 in Supplementary, **3a** can form three hydrogen bonds with Arg120 and Tyr355 as classic anti-inflammatory agents and one hydrogen bond with His513. In this case, the phenyl substituent of pyrrolo[3,4-*d*]pyridazinone moiety is surrounded by Leu352, Trp387, Phe518, Met522, Gly526 and Ser 530. The addition of fluorine atoms to phenylpiperazine scaffold is responsible for halogen interactions with Pro86 and Glu524. Similar to Meloxicam, phenylpiperazine group of **3a** in the binding center of COX-2 is exposed towards hydrophobic amino acids (Leu352, Trp387, Phe518, Met522, Gly526 and Ser 530), which are mainly involved in alkyl and van der Waals interactions. There is also a possibility of H-bonding interactions which involved pyrrolo[3,4-*d*]pyridazinone moiety with Arg120 and Tyr355 of COX-2 (Figure S3b, Table S5 in Supplementary).

The compound **3b** binds to the COX-1 differently. It forms only one hydrogen bond with Asn375. Additionally, one halogen interaction with Phe529 is present. The hydrophobic and polar amino acids Leu93, Val116, Leu352 and Tyr357 are localized in the vicinity of aliphatic chain. As we mentioned above, in the case of interactions of **3b** in the active center of COX-2, van der Waals and alkyl forces are crucial. Here, pyrrolo[3,4-*d*]pyridazinone group and its aliphatic substituent can penetrate a hydrophobic pocket formed by Val349, Leu384, Tyr385, Trp387, Met522 and Gly526, similar to Meloxicam.

As can be observed on Figure S5a in Table S5 in Supplementary, compound **4a** exhibits a unique binding configuration in the binding cavity of COX-1. The pyrrolo[3,4-*d*]pyridazinone moiety forms π-σ and π-sulfur interactions with Val116 and Met113, respectively, and additionally interacts via π-alkyl and van der Waals interactions with Leu93, Arg120, Ile345 Val349 and Ala527. One hydrogen bond is created between Ser530 and nitrogen atom of oxadiazole ring, which simultaneously interact with Leu352, Tyr385, Trp387. Similar configuration for **4a** is observed in the binding center of COX-2.

As determined, compound **4b** interacts with COX-1 via van der Waals interactions with Leu117, Arg120, Ser353, Met522, Gly526 and Ser530. Additionally, π-σ interactions with Val116, Leu352 and Tyr358 are formed. The pyrrolo[3,4-*d*]pyridazinone group of inhibitors occupied the characteristic pocket formed by Val349, Ieu352, Tyr385, Trp387, Phe518, Met522, Ala527 and Ser530 (Figure S6a in Table S5 in Supplementary). The best docking pose for **4b**-COX-2 is presented in Figure S6b in Table S5 in Supplementary. The main origin of stabilization in this case is also van der Waals and π-alkyl interactions. The aliphatic chain can deeply penetrate the binding cavity created by Leu352, Trp387, Met522 and Val523.

The binding pose of **5a** in the active center of COX-1 is presented in Figure S7a in Table S5 in Supplementary. The main origin of stabilization is π–type interactions. The pyrrolo[3,4-*d*]pyridazinone moiety interacts via π-σ and π-alkyl interactions with Val349, Ala527, Leu532 and Ile345, Leu352, Ile523, Ile534, respectively. Two conventional hydrogen bonds are created with Arg120 and Ser530. Similar to Diclofenac, **5a** can form three hydrogen bonds with Arg120, Tyr355 and Ser350 of COX-2. The pyrrolo[3,4-*d*]pyridazinone group occupies additional binding cavity of COX-2 and is located in close proximity of Leu352, Tyr385, TRP387, Phe518, Gly526, Ala527 and Val523.

Compound **5b** is almost located in the same binding place of COX-1 as **2a**. This way of binding is typical for the inhibitors such as Meloxicam. As can be seen in Figure S7a in Table S5 in Supplementary, pyrrolo[3,4-*d*]pyridazinone group occupied hydrophobic pocket formed by Val349, Ser353, Tyr385, Trp387, Ile523, Gly526 and Phe518. The π-alkyl and van der Waals interactions are also created between 1,3,4-oxadiazole moiety and Tyr355, Leu531, Val349 and Met113, Met522, Ala527, Ser530, respectively. The intermolecular interactions of compound **5b** in the active center of COX-2 are presented in Figure S8b in Table S5 in Supplementary. The 5b-COX-2 complex is stabilized mainly by van der Waals, π-alkyl and π-σ interactions. Similar to **2b** and **3b**, pyrrolo[3,4-*d*]pyridazinone moiety of **5b** binds to the binding pocket of COX-2 and is localized in the close vicinity of Leu352, Phe381, Tyr385, Trp387, Met522, Val523 and Gly526.

The intermolecular interactions of compound **6a** in the active center of COX-1 are presented in Figure S9a in Table S5 in Supplementary. The π-σ interactions are created between pyrrolo[3,4-*d*]pyridazinone rings and Val349 and Leu531. The oxadiazole-2-thione moiety interacts with Arg120 and Val116 via π-σ interactions and is also involved in van der Waals and alkyl interactions including Met113, Leu117, Leu359, Ile523 and Gly526. In this case, only one hydrogen bond is created. Similar to **2a** and **5a**, pyrrolo[3,4-*d*]pyridazinone group of 6a binds to the cavity created by Leu352, Phe381, Trp387, Phe518, Met522, Val523 and Gly526 of COX-2. In this case, the van der Waals forces and π-alkyl interactions play a dominant role. One hydrogen bond is created between the oxygen atom of oxadiazole ring and Ser530 amino acid residue (See Figure S9a in Table S5 in Supplementary).

The binding mode of **6b** in the active center of COX-1 exhibits some unfavorable interactions. The position of ligand is stabilized by π-type and van der Waals interactions. Three amide-π interactions are possible in the binding cavity of COX-2 between **6b** and Gly526 and Ser530 amino acid residues of protein. Similar to Meloxicam, pyrrolo[3,4-*d*]pyridazinone moiety is mainly exposed towards hydrophobic amino acids residues (Val349, Leu352, Tyr385, Trp387, Phe518, Met522, Val523, Gly526 and Ala527). Oxadiazole ring forms interactions with Arg120, Tyr348, Tyr355 and Ser530.

In the case of **7a** (Figure S11a, Table S5), its pyrrolo[3,4-*d*]pyridazinone moiety can also bind to the active site of COX-1 through H-bonding interactions with Ser530 and van der Waals interactions with Met522 and Gly526. Its phenyl substituent can form π-alkyl interactions with Ile345, Leu359 and Leu531. The amino acids residues of Ile523 interact via carbon hydrogen bond. Additionally, phenylpiperazine scaffold forms halogen and π-cation interactions with Pro86 and Arg120. The interactions with pyrrolo[3,4-*d*]pyridazinone and oxadiazole moieties are mainly responsible for activity of **7a** towards COX-2 (See Figure S11b, Table S5). The most important role is played by van der Waals and π-type interactions. Phenyl substituent is surrounded by Phe381, Leu384, Tyr385, Trp387, Met522 and Gly526. Oxadiazole ring interacts with Val532, Ala527, and Ser530.

In the case of binding to COX-1, **7b** makes some unfavorable interactions. Two hydrogen bonds with Arg120 and Ser530 are present. We can also observe two π-σ interactions with Met113 and Ile532. The van der Waals and π-alkyl interactions also play an important role. **7b** binds to the binding cavity of COX-2, similar to **6b** and **4b**. The pyrrolo[3,4-*d*]pyridazinone moiety is involved in interactions with Val349, Leu352, Tyr385, Trp387, Phe518, Met522, Val523, Gly526 and Ala527. The nitrogen of oxadiazole ring forms one hydrogen bond with Ser530 amino acid.

### 2.3. Evaluation of Anti-Inflammatory Activity within Cells

According to the cyclooxygenase inhibition studies described above, all compounds showed similar activity and affinity to both isoenzymes. Therefore, derivatives were further evaluated in terms of their anti-inflammatory potential within cells in three assays.

#### 2.3.1. Cells Regeneration—MTT Assay

**Figure 4:**
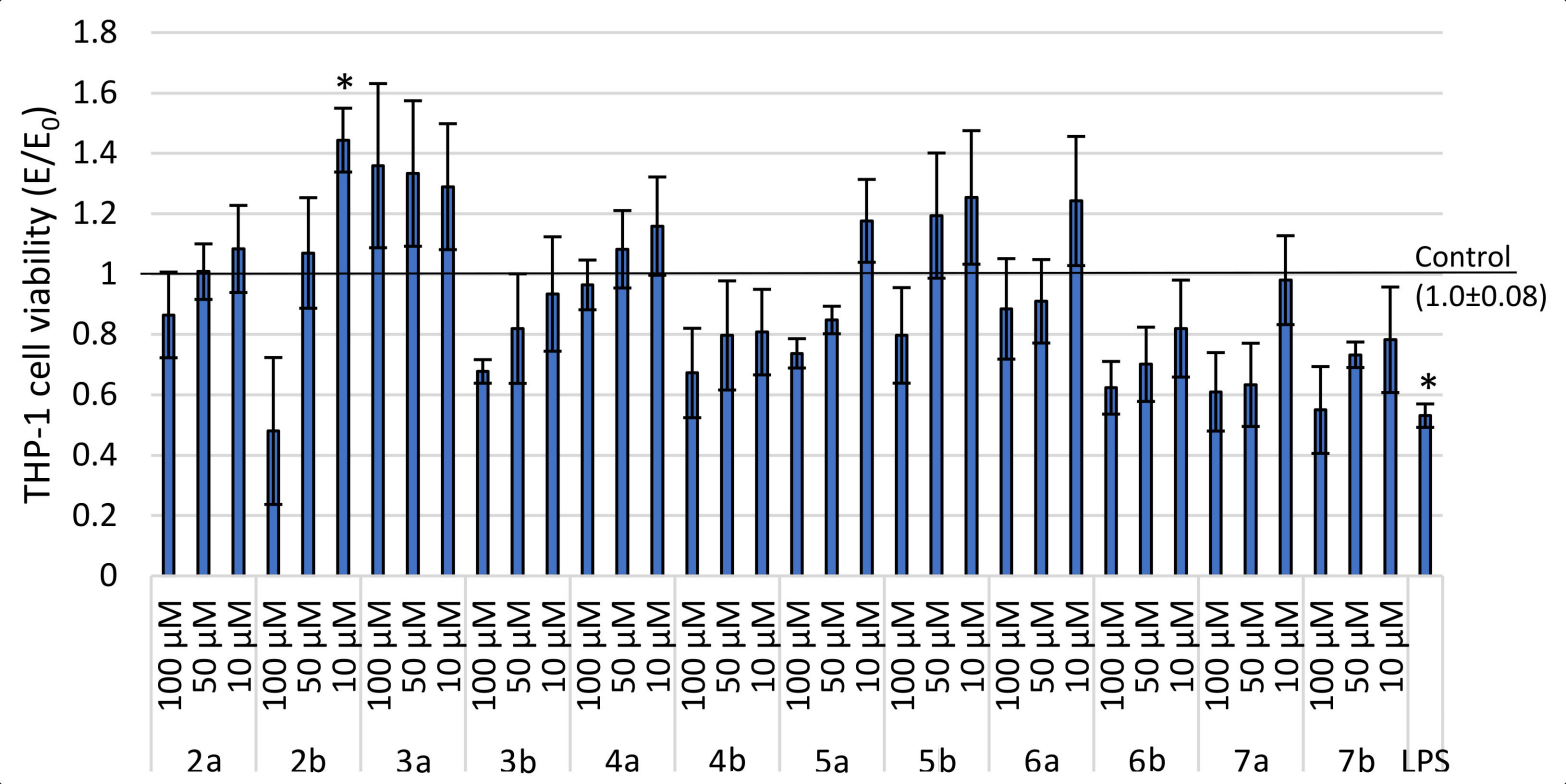
Impact of investigated compounds on THP-1 cells measured through MTT assay; * *p* < 0.05—significant difference compared to control with 50 μg/mL LPS and without compounds.

THP-1 cell line, firstly damaged through incubation with LPS for 24 h to induce inflammation, was incubated with investigated compounds for 24 h. All derivatives caused an increase in cell viability compared to the positive control (LPS) (Figure 4), which suggests they have a possibly good ability to reduce inflammation within cells. Some compounds improved cell viability above negative control, suggesting their proliferative activity. For compound **2b**, such improvement was observed in the lowest concentration with statistical significance. In comparison to positive control, when considering compounds that do not improve viability above negative control, derivative **3b** showed the best regenerative activity. For all compounds, the highest improvement of viability was reached using the lowest compound concentration, except for **3a**, where the best results were obtained when the highest concentration was applied.

#### 2.3.2. Level of ROS and Nitric Oxide Synthesis

**Figure 5:**
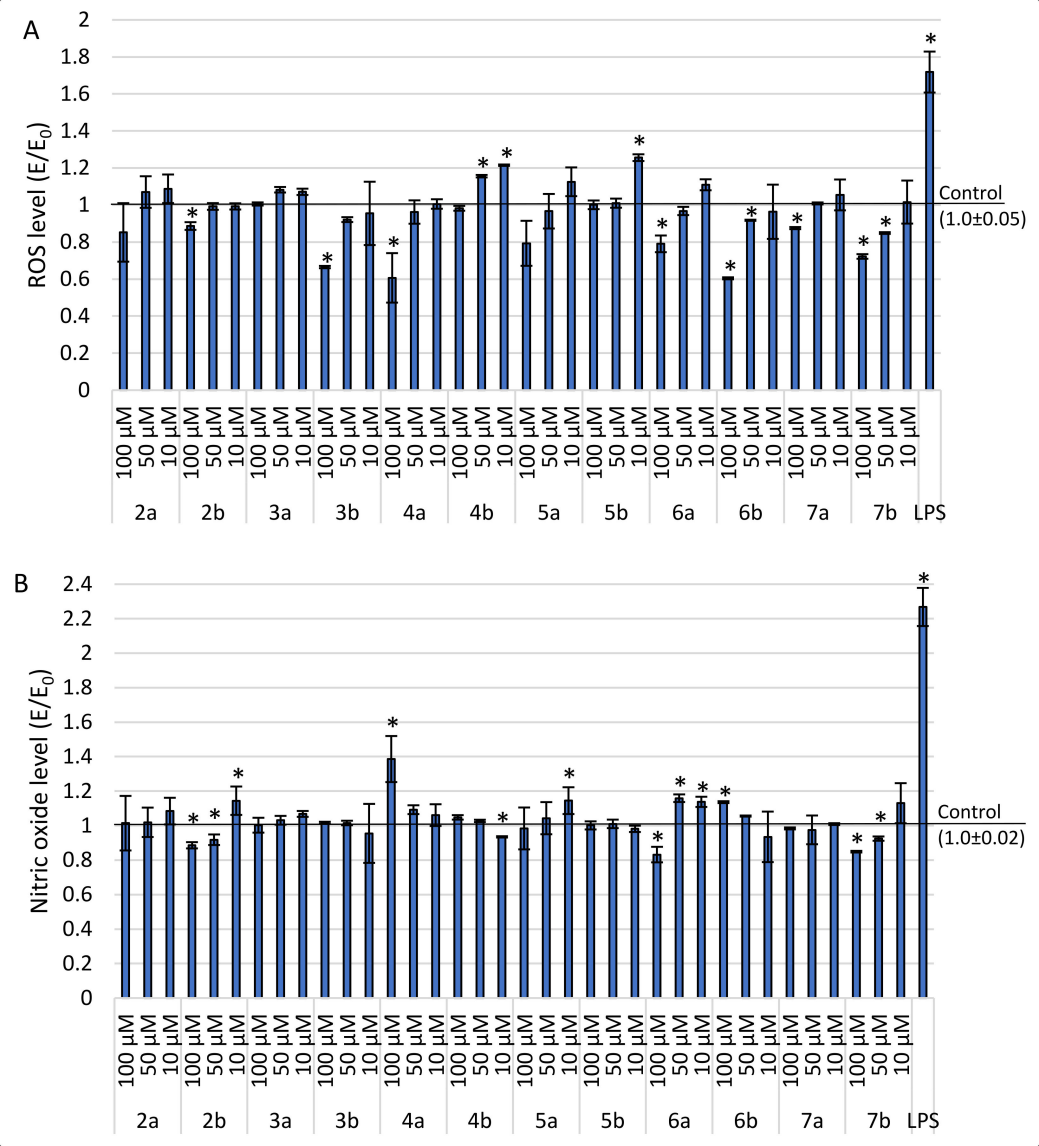
Impact of investigated compounds on THP-1 cell line incubated with LPS; (**A**)—DCF-DA assay, (**B**)—Griess assay, * *p* < 0.05—significant difference compared to control with 50 μg/mL LPS and without compounds.

Another study performed to evaluate potential anti-inflammatory activity of investigated derivatives was the estimation of accumulation of reactive oxygen species (ROS) and nitrite ions synthesis within LPS-damaged THP-1 cells after exposure to investigated compounds; for those, DCF-DA and Griess assays were performed, respectively (Figure 5).

There is a visible dependence showing that the higher the drug concentration is applied, the lower the level of ROS observed, although the differences are not extensive. All derivatives significantly decreased the ROS level compared to positive control (LPS). Compounds **4b** and **5b** appeared to be the weakest among all molecules in their lowest concentration with statistical significance. Compound **6b** at concentration of 100μM appeared to be the most potent in reducing oxidative stress (result statistically significant). All compounds were able to reduce the ROS level below the negative control in their highest concentrations.

All of the investigated compounds successfully reduced nitrosative stress compared to positive control (LPS). Derivatives **6a** and **7b** decreased the level of nitric oxide almost three times in their highest concentration, with statistical significance. There is no straight dependence between concentration and nitric oxide level, while for most compounds the activity is close to equal, except for **4a** which appeared to be visibly weakest in its highest concentration.

Taking into account the results above, we can confirm that all derivatives are able to reduce inflammation within cells.

### 2.4. In Silico Pharmacokinetic Prediction

All compounds underwent in silico pharmacokinetic properties prediction using SWISSADME server. None of them are assumed to cross blood–brain barrier and for all Mannich bases (**2a, b**–**4a, b**) the presumed GI absorption is high (Table 3). For compounds **5a**, **b**–**7a**, **b**, the oral availability, as well as their probability of bioavailability reaching >10% in rats (bioavailability score), is low. Those findings correspond with the Lipinski rule, which is fulfilled only for **2a, b**–**4a, b**. Veber filter is violated only for compounds **5b**, **6b** and **7b** (≥10 rotatable bonds) (Table 4). Low GI absorption and bioavailability parameters could presumably be associated with molecule size. In the case of compounds **5a, b**–**7a, b**, the linker between arylpiperazine pharmacophore and 1,3,4-oxadiazole moiety is more complex, which implies bigger molecule size and could therefore have a significant impact on absorption.

## 3. Materials and Methods

### 3.1. Chemistry

#### 3.1.1. Instruments and Chemicals

All solvents, reagents and chemicals used during experiments described in this paper were delivered by commercial suppliers (Alchem, Wrocław, Poland; Chemat, Gdańsk, Poland; Archem, Łany, Poland) and were used without further purification. Any dry solvents were received due to standard procedures. Reaction progress was monitored by thin-layer chromatography (TLC) technique, on TLC plates made of 60-254 silica gel and was visualized by UV light at 254/366 nm. Melting points of final compounds were determined on Electrothermal Mel-Temp 1101D apparatus (Cole-Parmer, Vernon Hills, IL, USA) using open capillary method, no correction needed. ^1^H NMR (300 MHz) and ^13^C NMR (75 MHz) spectra were recorded using Bruker 300 MHz NMR spectrometer (Bruker Analytische Messtechnik GmbH, Rheinstetten, Germany) in CDCl_3_/DMSO-d_6_, with tetramethylsilane (TMS) as an internal reference. Chemical shifts (δ) were reported in ppm. In order to record and read spectra, TopSpin 3.6.2. (Bruker Daltonik, GmbH, Bremen, Germany) program was used. FT-IR spectra were measured on Nicolet iS50 FT-IR Spectrometer (Thermo Fisher Scientific, Waltham, MA, USA). Frequencies were reported in cm^−1^. All samples were solid, and spectra were read by OMNIC Spectra 2.0 (Thermo Fisher Scientific, Waltham, MA, USA). Mass spectra (MS) were determined using Bruker Daltonics Compact ESI-mass spectrometer (Bruker Daltonik, GmbH, Bremen, Germany), operating in positive ion mode. The samples were dissolved in a methanol-chloroform mixture.

#### 3.1.2. Chemical Synthesis

All synthetic protocols for compounds **1a, b,** alongside with their experimental data, were reported previously [32].

General procedure for preparation of Mannich base type derivatives of **pyrrolo[3,4-*d*]pyridazinone 2a, b**–**4a, b.**

The proper 2-thioxo-3*H*-1,3,4-oxadiazole derivative (**1a** or **1b**) (0.001 mol), was suspended in absolute ethanol (30 mL) and 37% aqueous formaldehyde solution (0.01 mol) was added. The mixture was then stirred at room temperature for 15 min. Afterwards, adequate disubstituted phenylpiperazine (0.0015 mol) was added and stirring was continued for another few hours at room temperature. The mixture was left overnight. Obtained precipitate was filtered off, washed with ethanol and purified by crystallization from this solvent.

**2a** 3,5,7-Trimethyl-6-phenyl-1-[[4-[(4-(2,5-dimethylphenyl)piperazin-1-yl)methyl]-2-thioxo-1,3,4-oxadiazol-5-yl]methoxy]pyrrolo[3,4-*d*]pyridazin-4-one

Yield: 83.14%, m.p: 194–195 °C

FT-IR (selected lines, γ_max_, cm^−1^): 3053 (C-H arom.), 2952, 2833, 2809 (C-H aliph.), 1625 (C=N), 1549 (C=S)

^1^H NMR (300 MHz, CDCl_3_): δ = 2.20 (s, 3H, 7-CH_3_), 2.44 (s, 3H, 5-CH_3_), 2.92–2.95 (m, 8H, CH_2_-piperazine), 3.58 (s, 3H, 3-CH_3_), 5.09 (s, 2H, N-CH_2_), 5.34 (s, 2H, O-CH_2_), 6.77–6.81 (m, 2H, ArH), 7.03-7.05 (m, 1H, ArH), 7.18–7.7.21 (m, 2H, ArH) 7.54–7.56 (m, 3H, ArH);

^13^C NMR (75 MHz, CDCl_3_): δ = 11.42, 11.90, 17.40, 21.16, 37.11, 50.80, 51.65, 57.12, 70.56, 108.29, 112.04, 119.78, 123.98, 124.29, 127.78, 129.29, 129.390, 129.76, 130.86, 130.91, 163.12, 163.63, 147.88, 151.08, 157.63, 159.28, 178.87

HR-MS (m/z): calcd. for [L+H]+: 586.2595; found: 586.2591

**2b** 3,5,7-Trimethyl-6-butyl-1-[[4-[(4-(2,5-dimethylphenyl)piperazin-1-yl)methyl]-2-thioxo-1,3,4-oxadiazol-5-yl]methoxy]pyrrolo[3,4-*d*]pyridazin-4-one

Yield: 84.91%, m.p: 183–184 °C

FT-IR (selected lines, γ_max_, cm^−1^): 2974, 2949, 2869, 2820 (C-H aliph.), 1640 (C=N), 1547 (C=S)

^1^H NMR (300 MHz, CDCl_3_): δ = 0.95–0.99 (m, 3H, -CH_2_-CH_2_-CH_2_-CH_3_), 1.37–1.43 (m, 2H, -CH_2_-CH_2_-CH_2_-CH_3_), 1.62–1.65 (m, 2H, -CH_2_-CH_2_-CH_2_-CH_3_), 2.51 (s, 3H, 7-CH_3_), 2.69 (s, 3H, 5-CH_3_), 2.92–2.95 (m, 8H, CH_2_–piperazine), 3.54 (s, 3H, 3-CH_3_), 3.89–3.94 (m, 2H, -CH_2_-CH_2_-CH_2_-CH_3_), 5.09 (s, 2H, N-CH_2_), 5.30 (s, 2H, O-CH_2_), 6.79–6.81 (m, 2H, ArH), 7.04–7.06 (m, 1H, ArH);

^13^C NMR (75 MHz, CDCl_3_): δ = 10.662, 13.70, 17.42, 20.05, 21.17, 32.32, 37.05, 43.98, 50.79, 51.65, 57.05, 70.55, 108.02, 111.83, 119.79, 122.64, 123.98, 129.29, 130.91, 136.13, 147.79, 151.09, 157.69, 159.22, 178.88

HR-MS (m/z): calcd. for [L+H]+: 566.2908; found: 566.2901

**3a** 3,5,7-Trimethyl-6-phenyl-1-[[4-[(4-(2,4-difluorophenyl)piperazin-1-yl)methyl]-2-thioxo-1,3,4-oxadiazol-5-yl]methoxy]pyrrolo[3,4-*d*]pyridazin-4-one

Yield: 69.74%, m.p: 186–188 °C

FT-IR (selected lines, γ_max_, cm^−1^): 3054 (C-H arom.), 2925, 2835 (C-H aliph.), 1647 (C=N), 1503 (C=S)

^1^H NMR (300 MHz, CDCl_3_): δ = 2.28 (s, 3H, 7-CH_3_), 2.43 (s, 3H, 5-CH_3_), 3.00–3.01 (m, 8H, CH_2_–piperazine), 3.58 (s, 3H, 3-CH_3_), 5.08 (s, 2H, N-CH_2_), 5.32 (s, 2H, O-CH_2_), 6.75–6.92 (m, 3H, ArH), 7.18–7.7.21 (m, 2H, ArH) 7.53–7.56 (m, 3H, ArH);

^13^C NMR (75 MHz, CDCl_3_): δ = 11.41, 11.91, 37.12, 50.28, 50.82, 57.05, 70.26, 104.39, 104.74, 105.06, 108.27, 110.54, 110.87, 112.04, 119.54, 119.72, 124.32, 127.78, 129.38, 129.74, 130.84, 136.64, 147.86, 157.63, 159.29, 159.59, 178.85

HR-MS (m/z): calcd. for [L+H]+: 594.2093; found: 594.2096

**3b** 3,5,7-Trimethyl-6-butyl-1-[[4-[(4-(2,4-difluorophenyl)piperazin-1-yl)methyl]-2-thioxo-1,3,4-oxadiazol-5-yl]methoxy]pyrrolo[3,4-*d*]pyridazin-4-one

Yield: 83.02%, m.p: 202–205 °C

FT-IR (selected lines, γ_max_, cm^−1^): 2948, 2837 (C-H aliph.), 1621 (C=N), 1507 (C=S)

^1^H NMR (300 MHz, CDCl_3_): δ = 0.94–0.99 (m, 3H, -CH_2_-CH_2_-CH_2_-CH_3_), 1.37–1.42 (m, 2H, -CH_2_-CH_2_-CH_2_-CH_3_), 1.62–1.64 (m, 2H, -CH_2_-CH_2_-CH_2_-CH_3_), 2.51 (s, 3H, 7-CH_3_), 2.68 (s, 3H, 5-CH_3_), 3.00–3.02 (m, 8H, CH_2_–piperazine), 3.55 (s, 3H, 3-CH_3_), 3.88–3.93 (m, 2H, -CH_2_-CH_2_-CH_2_-CH_3_), 5.08 (s, 2H, N-CH_2_), 5.29 (s, 2H, O-CH_2_), 6.76–6.79 (m, 3H, ArH)

^13^C NMR (75 MHz, CDCl_3_): δ = 10.65, 11.27, 13.69, 20.04, 32.31, 37.07, 43.97, 50.28, 50.86, 59.99, 70.25, 104.39, 104.73, 105.06, 108.00, 110.54, 110.83, 110.88, 111.82, 119.60, 119.67, 119.72, 122.68, 129.28, 136.54, 147.79, 153.93, 157.67, 159.24, 178.86

HR-MS (m/z): calcd. for [L+H]+: 574.2406; found: 574.2390

**4a** 3,5,7-Trimethyl-6-phenyl-1-[[4-[(4-(3,4-dichlorophenyl)piperazin-1-yl)methyl]-2-thioxo-1,3,4-oxadiazol-5-yl]methoxy]pyrrolo[3,4-*d*]pyridazin-4-one

Yield: 75.71%, m.p: 205–207 °C

FT-IR (selected lines, γ_max_, cm^−1^): 3074 (C-H arom.), 2948, 2915, 2848 (C-H aliph.), 1624 (C=N), 1550 (C=S)

^1^H NMR (300 MHz, CDCl_3_): δ = 2.26 (s, 3H, 7-CH_3_), 2.43 (s, 3H, 5-CH_3_), 2.92–2.96 (m, 4H, CH_2_–piperazine), 3.14–3.17 (m, 4H, CH_2_–piperazine), 3.56 (s, 3H, 3-CH_3_), 5.07 (s, 2H, N-CH_2_), 5.31 (s, 2H, O-CH_2_), 6.68–6.72 (m, 1H, ArH), 6.90–6.92 (m, 1H, ArH), 7.17–7.7.20 (m, 3H, ArH) 7.54–7.56 (m, 3H, ArH);

^13^C NMR (75 MHz, CDCl_3_): δ = 11.42, 11.92, 3710, 48.81, 49.91, 57.02, 70.12, 108.23, 112.01, 115.71, 117.53, 122.58, 124.18, 127.76, 129.42, 129.77, 130.49, 130.90, 132.82, 136.59, 147.82, 150.49, 157.68, 159.28, 178.85

HR-MS (m/z): calcd. for [L+H]+: 626,1502; found: 626.1476

**4b** 3,5,7-Trimethyl-6-butyl-1-[[4-[(4-(3,4-dichlorophenyl)piperazin-1-yl)methyl]-2-thioxo-1,3,4-oxadiazol-5-yl]methoxy]pyrrolo[3,4-*d*]pyridazin-4-one

Yield: 93.41%, m.p: 190–192 °C

FT-IR (selected lines, γ_max_, cm^−1^): 2948, 2874, 2838 (C-H aliph.), 1619 (C=N), 1552 (C=S)

^1^H NMR (300 MHz, CDCl_3_): δ = 0.94–0.99 (m, 3H, -CH_2_-CH_2_-CH_2_-CH_3_), 1.34–1.42 (m, 2H, -CH_2_-CH_2_-CH_2_-CH_3_), 1.61–1.65 (m, 2H, -CH_2_-CH_2_-CH_2_-CH_3_), 2.49 (s, 3H, 7-CH_3_), 2.68 (s, 3H, 5-CH_3_), 2.92–2.96 (m, 4H, CH_2_–piperazine), 3.14–3.17 (m, 4H, CH_2_-piperazine), 3.52 (s, 3H, 3-CH_3_), 3.88–3.93 (m, 2H, -CH_2_-CH_2_-CH_2_-CH_3_), 5.07 (s, 2H, N-CH_2_), 5.28 (s, 2H, O-CH_2_), 6.69–6.73 (m, 1H, ArH), 6.92–6.93 (m, 1H, ArH), 7.24–7.27 (m, 1H, ArH);

^13^C NMR (75 MHz, CDCl_3_): δ = 10.65, 11.26, 20.04, 32.31, 37.03, 43.98, 48.81, 49.90, 56.94, 70.11, 107.97, 111.80, 115.69, 117.53, 122.56, 122.64, 129.32, 130.49, 132.82, 147.73, 150.49, 157.73, 159.21, 178.86

HR-MS (m/z): calcd. for [L+H]+: 606.1815; found: 606.1799

General procedure for preparation of ***S*-alkylated pyrrolo[3,4-*d*]pyridazinone derivatives 5a, b–7a, b**

The adequate pyrrolo[3,4-*d*]pyridazinone derivative **1a** or **1b** (0.001 mol) was suspended in 30 mL of anhydrous ethanol in a round bottom flask. Afterwards, 1 mL of sodium ethoxide (0.001 mol) was added and appropriate 1-(2-chloro-1-oxoethyl)-4-disubstituted phenylpiperazine (0.001 mol). Obtained mixture was refluxed for about 5 h. Progress of the reaction was monitored by TLC. Then the mixture was left overnight. Obtained precipitate was filtered off, washed with ethanol and purified by crystallization from this solvent.

**5a** 3,5,7-trimethyl-1-[[2-[2-oxo-2-(4-(2,5-dimethylphenyl)piperazin-1-yl)ethyl]sulfanyl-1,3,4-oxadiazol-5-yl]methoxy]-6-phenyl-pyrrolo[3,4-*d*]pyridazin-4-one

Yield: 52.17%, m.p: 159–161 °C

FT-IR (selected lines, γ_max_, cm^−1^): 2923, 2862, 2821 (C-H aliph.), 1646 (C=N)

^1^H NMR (300 MHz, CDCl_3_): δ = 2.26 (s, 3H, 7-CH_3_), 2.27 (s, 3H, Ar-CH_3_) 2.30 (s, 3H, Ar-CH_3_) 2.44 (s, 3H, 5-CH_3_) 2.89–2.94 (m, 4H, CH_2_-piperazine) 3.58 (s, 3H, 3-CH_3_) 3.70–3.79 (m, 4H, CH_2_-piperazine) 4,42 (s, 2H, S-CH_2_) 5.48 (s, 2H,O-CH_2_) 6.79–6.85 (m, 2H, ArH) 7.07–7.09 (m, 1H, ArH) 7.18–7.21 (m, 2H, ArH ), 7.53-7.55 (m, 3H, ArH)

^13^C NMR (75 MHz, CDCl_3_): δ = 11.42, 11.86, 17.36, 21.14, 37.10, 37.36, 42.95, 46.74, 51.51, 51.863, 57.02, 108.42, 112.07, 120.01, 124.24, 124.64, 127.79, 129.35, 129.42, 129.74, 130.72, 131.04, 136.35, 136.68, 148.05, 150.37, 159.31, 163.82, 164.60, 165.55

MS (m/z): calcd. for [L+H]+: 614.2544; found: 614.2504

**5b** 3,5,7-trimethyl-1-[[2-[2-oxo-2-(4-(2,5-dimethylphenyl)piperazin-1-yl)ethyl]sulfanyl-1,3,4-oxadiazol-5-yl]methoxy]-6-butyl-pyrrolo[3,4-*d*]pyridazin-4-one

Yield: 56.36%, m.p: 141–143 °C

FT-IR (selected lines, γ_max_, cm^−1^): 2923, 2862, 2821 (C-H aliph.), 1646 (C=N)

^1^H NMR (300 MHz, CDCl_3_): δ = 0.94–0.99 (m, 3H, -CH_2_-CH_2_-CH_2_-CH_3_), 1.35–1.42 (m, 2H, -CH_2_-CH_2_ CH_2_-CH_3_), 1.61–1.71 (m, 2H, -CH_2_-CH_2_-CH_2_-CH_3_), 2.274 (s, 3H, Ar-CH_3_), 2.30 (s, 3H, Ar-CH_3_), 2.49 (s, 3H, 7-CH_3_), 2.68 (s, 3H, 5-CH_3_) 2.90–2.95 (m, 4H, CH_2_-piperazine) 3.55 (s, 3H, 3-CH_3_) 3.70–3.80 (m, 4H, CH_2_-piperazine) 3.88–3.92 (m, 3H, -CH_2_-CH_2_-CH_2_-CH_3_) 4.42 (s, 2H, S-CH_2_) 5.45 (s, 2H,O-CH_2_), 6.79–6.85 (m, 2H, Ar-H), 7.07–7.10 (m, 1H, Ar-H)

^13^C NMR (75 MHz, CDCl_3_): δ = 10.65, 11.20, 13.70, 17.36, 20.05, 21.14, 32.32, 37.03, 37.35, 42.95, 43.95, 46.75, 51.52, 51.87, 56.95, 108.15, 111.84, 120.01, 122.60, 124.64, 129.14, 129.42, 131.04, 136.35, 147.97, 150.37, 159.26, 163.87, 164.61, 165.82

HR-MS (m/z): calcd. for [L+H]+: 594.2857; found: 594.2830

**6a** 3,5,7-trimethyl-1-[[2-[2-oxo-2-(4-(2,4-difluorophenyl)piperazin-1-yl)ethyl]sulfanyl-1,3,4-oxadiazol-5-yl]methoxy]-6-phenyl-pyrrolo[3,4-*d*]pyridazin-4-one

Yield: 87.98%, m.p: 198–201 °C

FT-IR (selected lines, γ_max_, cm^−1^): 3062 (C-H arom.), 2925, 2830 (C-H aliph.), 1643 (C=N)

^1^H NMR (300 MHz, CDCl_3_): δ = 2.25 (s, 3H, 7-CH_3_), 2.43 (s, 3H, 5-CH_3_) 3.00–3.07 (m, 4H, CH_2_-piperazine) 3.58 (s, 3H, 3-CH3) 3.73–3.83 (m, 4H, CH_2_-piperazine) 4.40 (s, 2H, S-CH_2_) 5.47 (s, 2H, O-CH*_2_*), 6.80–6.90 (m, 3H, Ar-H), 7.17–7.20 (m, 2H, Ar-H), 7.53–7.56 (m, 3H, Ar-H)

^13^C NMR (75 MHz, CDCl_3_): δ = 11.40, 11.84, 37.01, 37.08, 42.50, 46.33, 50.60, 51.08, 57.01, 104.60, 104.95, 108.42, 110.77, 111.05, 112.08, 120.07, 124.23, 127.79, 129.35, 129.74, 130.73, 136.68, 148.03, 159.30, 159.30, 163.89, 164.60, 165.40

HR-MS (m/z): calcd. for [L+H]+: 622.2043; found: 622.2019; calcd. for [L+Na]: 644.1862, found: 644.1835

**6b** 3,5,7-trimethyl-1-[[2-[2-oxo-2-(4-(2,4-difluorophenyl)piperazin-1-yl)ethyl]sulfanyl-1,3,4-oxadiazol-5-yl]methoxy]-6-butyl-pyrrolo[3,4-*d*]pyridazin-4-one

Yield: 48.88%, m.p: 147–149 °C

FT-IR (selected lines, γ_max_, cm^−1^): 2960, 2935, 2918, 2871 (C-H aliph.), 1656 (C=N)

^1^H NMR (300 MHz, CDCl_3_): δ = 0.94–0.99 (m, 3H, -CH_2_-CH_2_-CH_2_-CH_3_), 1.37–1.39 (m, 2H, -CH_2_-CH_2_ CH_2_-CH_3_), 1.64–1.67 (m, 2H, -CH_2_-CH_2_-CH_2_-CH_3_), 2.49 (s, 3H, 7-CH3), 2.68 (s, 3H, 5-CH_3_) 3.00–3.09 (m, 4H, CH_2_-piperazine) 3.54 (s, 3H, 3-CH_3_) 3.72–3.84 (m, 4H, CH_2_-piperazine) 3.87–3.93 (m, 3H, -CH_2_-CH_2_-CH_2_-CH_3_) 4.40 (s, 2H, S-CH_2_) 5.44 (s, 2H, O-CH_2_), 6.80–6.84 (m, 2H, Ar-H), 6.87–6.90 (m, 1H, Ar-H)

^13^C NMR (75 MHz, CDCl_3_): δ = 10.64, 11.19, 13.688, 20.04, 32.32, 37.02, 42.50, 43.95, 46.34, 50.58, 51.09, 56.93, 104.60, 104.92, 105.27, 108.15, 110.81, 111.09, 111.85, 120.14, 122.58, 129.15, 135.88, 147.95, 159.24, 163.64, 164.61, 165.38

MS (m/z): calcd. for [L+Na]+: 624.2175; found: 624.2133

**7a** 3,5,7-trimethyl-1-[[2-[2-oxo-2-(4-(3,4-dichloro)piperazin-1-yl)ethyl]sulfanyl-1,3,4-oxadiazol-5-yl]methoxy]-6-phenyl-pyrrolo[3,4-*d*]pyridazin-4-one

Yield: 61.00%, m.p: 214–217 °C

FT-IR (selected lines, γ_max_, cm^−1^): 2925, 2840 (C-H aliph.), 1637 (C=N)

^1^H NMR (300 MHz, CDCl_3_): δ = 2.250 (s, 3H, 7-CH_3_), 2.43 (s, 3H, 5-CH_3_) 3.17–3.22 (m, 2H, CH_2_-piperazine) 3.58 (s, 3H, 3-CH_3_) 3.74–3.80 (m, 4H, CH_2_-piperazine) 4.38 (s, 2H, S-CH_2_) 5.48 (s, 4H, O-CH_2_), 6.72–6.76 (m, 1H, Ar-H), 6.96–6.97 (m, 1H, Ar-H) 7.18–7.20 (m, 2H, Ar-H), 7.28–7.32 (m, 1H, Ar-H) 7.53-7.56 (m, 3H, Ar-H)

^13^C NMR (75 MHz, CDCl_3_): δ = 11.41, 11.85, 36.72, 37.08, 41.99, 45.78, 48.78, 49.11, 56.99, 108.40, 112.06, 116.00, 118.06, 123.54, 124.23, 127.78, 129.36, 129.74, 130.66, 133.04, 136.66, 148.02, 150.00, 159.30, 163.95, 164.69, 165.300

MS (m/z): calcd. for [L+H]+: 654.1452 found: 654.1416

**7b** 3,5,7-trimethyl-1-[[2-[2-oxo-2-(4-(3,4-dichlorophenyl)piperazin-1-yl)ethyl]sulfanyl-1,3,4-oxadiazol-5-yl]methoxy]-6-butyl-pyrrolo[3,4-*d*]pyridazin-4-one

Yield: 82.00%, m.p: 167–168 °C

FT-IR (selected lines, γ_max_, cm^−1^): 2961, 2920, 2850 (C-H aliph.), 1630 (C=N)

^1^H NMR (300 MHz, CDCl_3_): δ= 0.94-0.99 (m, 3H, -CH_2_-CH_2_-CH_2_-CH_3_), 1.37–1.39 (m, 2H, -CH_2_-CH_2_ CH_2_-CH_3_), 1.60–1.69 (m, 2H, -CH_2_-CH_2_-CH_2_-CH_3_), 2.49 (s, 3H, 7-CH_3_), 2.68 (s, 3H, 5-CH_3_) 3.17–3.23 (m, 4H, CH_2_-piperazine) 3.54 (s, 3H, 3-CH_3_) 3.74–3.80 (m, 4H, CH_2_-piperazine) 3.87–3.92 (m, 3H, -CH_2_-CH_2_-CH_2_-CH_3_) 4.38 (s, 2H, S-CH_2_) 5.44 (s, 2H, O-CH_2_), 6.73–6.77 (m, 2H, Ar-H), 6.96–6.97 (m, 1H, Ar-H)

^13^C NMR (75 MHz, CDCl_3_): δ = 10.65, 11.19, 13.69, 20.04, 32.32, 36.69, 37.02, 42.00, 43.94, 45.79, 48.78, 49.10, 56.92, 108.13, 111.85, 115.99, 118.05, 122.58, 123.52, 129.16, 130.67, 133.04, 147.93, 150.01, 159.24, 164.01, 164.71, 165.26

MS (m/z): calcd. for [L+H]+: 634.1765; found: 634.1733

### 3.2. Cell Line

In this study, two different cell lines were used—NHDF and THP-1.

Normal human dermal fibroblasts (NHDF) (Lonza, Basel, Switzerland), adherent regular skin fibroblasts line, were incubated in 37 °C, 5% CO_2_, 95% humidity and passaged twice a week. Cells were evaluated microscopically and, depending on confluency, either passaged or the medium was replaced. This line was used only for estimation of cytotoxicity of new compounds.

THP-1 cells were received from monocytic leukemia patient. This line was incubated as suspension in the same conditions as NHDF cells and was used to determine the ability of investigated derivatives to reduce inflammation.

### 3.3. Cell Culture Media

NHDF cells were cultured in Dulbecco Modified Eagle Medium (DMEM) without phenol red, with 10% fetal bovine serum (FBS), 100 ug/mL gentamicin, 1.25 ug/mL amphotericin B, 2 mM L-glutamine.

THP-1 cells were cultured in RPMI 1640 medium with 10% FBS, 100 ug/mL gentamicin and 2mM L-glutamine.

### 3.4. Tested Compounds

All compounds were synthesized and provided by Department of Chemistry of Drugs, Wrocław Medical University. After received, DMSO was used to dissolve the derivatives to achieve 10 mM stock concentration. Those solutions were then used to prepare desired final concentrations of 100 μM, 50 μM and 10 μM for each derivative, respectively. LPS (Sigma-Aldrich, Saint Louis, MO, USA, cat. No. L2630) acquired from E. coli, was brought to stock concentration (1 mM) by dissolving in distilled water. Eventually, final concentration of 50 μM was obtained using primary medium.

Once dissolved, all compounds were stored at −20 °C up to 6 months.

### 3.5. Experimental Design

NHDF cells were put into 96-well plates (density 10,000 cells/well) and left overnight to adhere. After removing the suprenatant, the cells were incubated with tested compounds in 5% CO_2_, 37 °C, 24 h for MTT assay. This line was used only for cytotoxicity estimation.

THP-1 cell line, cultured as described above, was centrifuged (1000× *g*, 5 min), then counted and put into plates consisting of 96 wells with density of 40,000 cells/well. Afterwards, the plates were left overnight for the cells to precipitate and then freshly prepared LPS was added to reach concentration of 50 μg/mL. After 24 h of inflammation activation, tested compounds were added. This line was used for estimation of anti-inflammatory activity of compounds.

One control was used for NHDF (negative) and two for THP-1 (negative and positive). Neither negative nor positive control included tested compounds. Cells incubated in primary medium were negative control, while the positive control consisted of cells with LPS at concentration of 50 μg/mL.

To detect possible cytotoxicity of investigated compounds, MTT assay was performed using NHDF cell lines. Afterwards, having performed cyclooxygenase inhibition studies, in order to measure anti-inflammatory activity of the compounds within cells, MTT assay on THP-1 line was carried out. This line was previously exposed to LPS and incubated for 24 h to induce inflammation. In addition, estimation of reactive oxygen species was conducted in DCF-DA assay. Griess assay was carried out in order to determine nitric oxide level.

### 3.6. MTT Assay

In order to determine the influence of evaluated compounds on cells viability, MTT assay was performed using NHDF and THP-1 lines. For NHDF line, after incubating the cells with investigated derivatives (5% CO_2_, 37 °C, 2 h), supernatant was eliminated and MTT solution in MEM (1mg/mL) was poured into each well to finally incubate the plates (5% CO_2_, 37 °C, 2 h). Afterwards, the medium was removed and obtained formazan crystals were dissolved using 100 μL of isopropanol and left for 30 min. For THP-1 line, after incubation with compounds (5% CO_2_, 37 °C, 4 h), very little amount of supernatant was carefully removed and 5 mg/mL MTT in MEM was added, followed by lysis buffer. In order to measure the absorbance, Varioskan LUX microplate reader (Thermo Scientific, USA) was used at wavelength of 570 nm. 

### 3.7. Level of Reactive Oxygen Species (ROS)—DCF-DA Assay

To estimate the level of ROS, DCF-DA assay was performed. Having incubated THP-1 cell line with investigated derivatives (24 h), culture medium was eliminated following cells washing using PBS. Afterwards, after adding DCF-DA solution in MEM (without serum and phenol red), incubation of cultures was carried out (37 °C, 1 h). ROS level was evaluated fluorimetrically on Varioskan Lux microplate reader (Thermo Scientific, USA) with 485 nm fluorescence excitation and 535 nm emission.

### 3.8. Level of Nitrate Ions Synthesis—Griess Assay

Griess Assay procedure was performed in order to estimate nitrate ions synthesis in THP-1 cell line. Firstly, cells were incubated with investigated compounds (1 h) and then, after transferring suprenatant (50 μL) to new plate, 50 μL of Griess reagent was added (1% sulfanilamide in 5% phosphoric acid solution and 0.1% N-(1-Naphthyl)etyhlenediamine dihydrochloride, 1:1 *v/v* mixture). The plate was put in the dark at RT and left for 20 min. Level of nitrite was estimated using Varioskan LUX microplate reader (Thermo Scientific, USA) with 548 nm wavelength.

### 3.9. Statistical Analysis

Any outcome result in biological assays was presented as mean ± SEM (standard error of the mean, relative to respective control (E/E_0_), E—measured sample, E_0_—negative control result). In MTT assay for NHDF cell line, the results for healthy cell culture without any damage factor was the negative control. For THP-1 lines, regenerative effect was investigated. Therefore, the positive control was carried out through incubation in presence of harmful factor LPS.

Considering statistical significance, the analyses were conducted using ANOVA parametric test and also post hoc (Scheffé’s method). The significance level was * *p* < 0.05.

### 3.10. Molecular Modeling

The structures of all designed compounds were optimized at the PM6 level of theory using Gaussian 09 package [43,44]. The PCM model (polarizable continuum model) was adopted in order to take into account the solvent effect [45]. AutoDock 4.2 program and the standard protocol were to predict the binding mode of compounds to both COX-1 and COX-2 isoenzymes [46]. The protein structures were downloaded from Protein Data Bank. As a molecular target, COX-1 (PDB ID:4O1Z) and COX-2 (PDB ID:4M11) crystals co-crystalized with meloxicam were used [42]. The validation of docking procedure was performed by docking of meloxicam into the enzyme and comparison of its position with the crystal. The root mean square deviation (RMSD) was calculated to estimate the accuracy of docking prediction on the LigRMSD web server [47]. The binding mode of inhibitors was correctly predicted when its RMSD was found to be less than 1 Å. The protein and ligand preparation and docking procedure were described in detail in previous studies [48,49,50]. Lamarckian genetic algorithm with local search was employed with a total of 200 runs for each binding site. Previous studies have shown that it is the most efficient and reliable algorithm of AutoDock 4.2 [51]. The obtained results were visualized using a Chimera and a BIOVIA Discovery Studio visualizer [52].

## 4. Conclusions

In the current study, we reported the synthesis and complex in vitro and in silico evaluation of novel 1,3,4-oxadiazole based derivatives of pyrrolo[3,4-*d*]pyridazinone with double-substituted arylpiperazine pharmacophore. New molecules were designed as potential cyclooxygenase inhibitors and, in fact, revealed such activity in both in vitro assay and molecular docking study. Structural modification performed on our derivatives was inspired by our former investigations [31,32] and aimed to enhance the COX-2 selectivity of new molecules. In practice, all 12 compounds generally showed promising but similar inhibitory activity to both isoenzymes of cyclooxygenase.

As it has been mentioned already, previously reported Mannich base type derivatives with non or monosubstituted arylpiperazine moiety revealed promising COX-2 inhibitory activity. Investigated compounds acted as specific or selective COX-2 inhibitors, with COX-2/COX-1 selectivity ratio better than the Meloxicam used as the reference [32]. On the other hand, compounds with arylpiperazine pharmacophore introduced via the 2-oxoethylene linker were found to be selective to the COX-2 isoform, although their activity was lower [31]. The introduction of this extended, flexible linker was probably responsible for the COX inhibitory profile.

Unexpectedly, the current study demonstrated that the abovementioned structure–activity relationships were not supported by the results gained for 12 derivatives described in this paper. The presence of disubstituted phenyl ring in arylpiperazine pharmacophore drastically modified profile of action of investigated compounds. We can conclude that, in the context of bioactivity, the nature of the linker does not seem to be as important as it was thought to be. Although the results did not fulfil our expectations about COX-2 selectivity of new molecules, the obtained data are crucial and helpful for estimation of structure–activity relationships in the group of 1,3,4-oxadiazole based derivatives of pyrrolo[3,4-*d*]pyridazinone.

It is worth noticing that all investigated compounds did not show cytotoxicity and confirmed their potential anti-inflammatory activity in performed additional in vitro investigations. All derivatives increased cell viability in MTT assay and were able to reduce induced oxidative and nitrosative stress. These findings confirm that reported molecules are able to reduce inflammation within cells. In the case of the mentioned results, we were unable to register any significant differences between the activity of particular molecules.

When considering the performed pharmacokinetic prediction, we can point out the meaningful difference in potential GI absorption and bioavailability between the compounds from the series **2a**, **b**–**4a**, **b** and **5a**, **b**–**7a**, **b**. Mannich base type derivatives (**2a**, **b**–**4a**, **b**), due to lower molecular weight and size, would probably show better membrane permeability.

Summing up, we have reported the synthesis and biological evaluation of 12 new, promising cyclooxygenase inhibitors based on the pyrrolo[3,4-*d*]pyridazinone scaffold. All compounds were not toxic and showed very similar activity in the performed in vitro experiments and also in the molecular docking studies. We were unable to achieve the intended goal of receiving potent and selective COX-2 inhibitors. Inspired by earlier instructive research, we will continue our efforts in this field of medicinal chemistry.

## Figures and Tables

**Table 1:** IC_50_ values determined for COX-1 and COX-2; data are shown as standard deviation (SD).

Compound	IC_50_ [µM] (SD)		COX-2/COX-1 Selectivity Ratio
	COX-1	COX-2	
**1a** **1b** **2a**	130.5 (18.5) 138.1 (12.7) 50.3 (0.01)	43.6 (1.3) 63.4 (7.2) 49.7 (0.03)	0.33 0.46 0.99
**2b**	50.0 (0.01)	49.4 (0.02)	0.99
**3a**	50.3 (0.01)	49.7 (0.02)	0.99
**3b**	54.3 (0.02)	56.0 (0.01)	1.03
**4a**	50.2 (0.01)	49.2 (0.01)	0.98
**4b**	56.8 (0.01)	57.3 (0.01)	1.01
**5a**	53.2 (0.02)	50.1 (0.08)	0.94
**5b**	51.8 (0.03)	53.8 (0.01)	1.04
**6a**	52.3 (0.01)	50.2 (0.02)	0.96
**6b**	51.1 (0.01)	50.1 (0.01)	0.98
**7a**	51.6 (0.02)	50.3 (0.02)	0.97
**7b**	50.9 (0.04)	49.5 (0.02)	0.97
Meloxicam	83.7 (0.03)	59.2 (0.06)	0.71
Celecoxib	56 (0.1)	0.30 (0.08)	0.005
Diclofenac	3.5 (0.04)	16.6 (0.03)	4.74

**Table 2:** Binding energies of designed compounds to (a) COX-1 and (b) COX-2 obtained from molecular docking (ΔG_bind_—free energy of binding; ΔE_int_—intermolecular interaction energy; Δ*E_1_* = Δ*E_vdw_* + Δ*E_hbond_* + Δ*E_desolv_*; Δ*E_2_* = Δ*E_el_* [kcal/mol]).

**a**	**ΔG_bind_**	**ΔE_int_**	**ΔE_1_**	**ΔE_2_**
**1a**	−7.1	−9.2	−9.2	0.0
**1b**	−6.9	−9.0	−8.9	−0.1
**2a**	−8.4	−10.5	−10.4	−0.1
**2b**	−7.5	−9.6	−9.4	−0.2
**3a**	−10.2	−12.9	−12.8	−0.1
**3b**	−9.6	−11.2	−11.2	0.0
**4a**	−8.6	−10.0	−10.1	0.1
**4b**	−8.4	−10.8	−11.1	0.3
**5a**	−7.1	−9.1	−9.1	0.0
**5b**	−9.4	−12.4	−12.4	0.0
**6a**	−11.2	−13.3	−13.2	−0.1
**6b**	−10.7	−13.1	−13.1	0.0
**7a**	−9.7	−11.1	−11.3	0.2
**7b**	−9.3	−11.3	−11.3	0.0
Meloxicam	−8.6	−10.0	−9.9	−0.1
Diclofenac	−9.7	−10.9	−10.9	0.0
**b**	**ΔG_bind_**	**ΔE_int_**	**ΔE_1_**	**ΔE_2_**
**1a**	−12.5	−13.7	−13.7	0.0
**1b**	−12.7	−13.8	−13.8	0.0
**2a**	−10.8	−13.0	−13.0	0.0
**2b**	−11.7	−13.8	−13.7	−0.1
**3a**	−11.0	−13.1	−13.1	0.0
**3b**	−12.9	−14.7	−14.7	0.0
**4a**	−10.3	−13.0	−13.0	0.0
**4b**	−11.3	−10.6	−10.7	0.1
**5a**	−11.6	−14.2	−14.2	0.0
**5b**	−11.4	−13.3	−13.3	0.0
**6a**	−11.6	−14.2	−14.2	0.0
**6b**	−10.9	−13.7	−13.7	0.0
**7a**	−11.5	−14.1	−14.1	0.0
**7b**	−11.8	−13.1	−13.1	0.0
Meloxicam	−9.9	−10.0	−9.8	−0.2
Diclofenac	−10.9	−11.0	−11.0	0.0

**Table 3:** Predicted pharmacokinetic properties of investigated compounds using SWISSADME server (accessed on 18 June 2021).

Compound	Pharmacokinetics				
	MW	Log P_o/w_	GI Absorption	BBB Permeant	P-gp Substrate
**2a**	585.72 g/mol	4.63	High	No	Yes
**2b**	565.73 g/mol	4.36	High	No	Yes
**3a**	593.65 g/mol	4.40	High	No	Yes
**3b**	573.66 g/mol	4.39	High	No	Yes
**4a**	626.56 g/mol	4.86	High	No	Yes
**4b**	606.57 g/mol	4.81	High	No	Yes
**5a**	613.73 g/mol	4.19	Low	No	Yes
**5b**	593.74 g/mol	4.02	Low	No	Yes
**6a**	621.66 g/mol	4.09	Low	No	Yes
**6b**	601.67 g/mol	4.05	Low	No	Yes
**7a**	654.57 g/mol	4.51	Low	No	Yes
**7b**	634.58 g/mol	4.41	Low	No	Yes

**Table 4:** Predicted druglikeness of investigated compounds using SWISSADME server (accessed 18 June 2021).

Compound	Druglikeness			
	Lipinski	Veber	Bioavailability Score	TPSA
**2a**	Yes; 1 violation	Yes	0.55	118.58 Å^2^
**2b**	Yes; 1 violation	Yes	0.55	118.58 Å^2^
**3a**	Yes; 1 violation	Yes	0.55	118.58 Å^2^
**3b**	Yes; 1 violation	Yes	0.55	118.58 Å^2^
**4a**	Yes; 1 violation	Yes	0.55	118.58 Å^2^
**4b**	Yes; 1 violation	Yes	0.55	118.58 Å^2^
**5a**	No; 2 violations	Yes	0.17	136.82 Å^2^
**5b**	No; 2 violations	No; 1 violation	0.17	136.82 Å^2^
**6a**	No; 2 violations	Yes	0.17	136.82 Å^2^
**6b**	No; 2 violations	No; 1 violation	0.17	136.82 Å^2^
**7a**	No; 2 violations	Yes	0.17	136.82 Å^2^
**7b**	No; 2 violations	No; 1 violation	0.17	136.82 Å^2^

## Supplementary Materials

The following are available online at https://www.mdpi.com/article/10.3390/ijms22179130/s1.
