# Supplementary material for: In Vitro and In Silico Evaluation of New 1,3,4-Oxadiazole Derivatives of Pyrrolo[3,4-d]pyridazinone as Promising Cyclooxygenase Inhibitors
Source: Int J Mol Sci. 2021 Aug 24;22(17):9130. doi: 10.3390/ijms22179130 (PMC8431030; doi:10.3390/ijms22179130)
Supplement: Supplementary file 1 [file ijms-22-09130-s001.zip › ijms-1321379-supplementary.pdf]

SUPPLEMENTARY DATA

In vitro and *in silico* evaluation of new oxadiazole derivatives of pyrrolo[3,4-d]pyridazinone as promising cyclooxygenase inhibitors

Krzysztof Peregrym[1], Łukasz Szczukowski[1], Benita Wiatrak[2], Katarzyna Potyrak[2], Żaneta Czyżnikowska[3] and Piotr Świątek[1]

[1] Department of Medicinal Chemistry, Faculty of Pharmacy, Wroclaw Medical University, Borowska 211, 50-556 Wroclaw, Poland; krzysztof.peregrym@student.umed.wroc.pl (K.P.), lukasz.szczukowski@umed.wroc.pl (Ł.S.), piotr.swiatek@umed.wroc.pl (P.Ś.)
[2] Department of Pharmacology, Faculty of Medicine, Wroclaw Medical University, Mikulicza-Radeckiego 2, 50-345 Wroclaw, Poland; benita.wiatrak@umed.wroc.pl (B.W.), katarzyna.potyrak@student.umed.wroc.pl (K.P.)
[3] Department of Inorganic Chemistry, Wroclaw Medical University, Borowska 211, 50-556 Wroclaw, Poland; zaneta.czyznikowska@umed.wroc.pl (Ż.C.)
* Correspondence: piotr.swiatek@umed.wroc.pl ; Tel.: +48 71 784 03 91

Table of contents

Table S1. Structures of new compounds 2
Table S2. NMR spectra of new compounds 5
Table S3. IR spectra of new compounds 17
Table S4. Mass spectra of new compounds 23
Table S5. The 2D intermolecular interactions of investigated compounds in the active site of COX 29
Table S6. Binding mode of investigated compounds to COX-1 41
Table S7. Binding mode of investigated compounds to COX-2 43

Table S1. Structures of new compounds

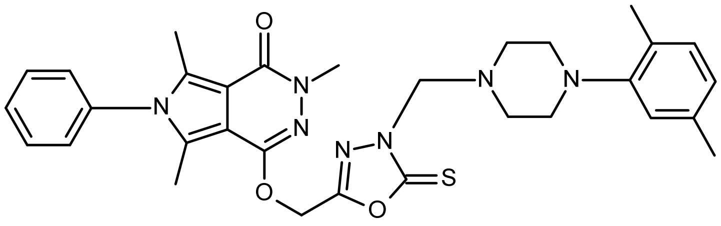
2a
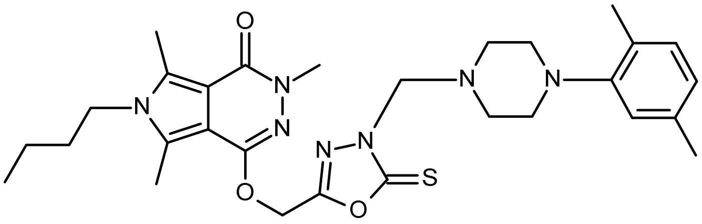
2b
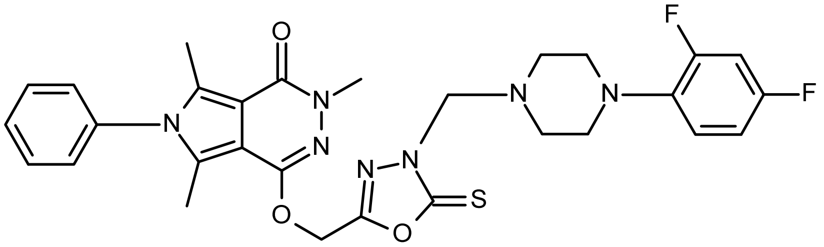
3a
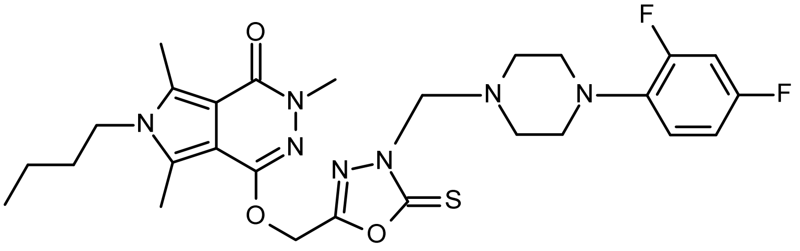
3b
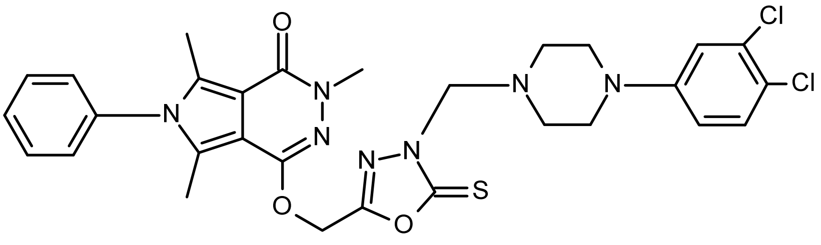
4a

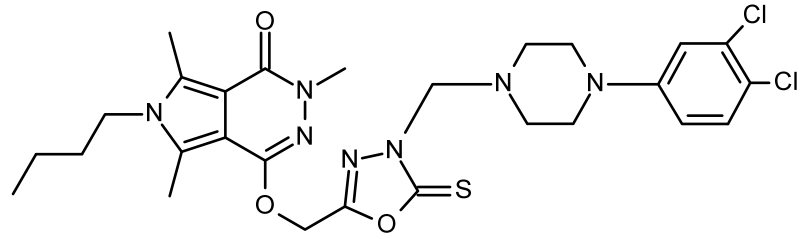O<br>N<br>N<br>N<br>O<br>N<br>N<br>N<br>O<br>S<br>N<br>N<br>Cl<br>Cl
4b
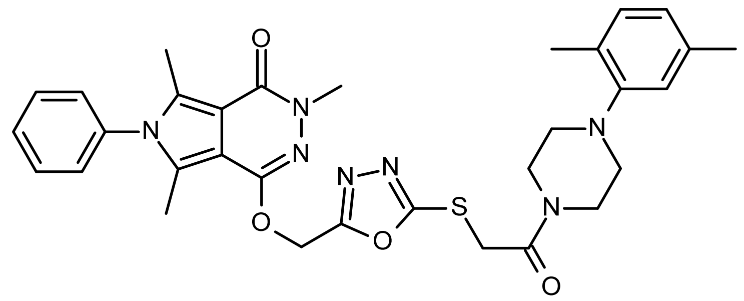O<br>N<br>N<br>N<br>O<br>N<br>N<br>O<br>S<br>N<br>N<br>O
5a
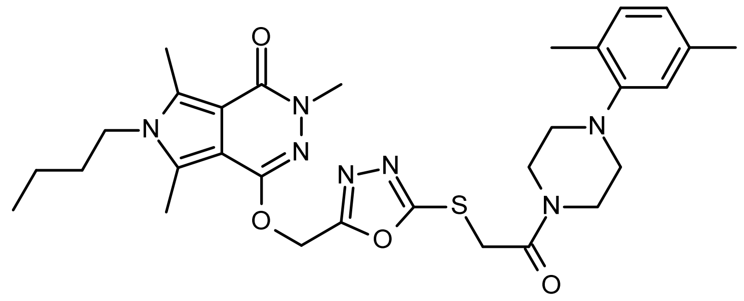O<br>N<br>N<br>N<br>O<br>N<br>N<br>O<br>S<br>N<br>N<br>O
5b
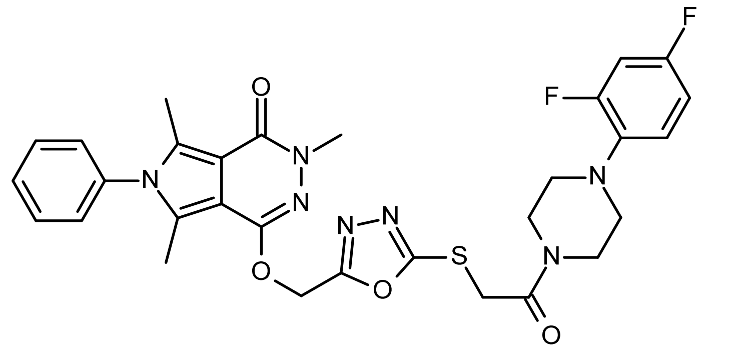F<br>F<br>O<br>N<br>N<br>N<br>O<br>N<br>N<br>O<br>S<br>N<br>N<br>O
6a
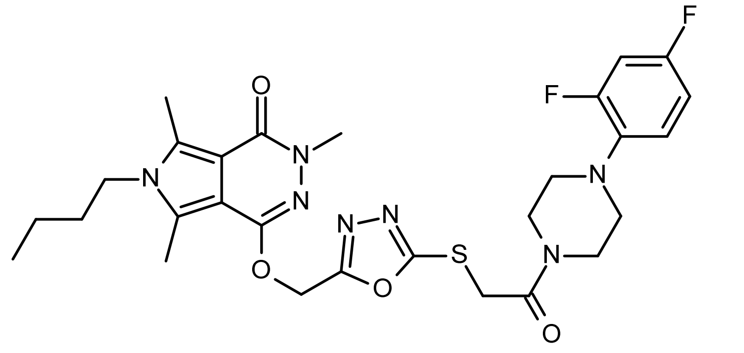F<br>F<br>O<br>N<br>N<br>N<br>O<br>N<br>N<br>O<br>S<br>N<br>N<br>O
6b

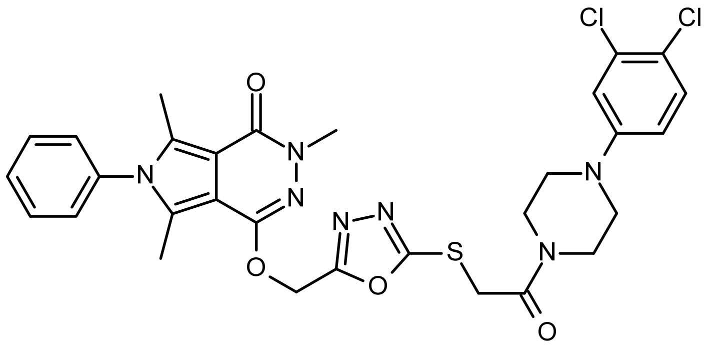
7a
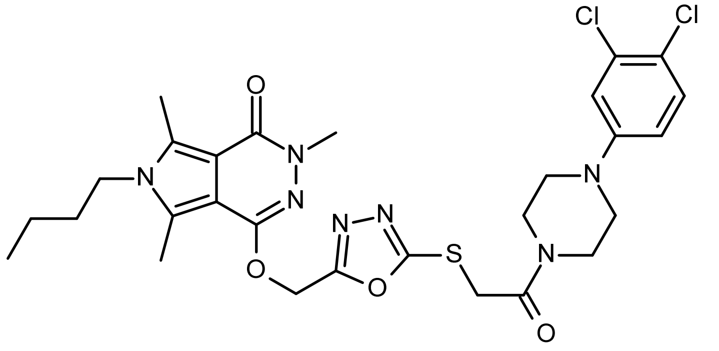
7b

Table S2. NMR spectra of new compounds

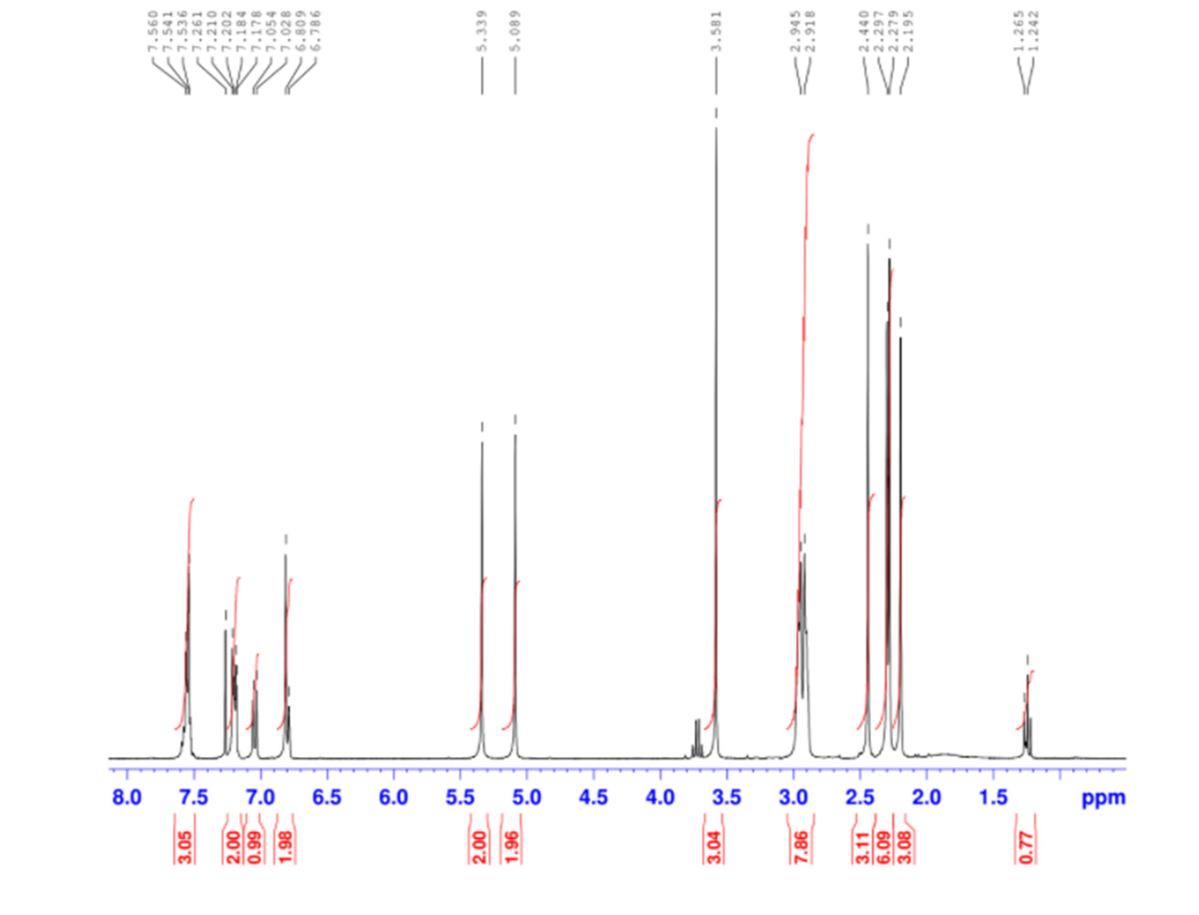

2a

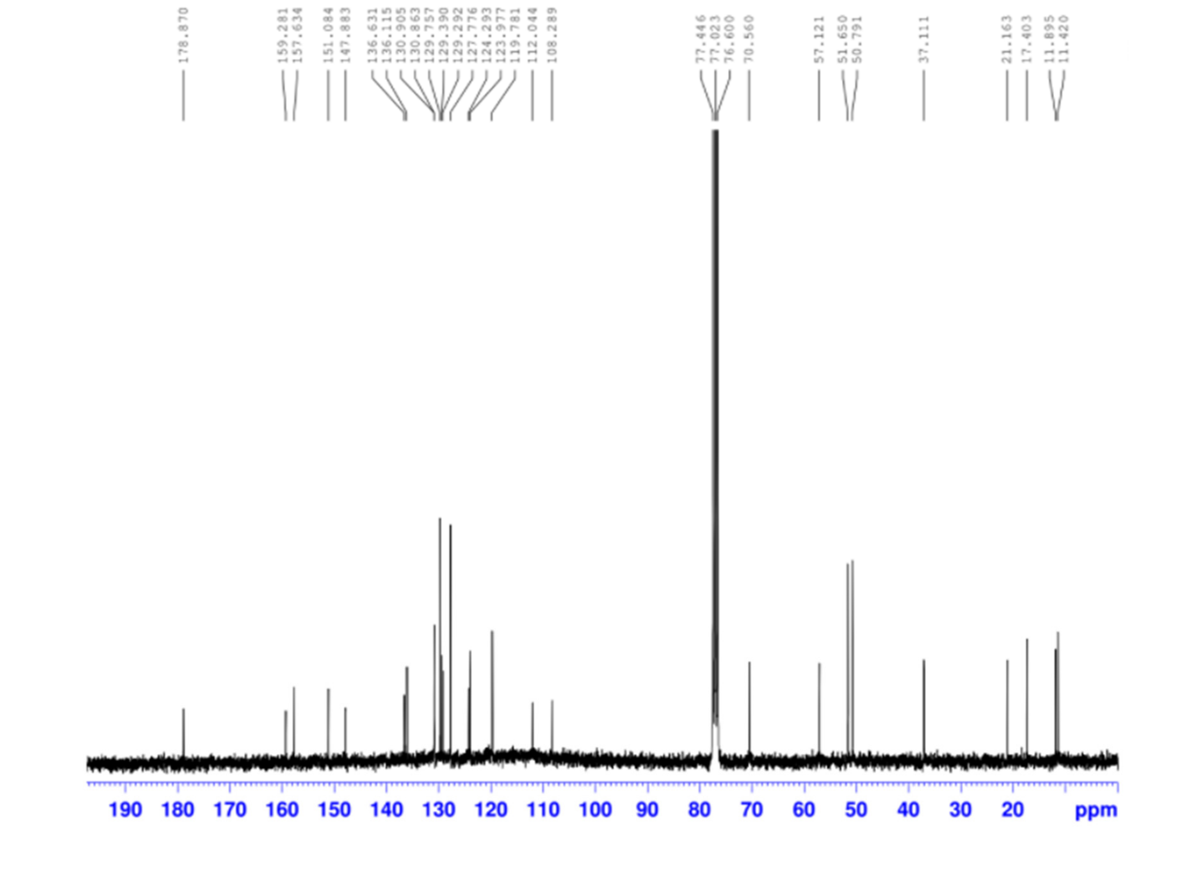

2a

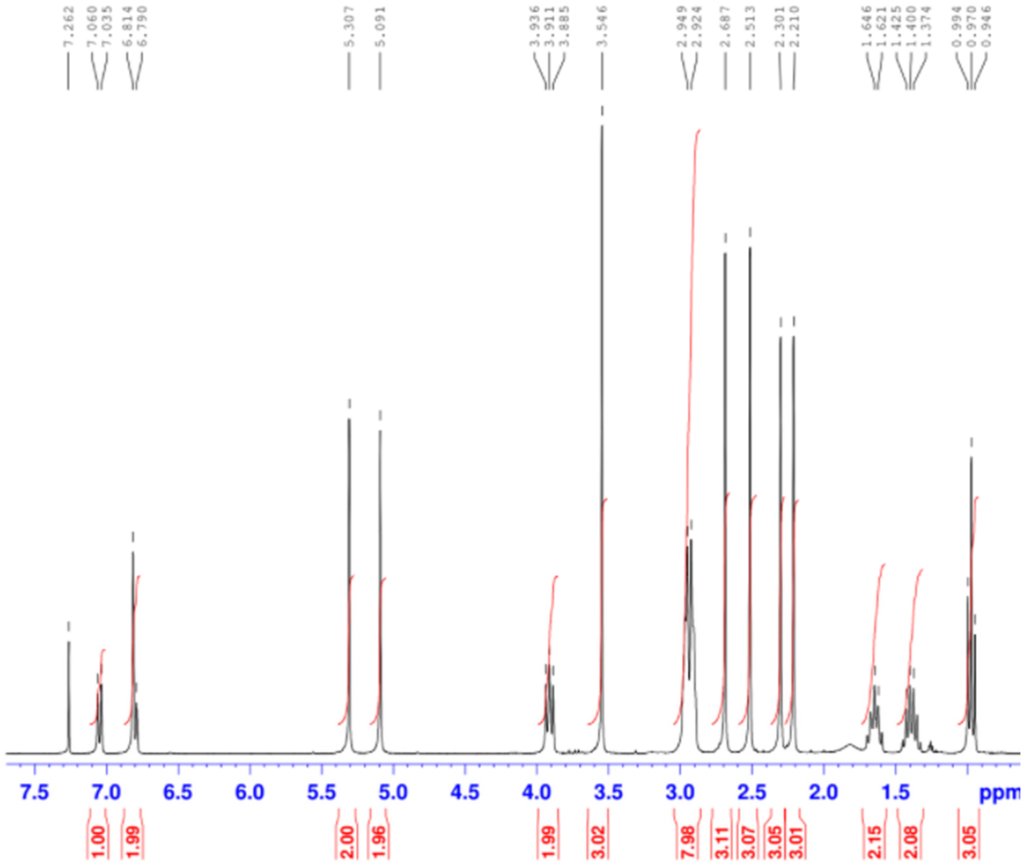
7.262
7.060
7.035
6.814
6.790
5.307
5.091
3.936
3.911
3.885
3.546
2.949
2.924
2.687
2.513
2.301
2.210
1.646
1.621
1.425
1.400
1.374
0.994
0.970
0.946
7.5 7.0 6.5 6.0 5.5 5.0 4.5 4.0 3.5 3.0 2.5 2.0 1.5 ppm
1.00 1.99 2.00 1.96 1.99 3.02 7.98 3.11 3.07 3.05 3.01 2.15 2.08 3.05

2b

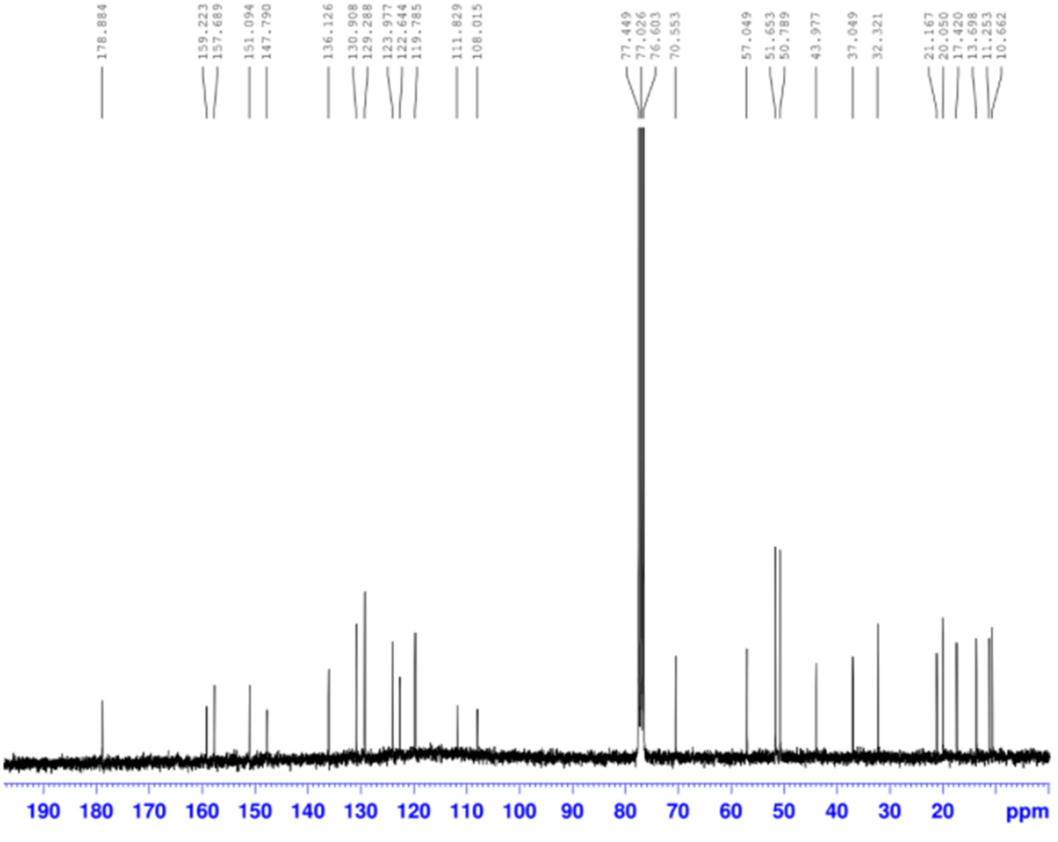
178.884
159.223
157.689
151.094
147.790
136.126
130.908
129.288
123.977
122.644
119.785
111.829
108.015
77.449
77.026
76.603
70.553
57.049
51.653
50.789
43.977
37.049
32.321
21.167
20.050
17.420
13.698
11.253
10.662
190 180 170 160 150 140 130 120 110 100 90 80 70 60 50 40 30 20 ppm

2b

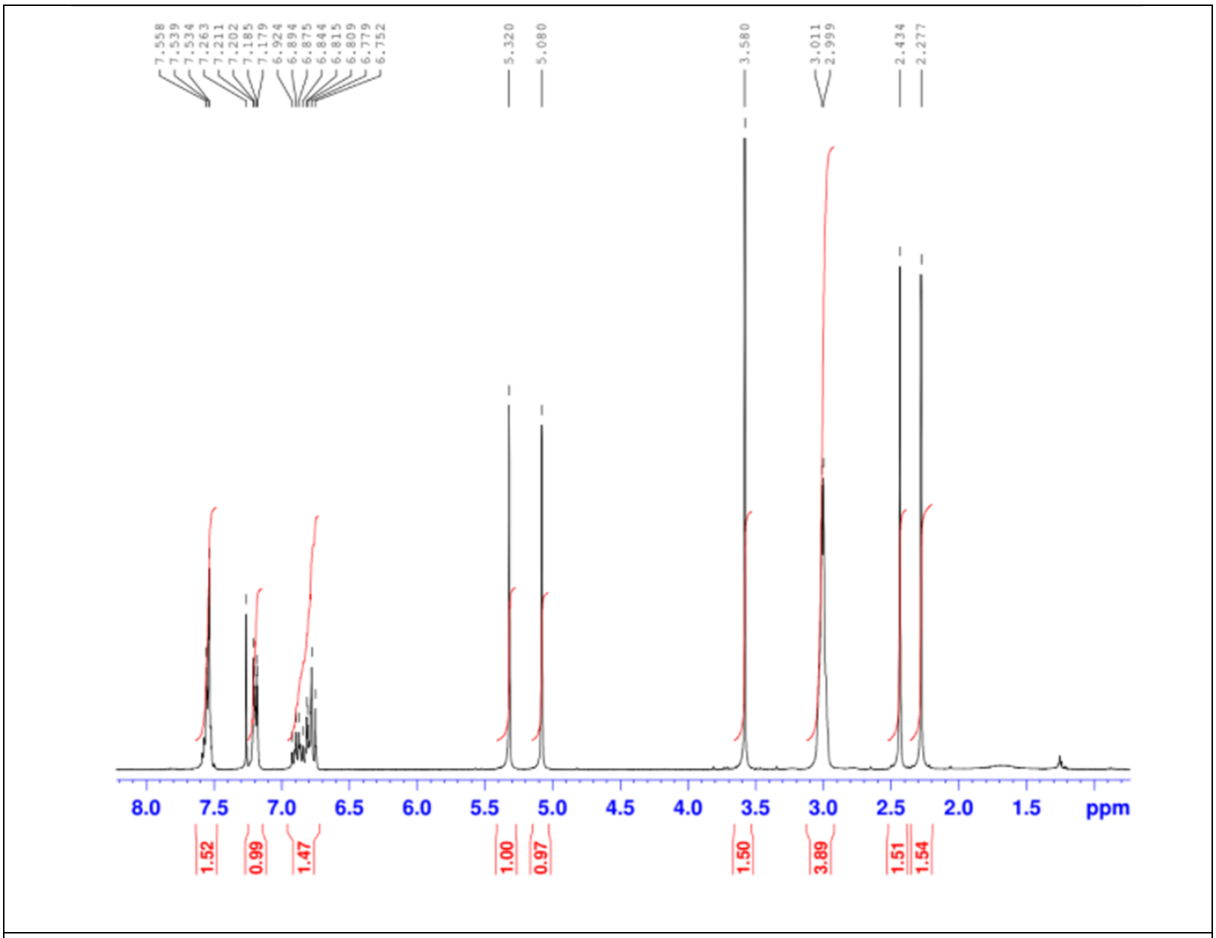
7.558 7.539 7.534 7.263 7.211 7.202 7.185 7.179 6.924 6.894 6.875 6.844 6.815 6.809 6.779 6.752
5.320
5.080
3.580
3.011 2.999
2.434
2.277
8.0 7.5 7.0 6.5 6.0 5.5 5.0 4.5 4.0 3.5 3.0 2.5 2.0 1.5 ppm
1.52 0.99 1.47 1.00 0.97 1.50 3.89 1.51 1.54

3a

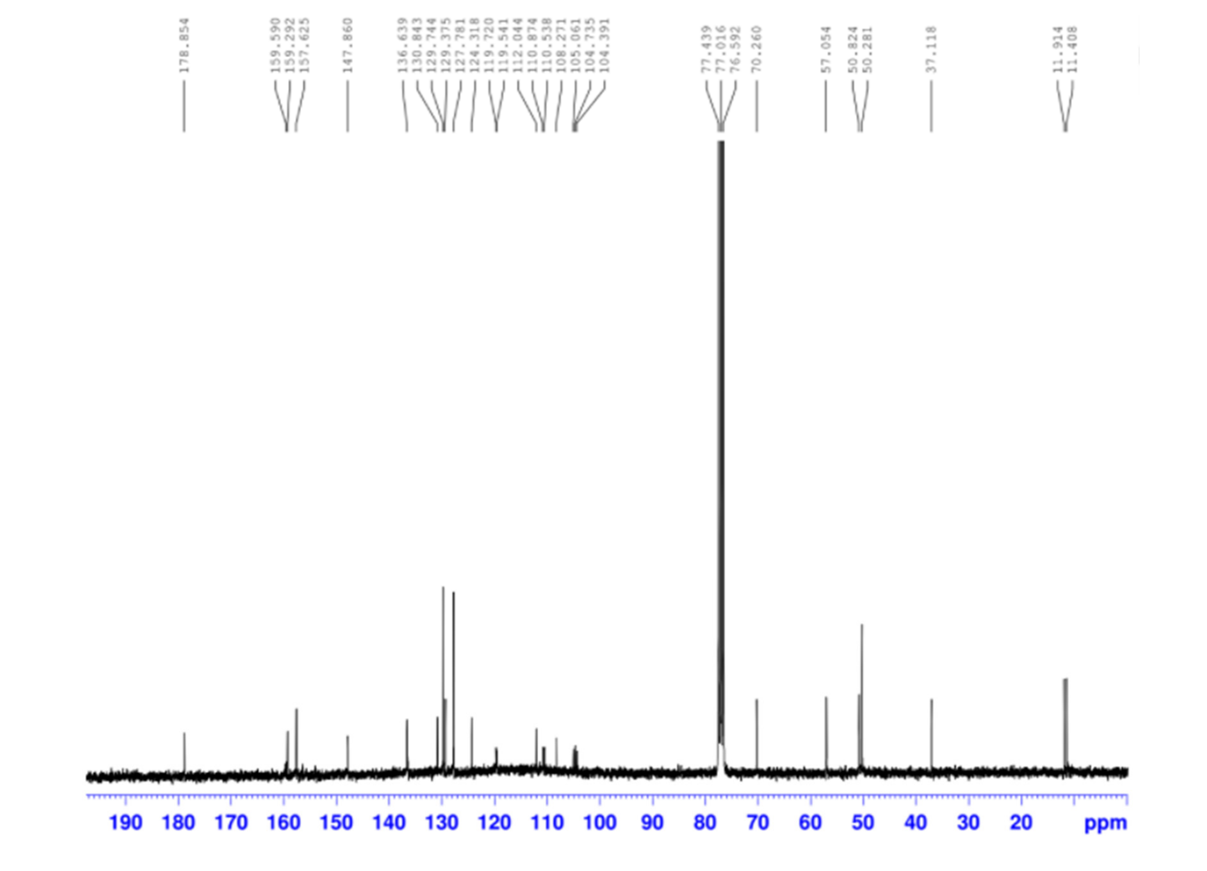
178.854
159.590 159.292 157.625
147.860
136.639 130.843 129.744 129.375 127.781 124.318 119.720 119.541 112.044 110.874 110.538 108.271 105.061 104.735 104.391
77.439 77.016 76.592
70.260
57.054
50.824 50.281
37.118
11.914 11.408
190 180 170 160 150 140 130 120 110 100 90 80 70 60 50 40 30 20 ppm

3a

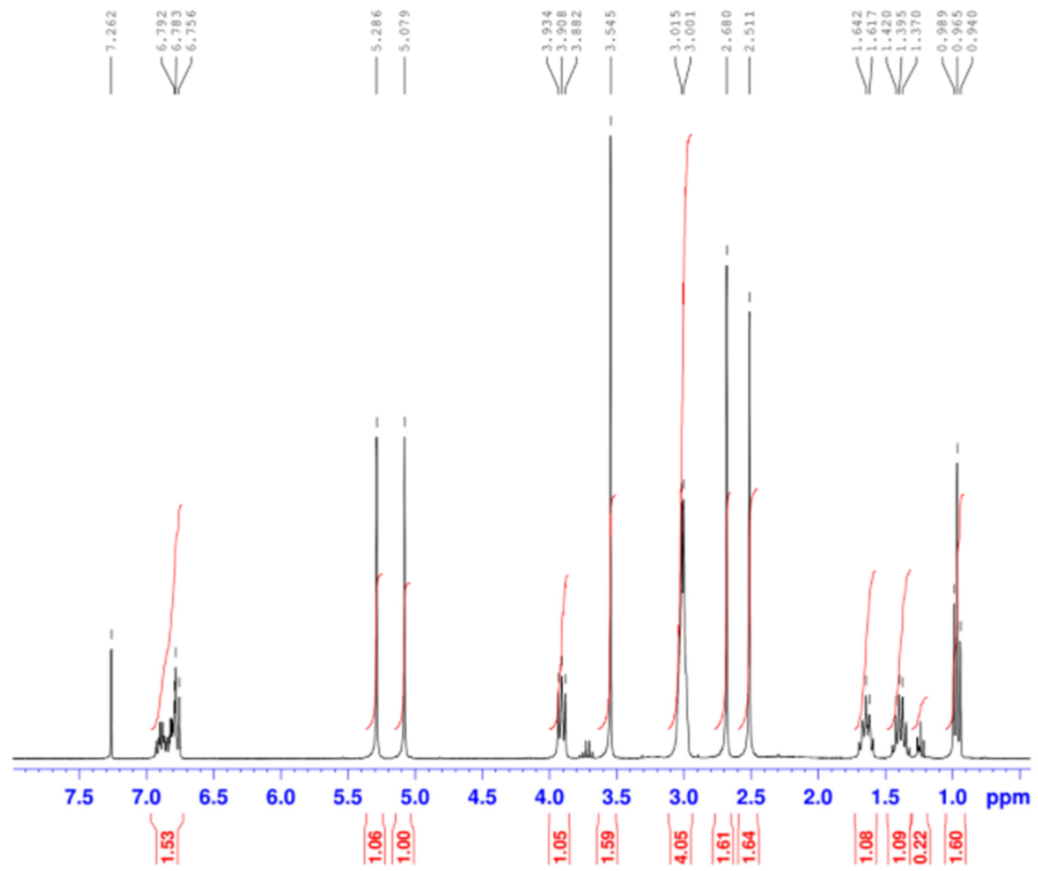
7.262
6.792
6.783
6.756
5.286
5.079
3.934
3.908
3.882
3.545
3.015
3.001
2.680
2.511
1.642
1.617
1.420
1.395
1.370
0.989
0.965
0.940
7.5 7.0 6.5 6.0 5.5 5.0 4.5 4.0 3.5 3.0 2.5 2.0 1.5 1.0 ppm
1.53
1.06
1.00
1.05
1.59
4.05
1.61
1.64
1.08
1.09
0.22
1.60

3b

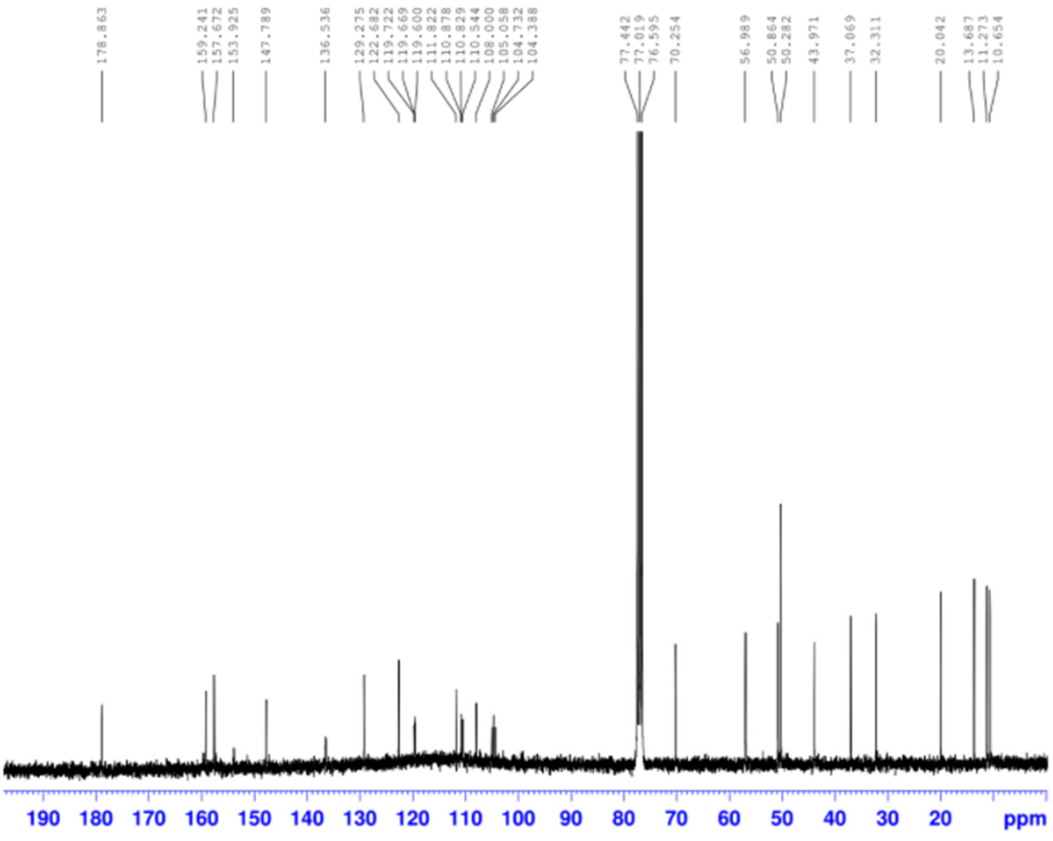
178.863
159.241
157.672
153.925
147.789
136.536
129.275
122.682
119.722
119.669
119.600
111.822
110.878
110.829
110.544
108.000
105.058
104.732
104.388
77.442
77.019
76.595
70.254
56.989
50.864
50.282
43.971
37.069
32.311
20.042
13.687
11.273
10.654
190 180 170 160 150 140 130 120 110 100 90 80 70 60 50 40 30 20 ppm

3b

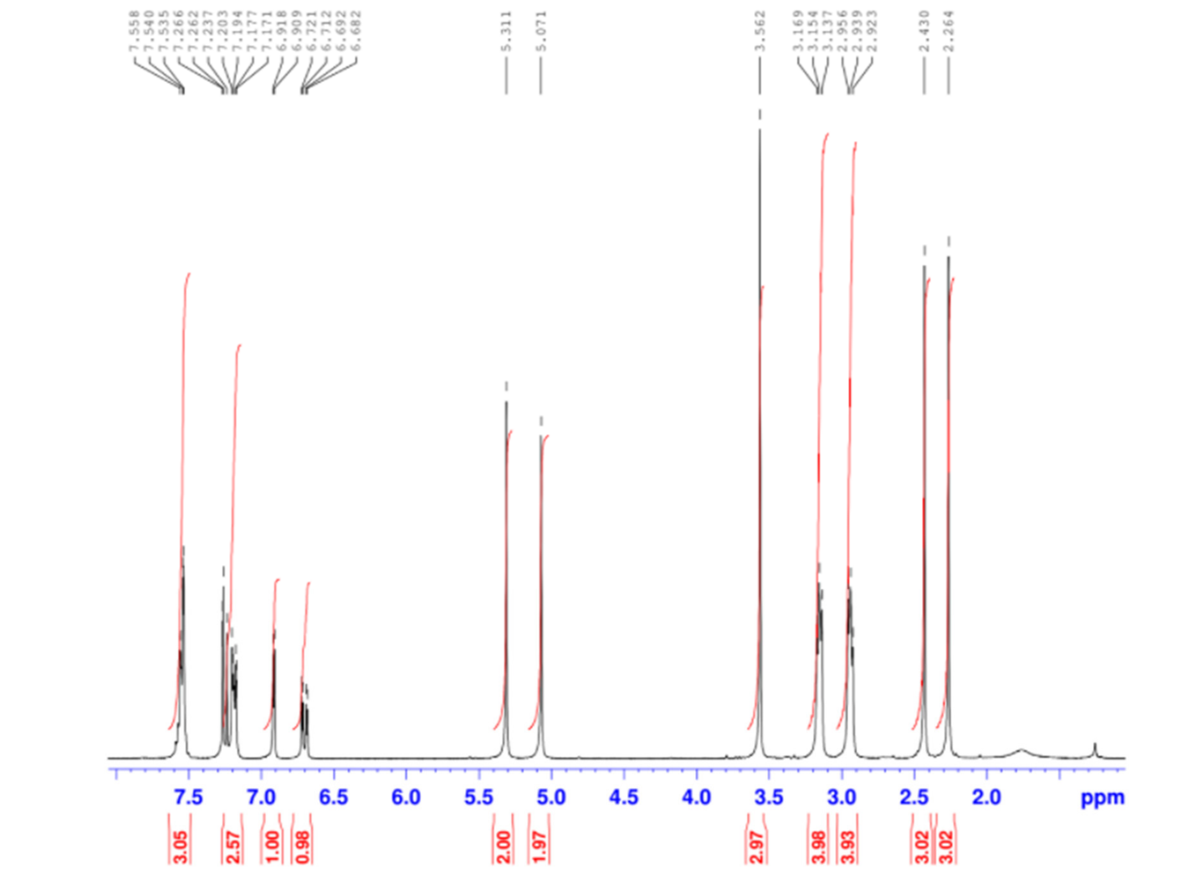

7.558 7.540 7.535 7.266 7.262 7.237 7.203 7.194 7.177 7.171 6.918 6.909 6.721 6.712 6.692 6.682
5.311 5.071
3.562 3.169 3.154 3.137 2.956 2.939 2.923 2.430 2.264
7.5 7.0 6.5 6.0 5.5 5.0 4.5 4.0 3.5 3.0 2.5 2.0 ppm
3.05 2.57 1.00 0.98 2.00 1.97 2.97 3.98 3.93 3.02 3.02

4a

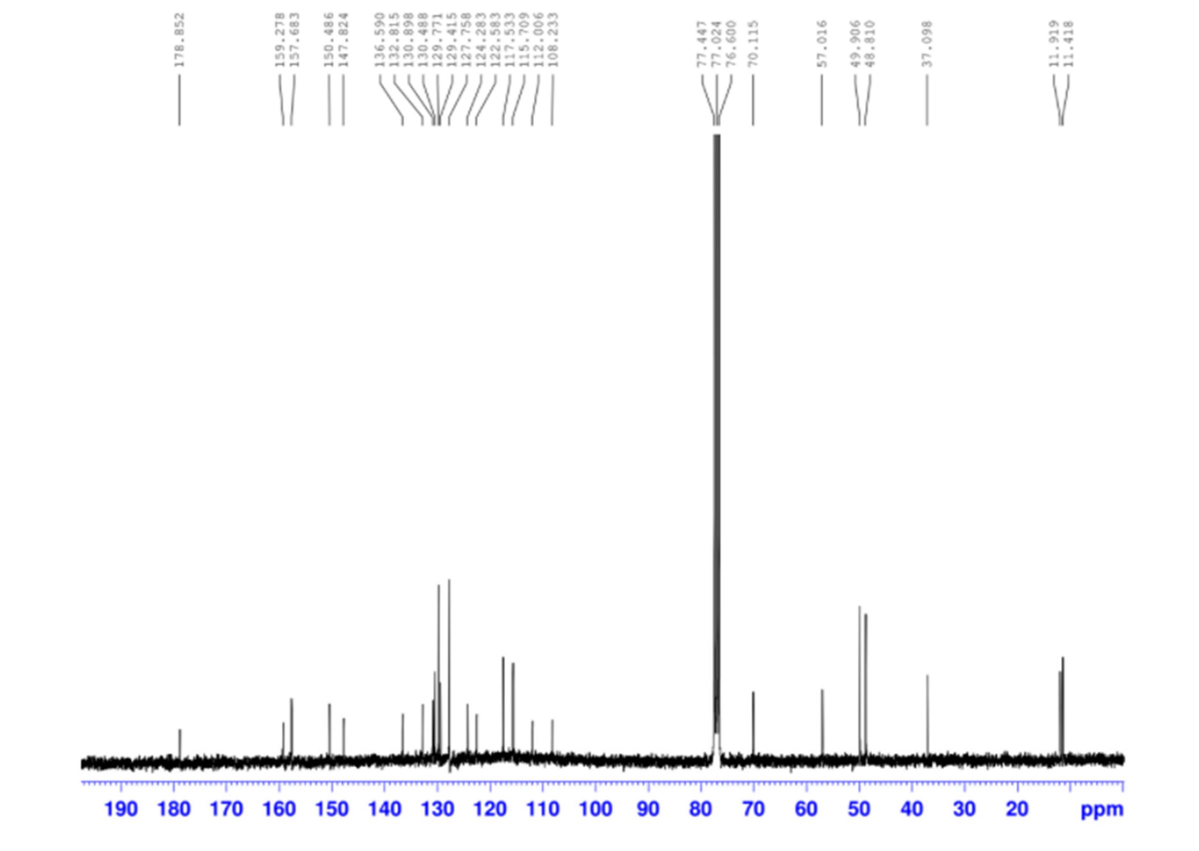

178.852
159.278 157.683
150.486 147.824
136.590 132.815 130.898 130.488 129.771 129.415 127.758 124.283 122.583 117.533 115.709 112.006 108.233
77.447 77.024 76.600 70.115
57.016 49.906 48.810 37.098
11.919 11.418
190 180 170 160 150 140 130 120 110 100 90 80 70 60 50 40 30 20 ppm

4a

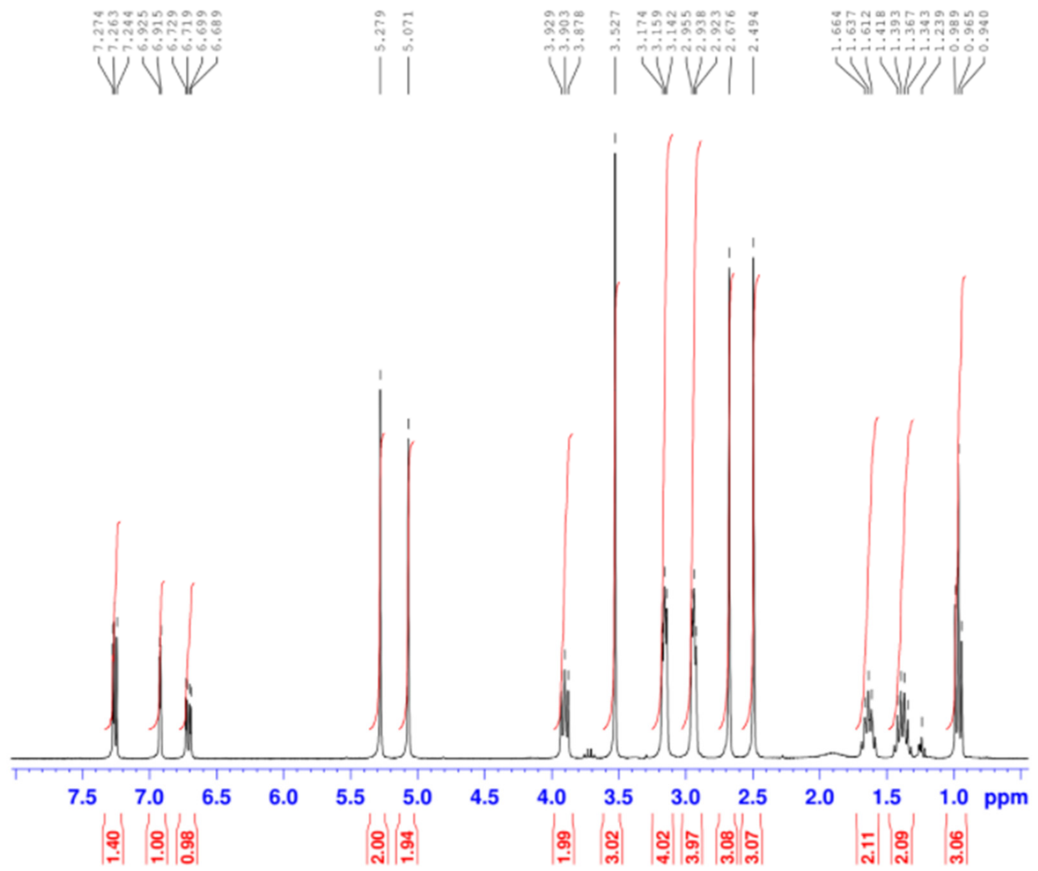
7.274
7.263
7.244
6.925
6.915
6.729
6.719
6.699
6.689
5.279
5.071
3.929
3.903
3.878
3.527
3.174
3.159
3.142
2.955
2.938
2.923
2.676
2.494
1.664
1.637
1.612
1.418
1.393
1.367
1.343
1.239
0.989
0.965
0.940
7.5
7.0
6.5
6.0
5.5
5.0
4.5
4.0
3.5
3.0
2.5
2.0
1.5
1.0
ppm
1.40
1.00
0.98
2.00
1.94
1.99
3.02
4.02
3.97
3.08
3.07
2.11
2.09
3.06

4b

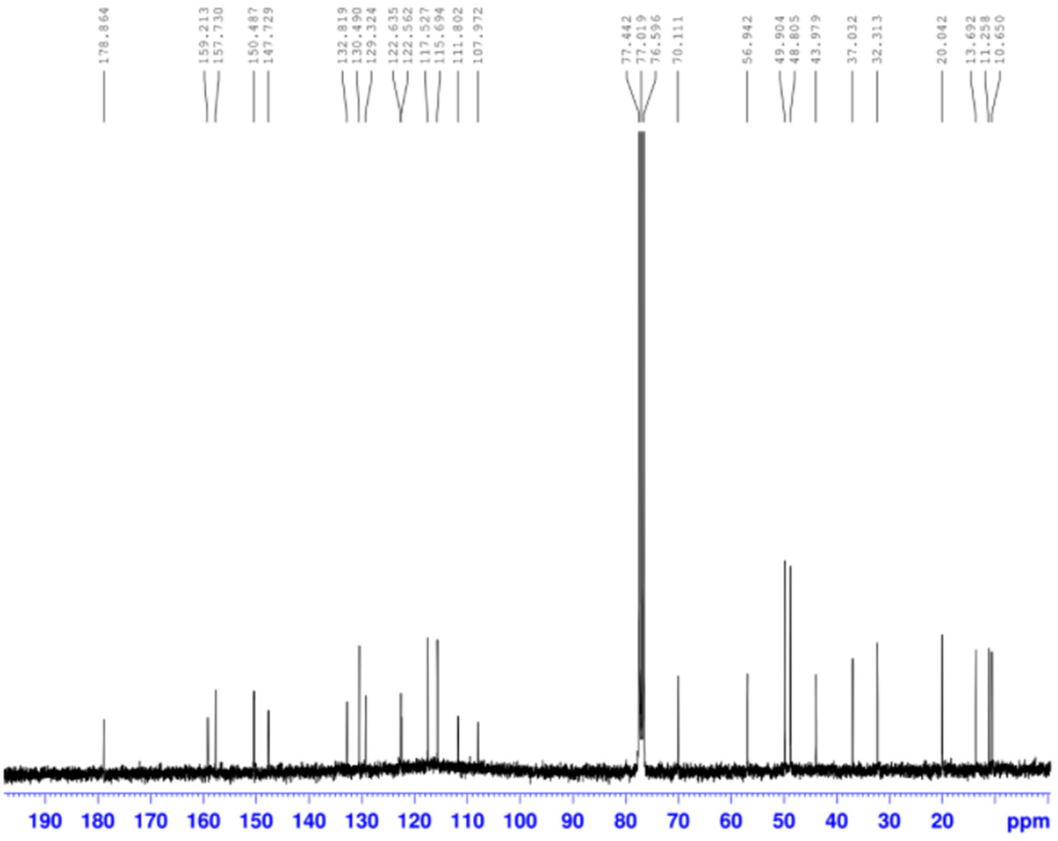
178.864
159.213
157.730
150.487
147.729
132.819
130.490
129.324
122.635
122.562
117.527
115.694
111.802
107.972
77.442
77.019
76.596
70.111
56.942
49.904
48.805
43.979
37.032
32.313
20.042
13.692
11.258
10.650
190
180
170
160
150
140
130
120
110
100
90
80
70
60
50
40
30
20
ppm

4b

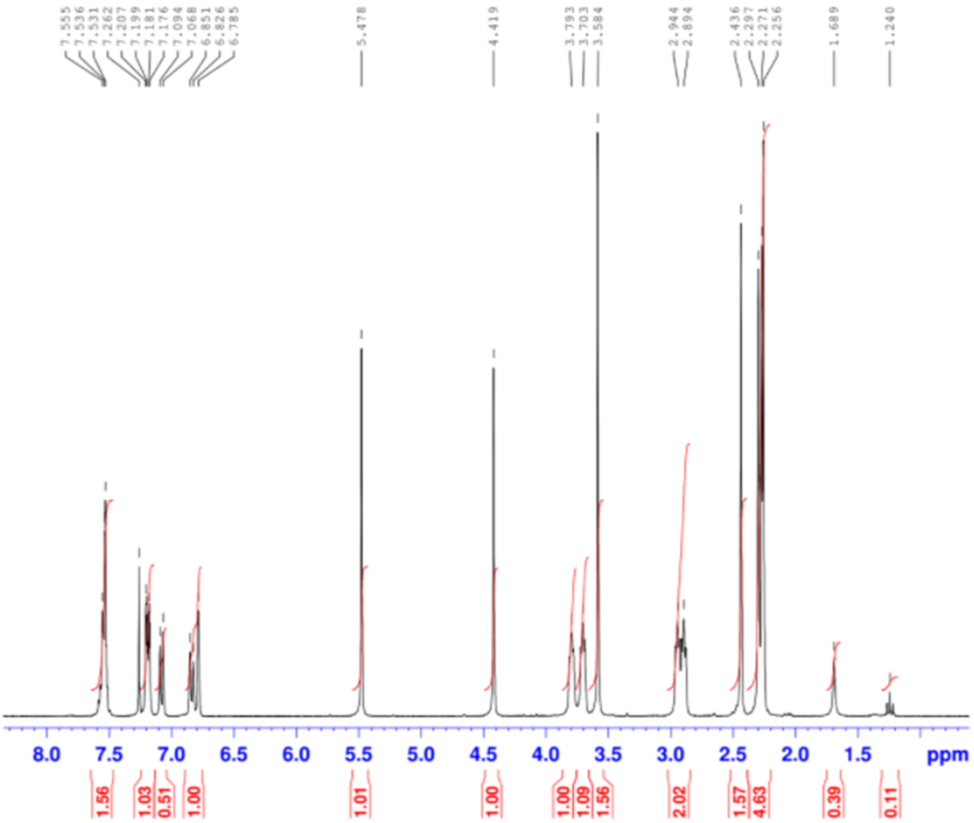
7.555
7.536
7.531
7.262
7.207
7.199
7.181
7.176
7.094
7.068
6.851
6.826
6.785
5.478
4.419
3.793
3.703
3.584
2.944
2.894
2.436
2.297
2.271
2.256
1.689
1.240
8.0
7.5
7.0
6.5
6.0
5.5
5.0
4.5
4.0
3.5
3.0
2.5
2.0
1.5
ppm
1.56
1.03
0.51
1.00
1.01
1.00
1.00
1.09
1.56
2.02
1.57
4.63
0.39
0.11

5a

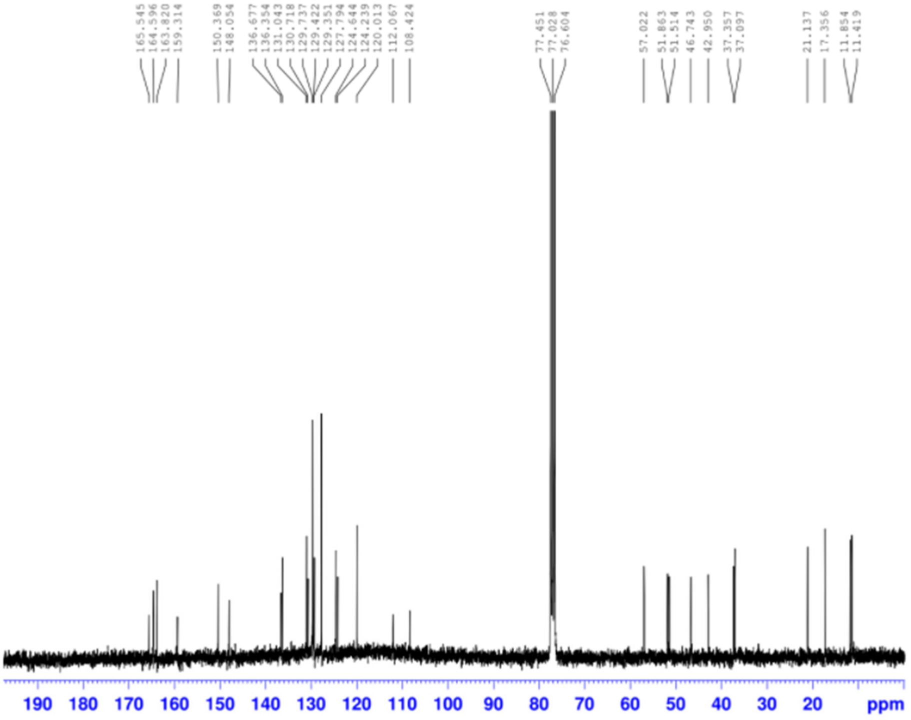
165.545
164.596
163.820
159.314
150.369
148.054
136.677
136.354
131.043
130.718
129.737
129.422
129.351
127.794
124.644
124.239
120.013
112.067
108.424
77.451
77.028
76.604
57.022
51.863
51.514
46.743
42.950
37.357
37.097
21.137
17.356
11.854
11.419
190
180
170
160
150
140
130
120
110
100
90
80
70
60
50
40
30
20
ppm

5a

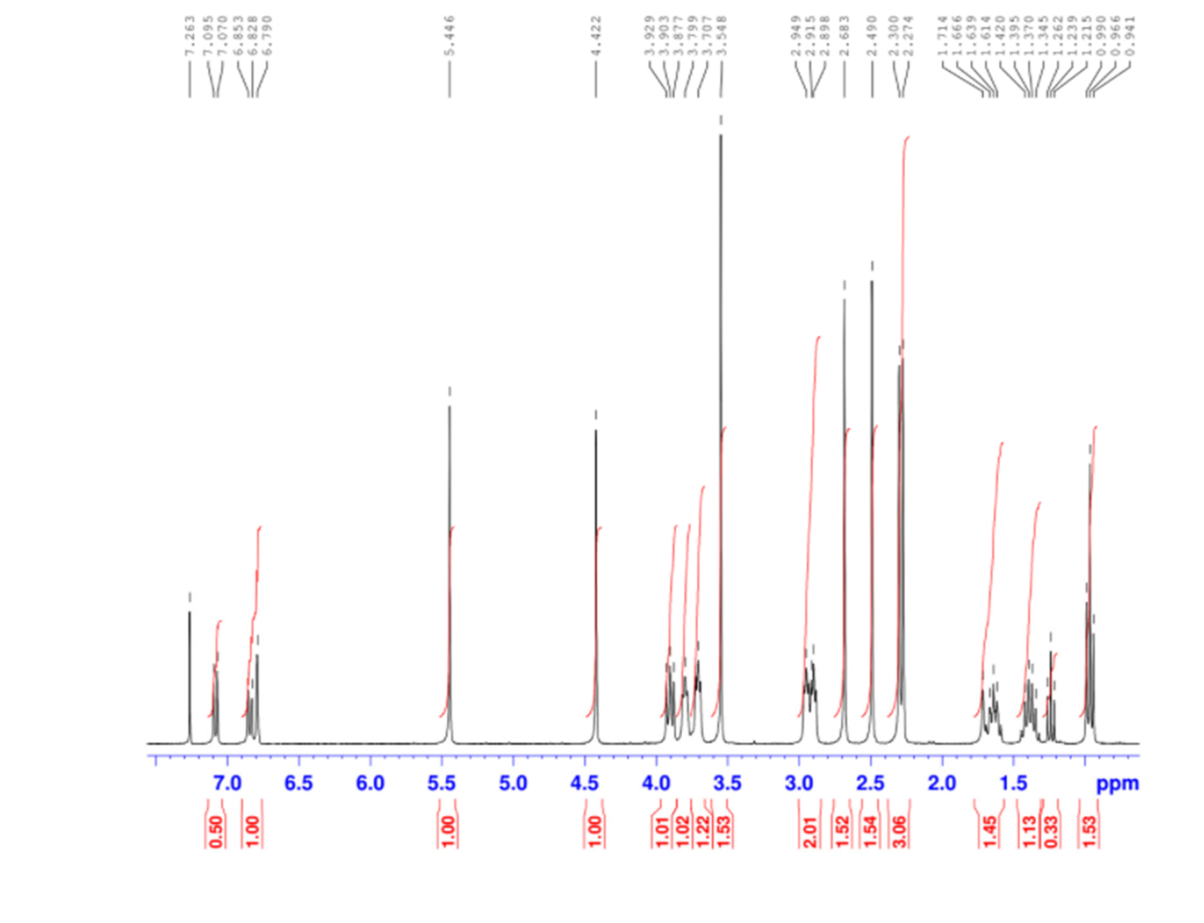
7.263
7.095
7.070
6.853
6.828
6.790
5.446
4.422
3.929
3.903
3.877
3.799
3.707
3.548
2.949
2.915
2.898
2.683
2.490
2.300
2.274
1.714
1.666
1.639
1.614
1.420
1.395
1.370
1.345
1.262
1.239
1.215
0.990
0.966
0.941
7.0 6.5 6.0 5.5 5.0 4.5 4.0 3.5 3.0 2.5 2.0 1.5 ppm
0.50
1.00
1.00
1.00
1.01
1.02
1.22
1.53
2.01
1.52
1.54
3.06
1.45
1.13
0.33
1.53

5b

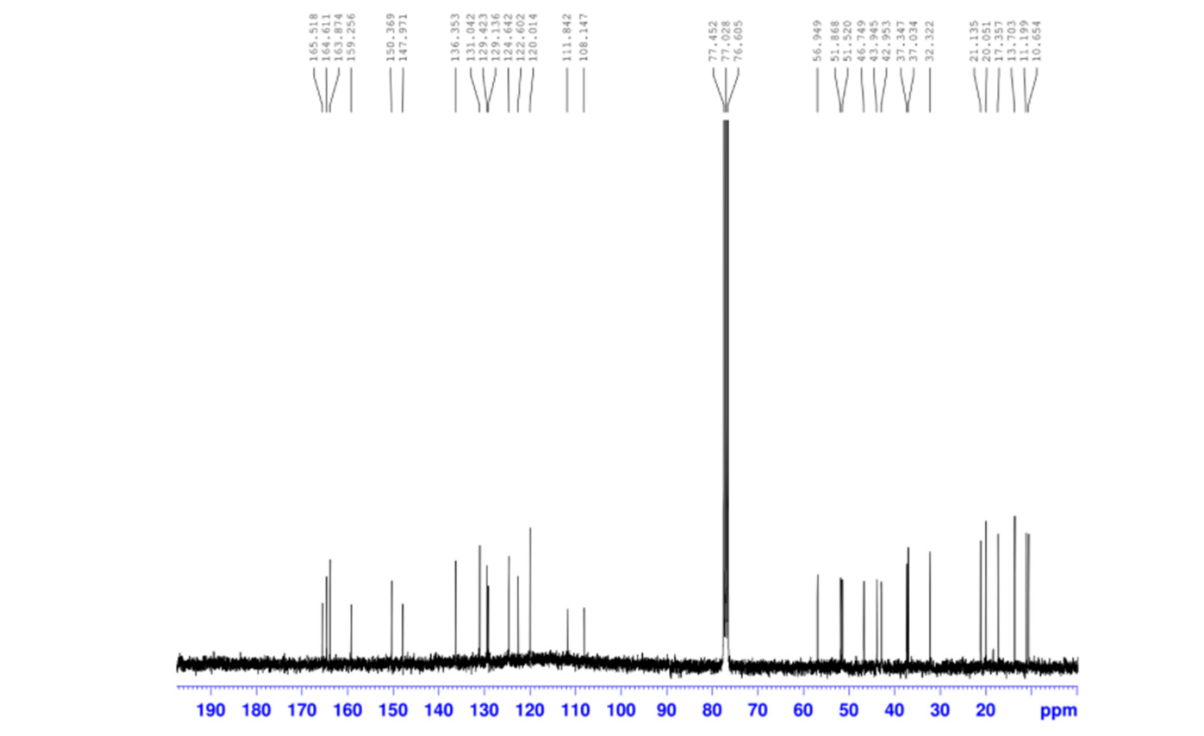
165.518
164.611
163.874
159.256
150.369
147.971
136.353
131.042
129.423
129.136
124.642
122.602
120.014
111.842
108.147
77.452
77.028
76.605
56.949
51.868
51.520
46.749
43.945
42.953
37.347
37.034
32.322
21.135
20.051
17.357
13.703
11.199
10.654
190 180 170 160 150 140 130 120 110 100 90 80 70 60 50 40 30 20 ppm

5b

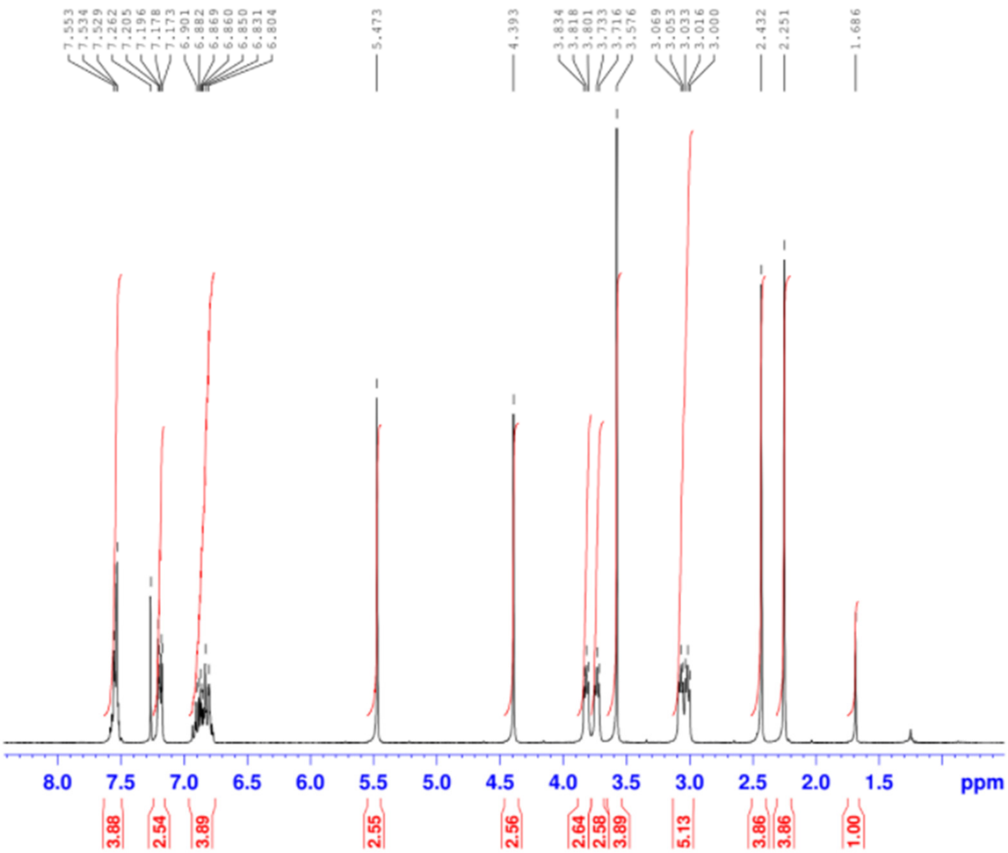

6a

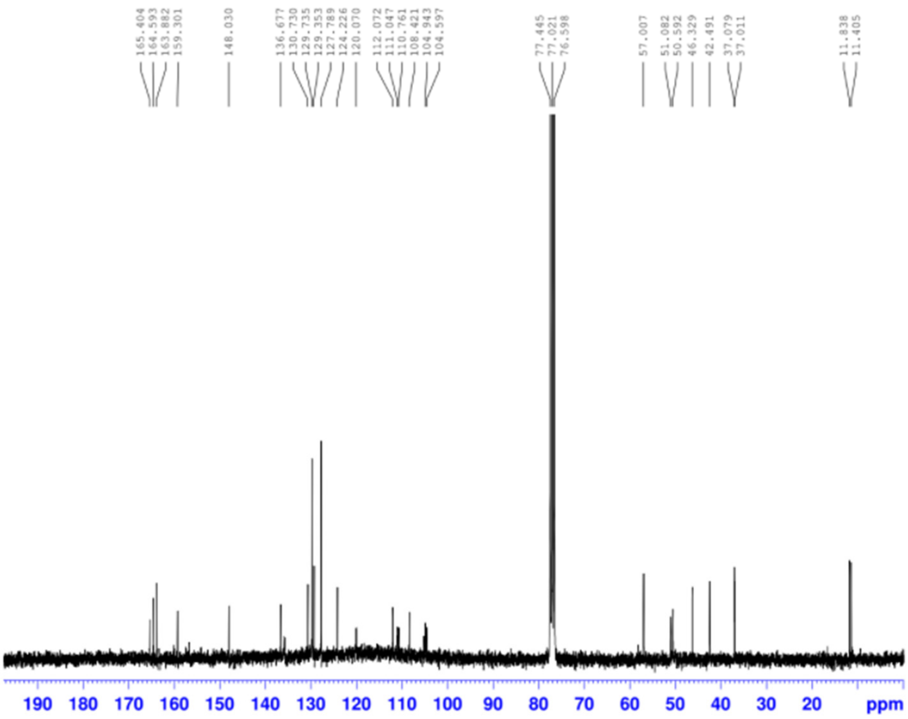

6a

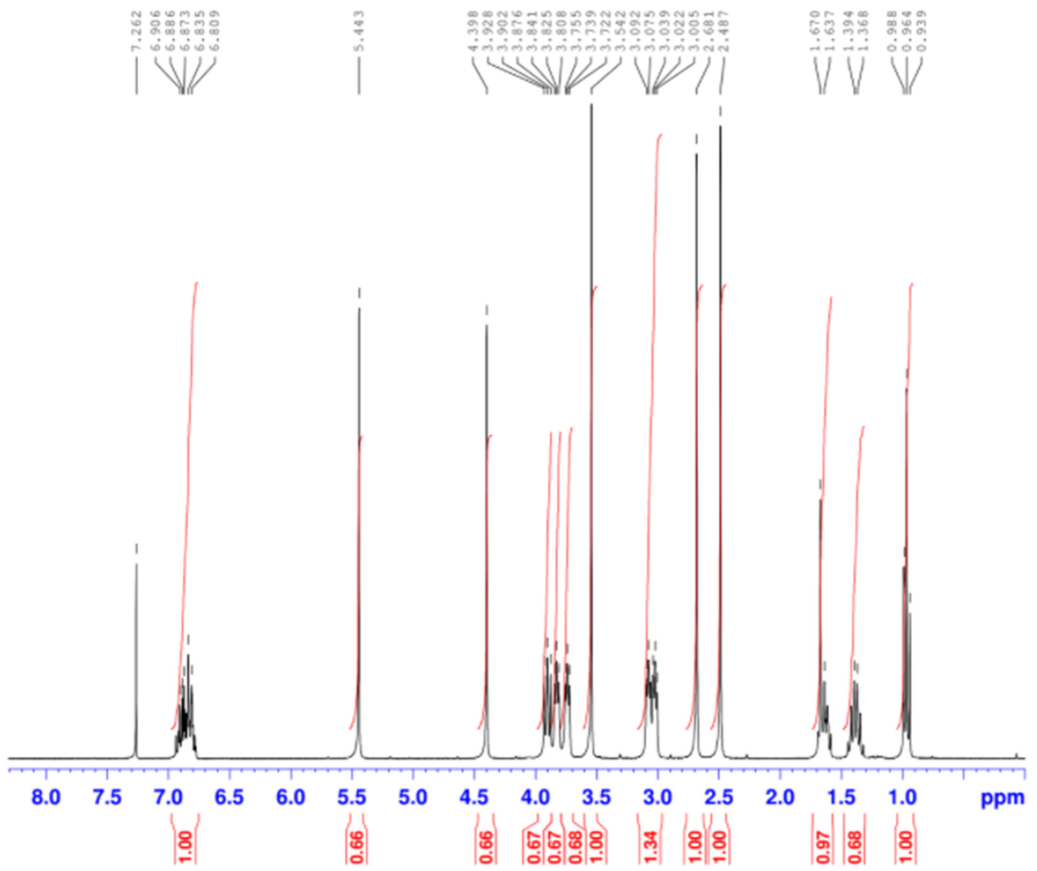

6b

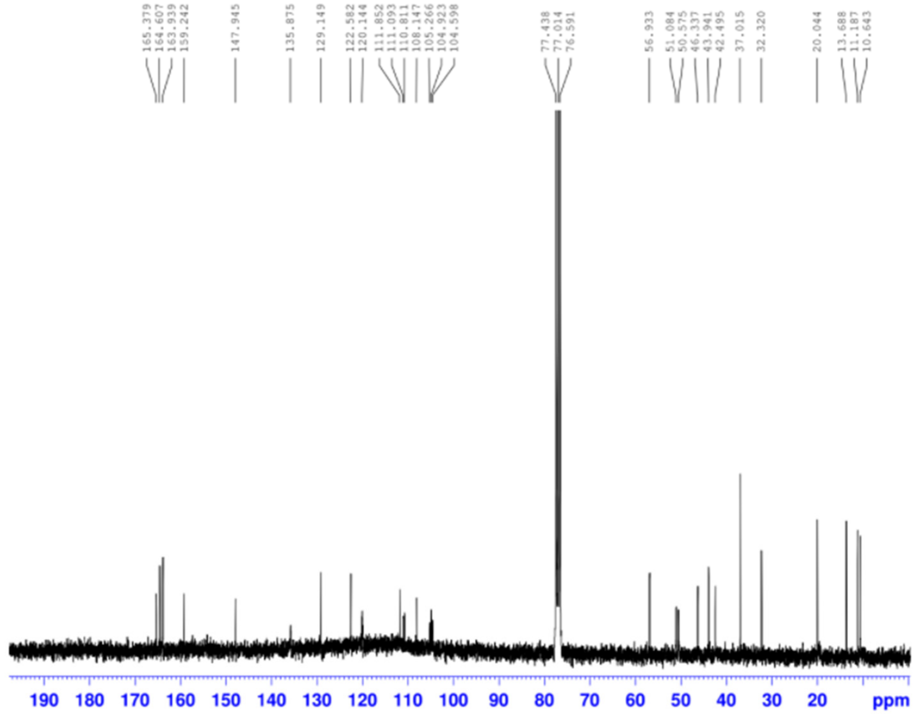

6b

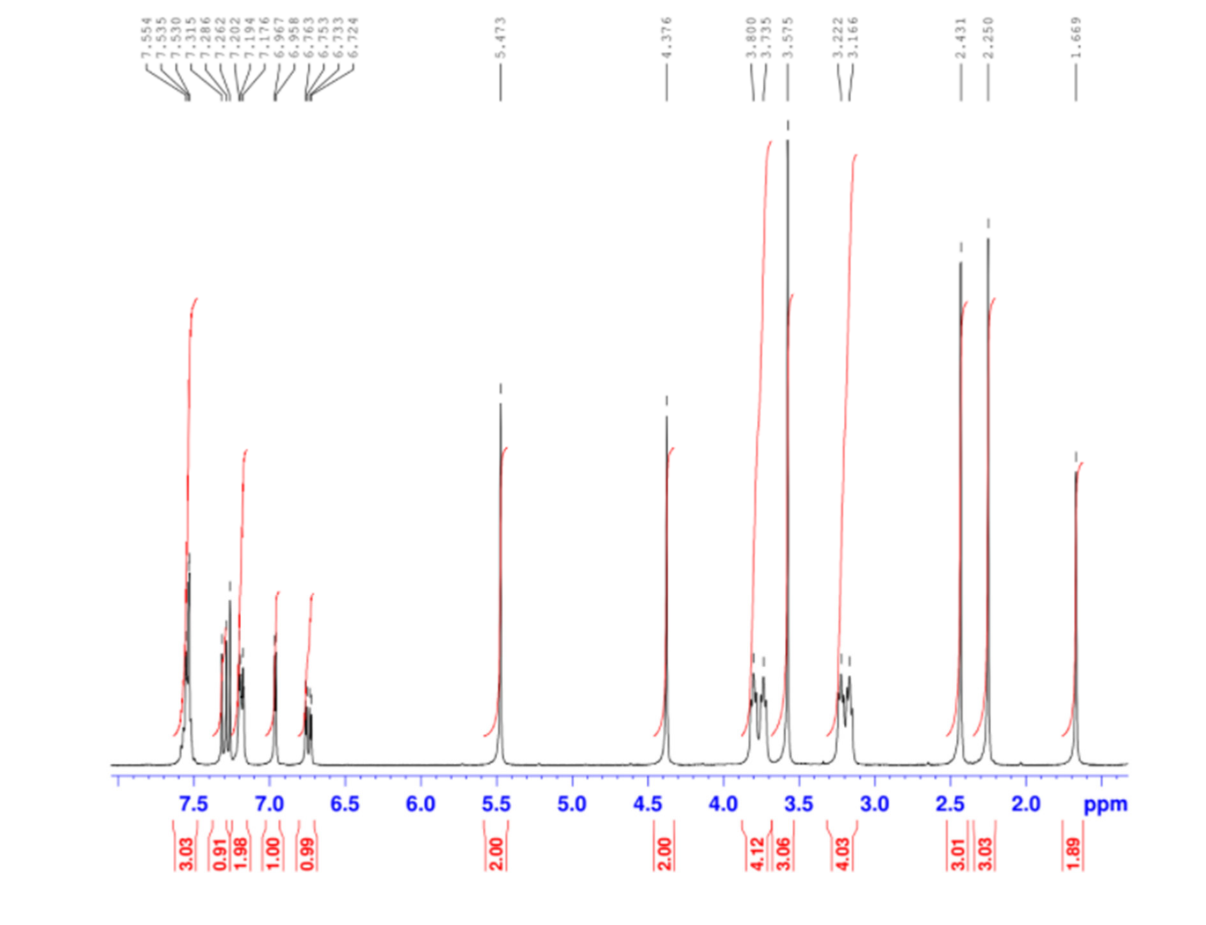
7.554
7.535
7.530
7.315
7.286
7.262
7.202
7.194
7.176
6.967
6.958
6.763
6.753
6.733
6.724
5.473
4.376
3.800
3.735
3.575
3.222
3.166
2.431
2.250
1.669
7.5 7.0 6.5 6.0 5.5 5.0 4.5 4.0 3.5 3.0 2.5 2.0 ppm
3.03
0.91
1.98
1.00
0.99
2.00
2.00
4.12
3.06
4.03
3.01
3.03
1.89

7a

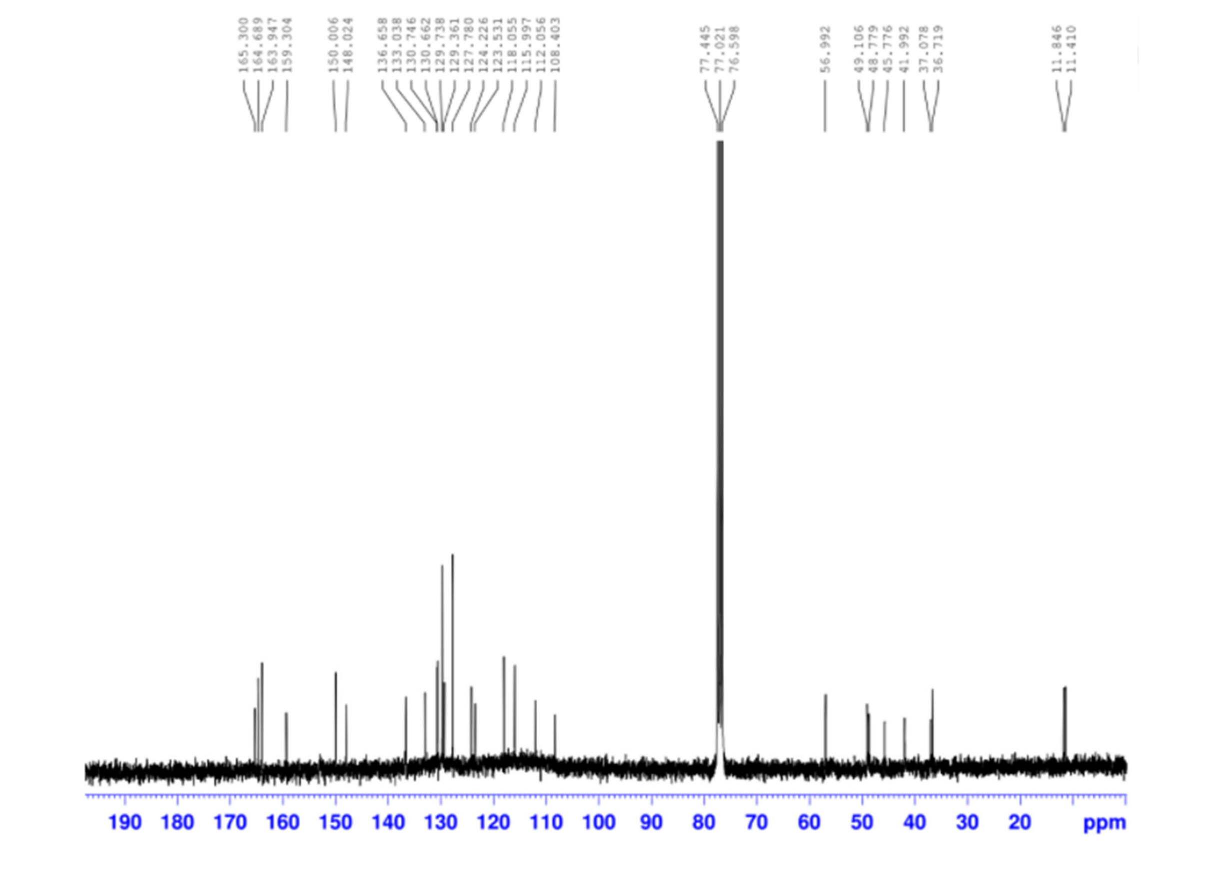
165.300
164.689
163.947
159.304
150.006
148.024
136.658
133.038
130.746
130.662
129.738
129.361
127.780
124.226
123.531
118.055
115.997
112.056
108.403
77.445
77.021
76.598
56.992
49.106
48.779
45.776
41.992
37.078
36.719
11.846
11.410
190 180 170 160 150 140 130 120 110 100 90 80 70 60 50 40 30 20 ppm

7a

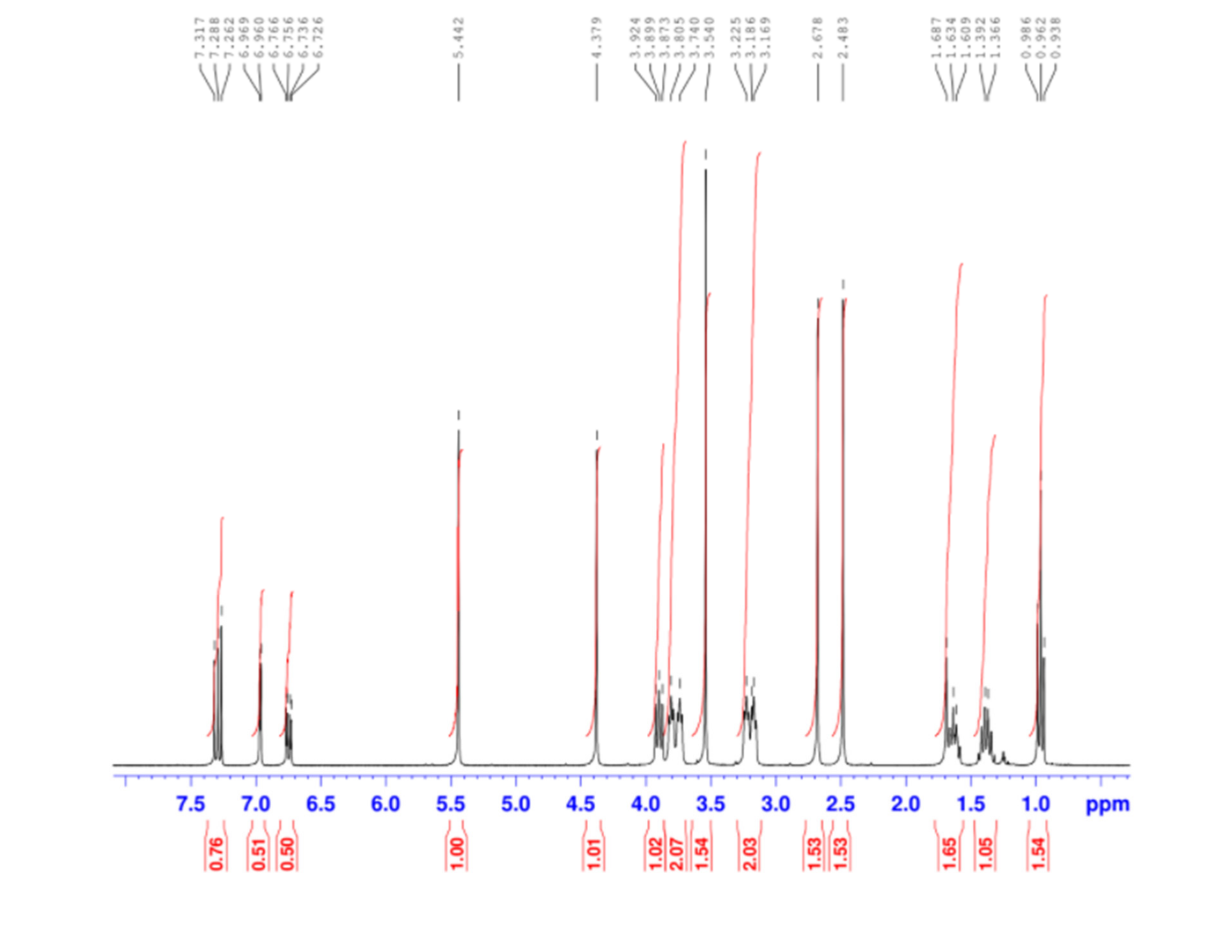
7.317
7.288
7.262
6.969
6.960
6.766
6.756
6.736
6.726
5.442
4.379
3.924
3.899
3.873
3.805
3.740
3.540
3.225
3.186
3.169
2.678
2.483
1.687
1.634
1.609
1.392
1.366
0.986
0.962
0.938
7.5
7.0
6.5
6.0
5.5
5.0
4.5
4.0
3.5
3.0
2.5
2.0
1.5
1.0
ppm
0.76
0.51
0.50
1.00
1.01
1.02
2.07
1.54
2.03
1.53
1.53
1.65
1.05
1.54

7b

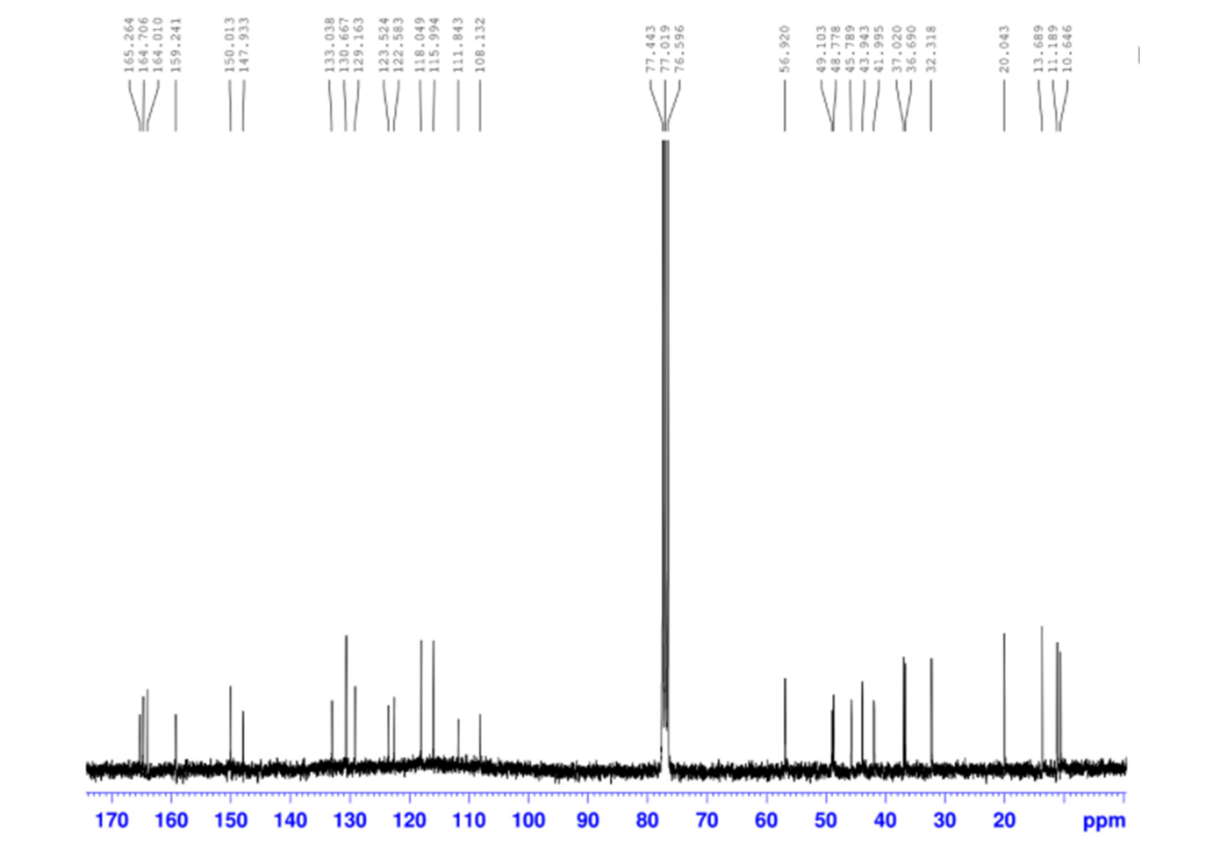
165.264
164.706
164.010
159.241
150.013
147.933
133.038
130.667
129.163
123.524
122.583
118.049
115.994
111.843
108.132
77.443
77.019
76.596
56.920
49.103
48.778
45.789
43.943
41.995
37.020
36.690
32.318
20.043
13.689
11.189
10.646
170
160
150
140
130
120
110
100
90
80
70
60
50
40
30
20
ppm

7b

Table S3. IR spectra of new compounds

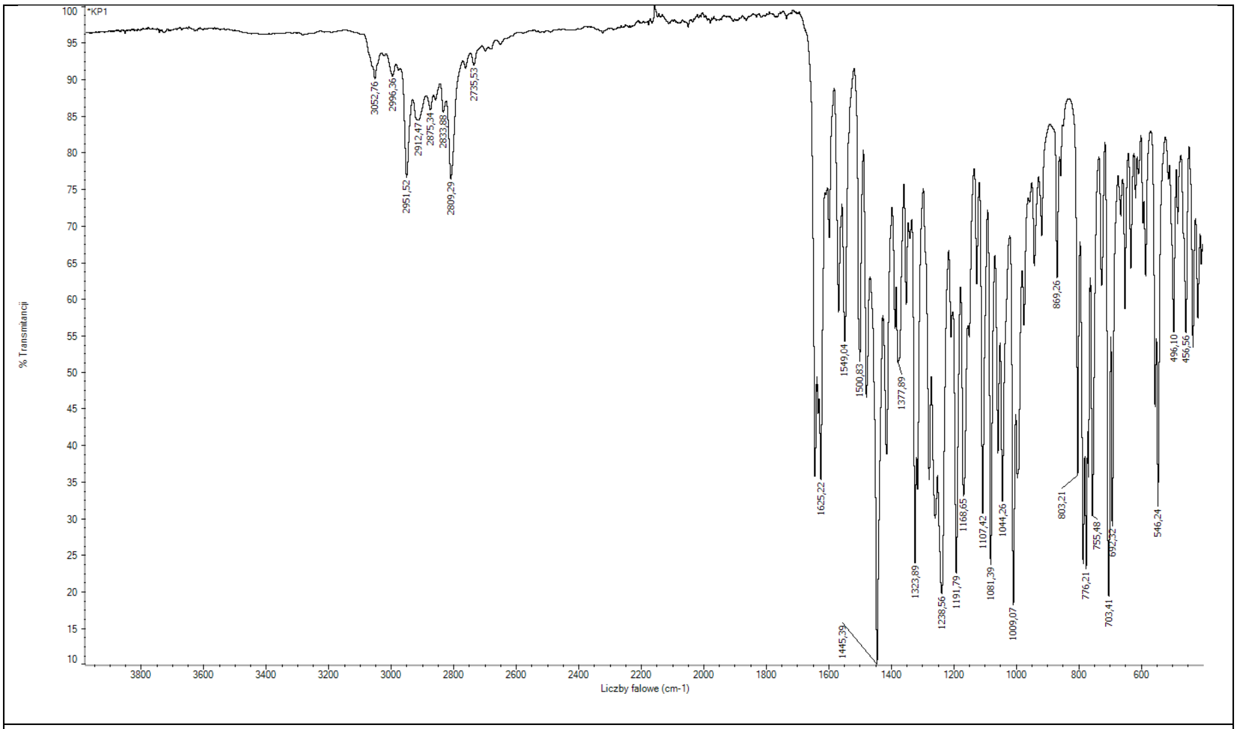

2a

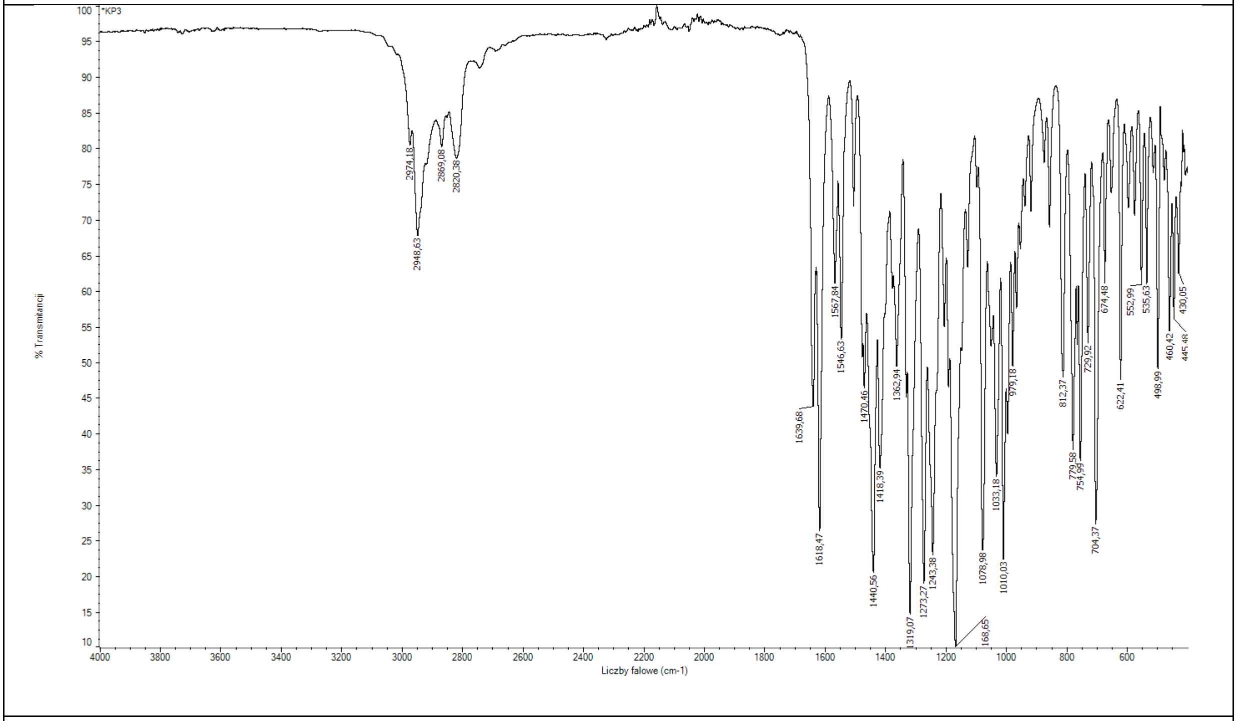

2b

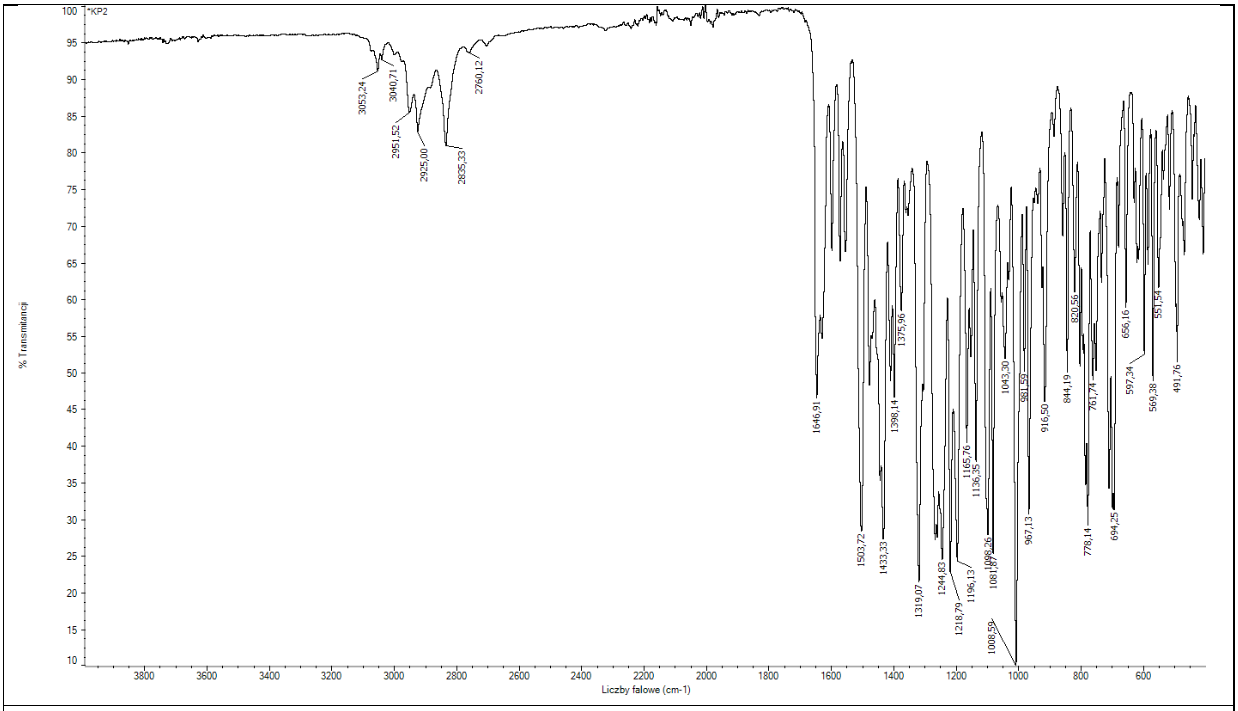
*KP2
% Transmitancji
Liczby falowe (cm-1)
3053,24
3040,71
2951,52
2925,00
2835,33
2760,12
1646,91
1503,72
1433,33
1398,14
1375,96
1319,07
1244,83
1218,79
1196,13
1165,76
1136,35
1098,26
1081,87
1043,30
1008,59
981,59
967,13
916,50
844,19
820,56
778,14
761,74
694,25
656,16
597,34
569,38
551,54
491,76

3a

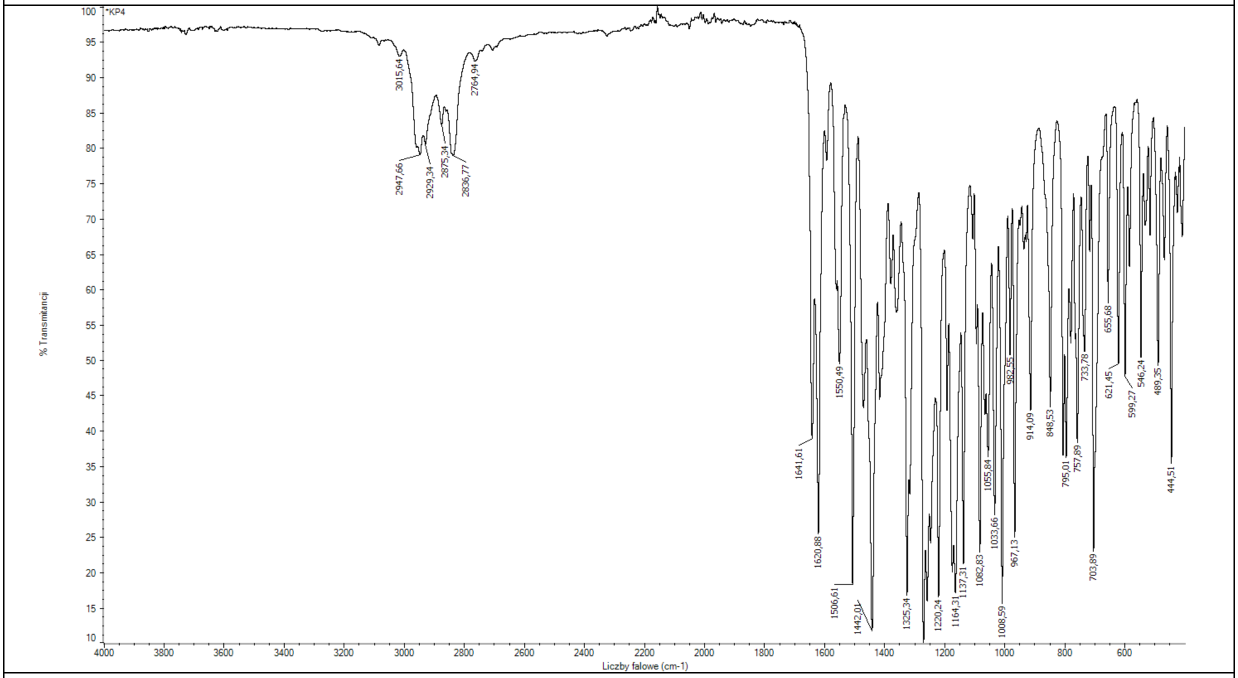
*KP4
% Transmitancji
Liczby falowe (cm-1)
3015,64
2947,66
2929,34
2875,34
2836,77
2764,94
1641,61
1620,88
1550,49
1506,61
1442,01
1325,34
1220,24
1164,31
1137,31
1082,83
1055,84
1033,66
1008,59
982,55
967,13
914,09
848,53
795,01
757,89
733,78
703,89
655,68
621,45
599,27
546,24
489,35
444,51

3b

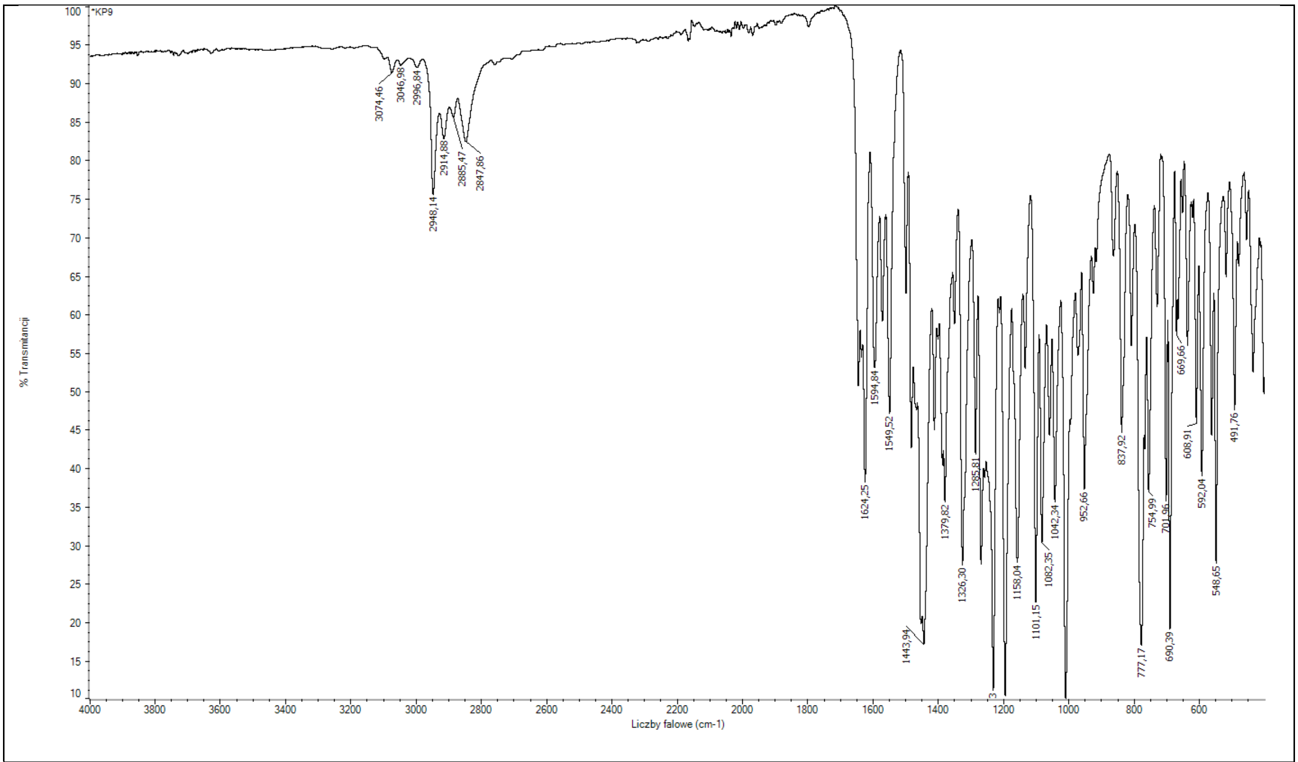
*KP9
% Transmitancji
Liczby falowe (cm-1)
3074,46
3046,98
2996,84
2948,14
2914,88
2885,47
2847,86
1624,25
1594,84
1549,52
1443,94
1379,82
1326,30
1285,81
1158,04
1101,15
1082,35
1042,34
952,66
837,92
777,17
754,99
701,96
690,39
669,66
608,91
592,04
548,65
491,76

4a

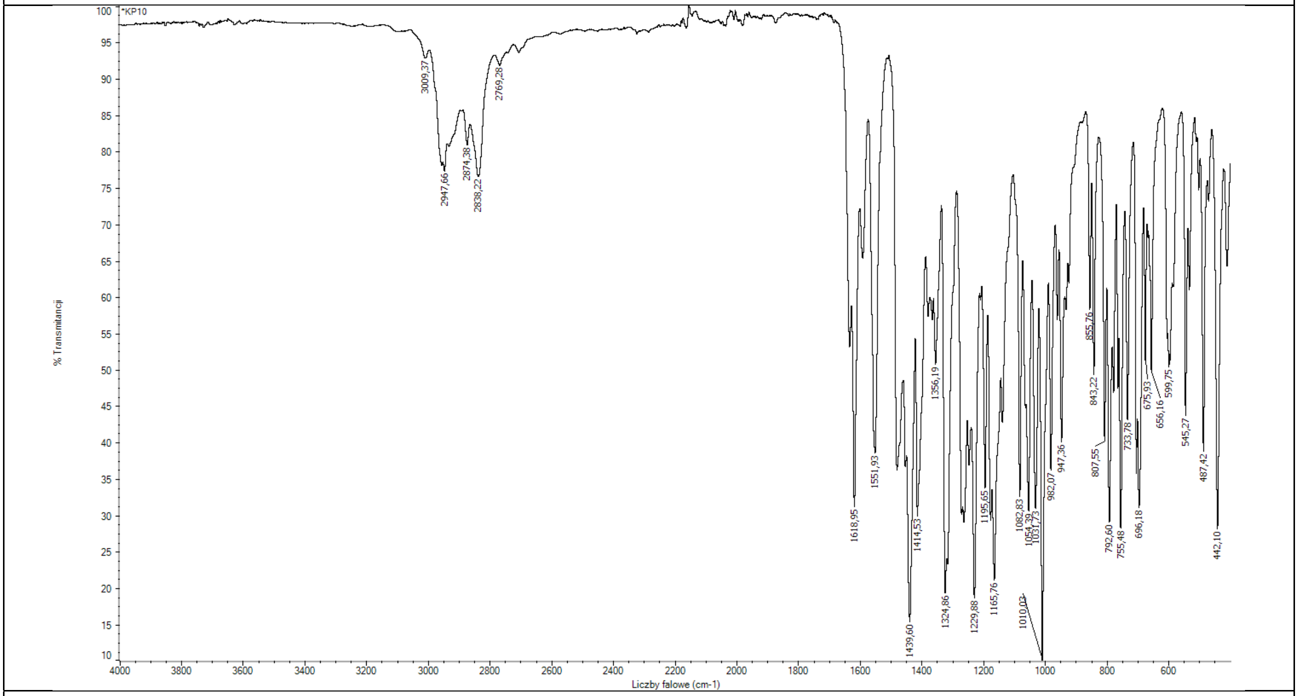
*KP10
% Transmitancji
Liczby falowe (cm-1)
3009,37
2947,66
2874,38
2838,22
2769,28
1618,95
1551,93
1439,60
1414,53
1356,19
1324,86
1229,88
1195,65
1165,76
1082,83
1054,39
1031,73
1010,03
982,07
947,36
855,76
843,22
807,55
792,60
755,48
733,78
696,18
675,93
656,16
599,75
545,27
487,42
442,10

4b

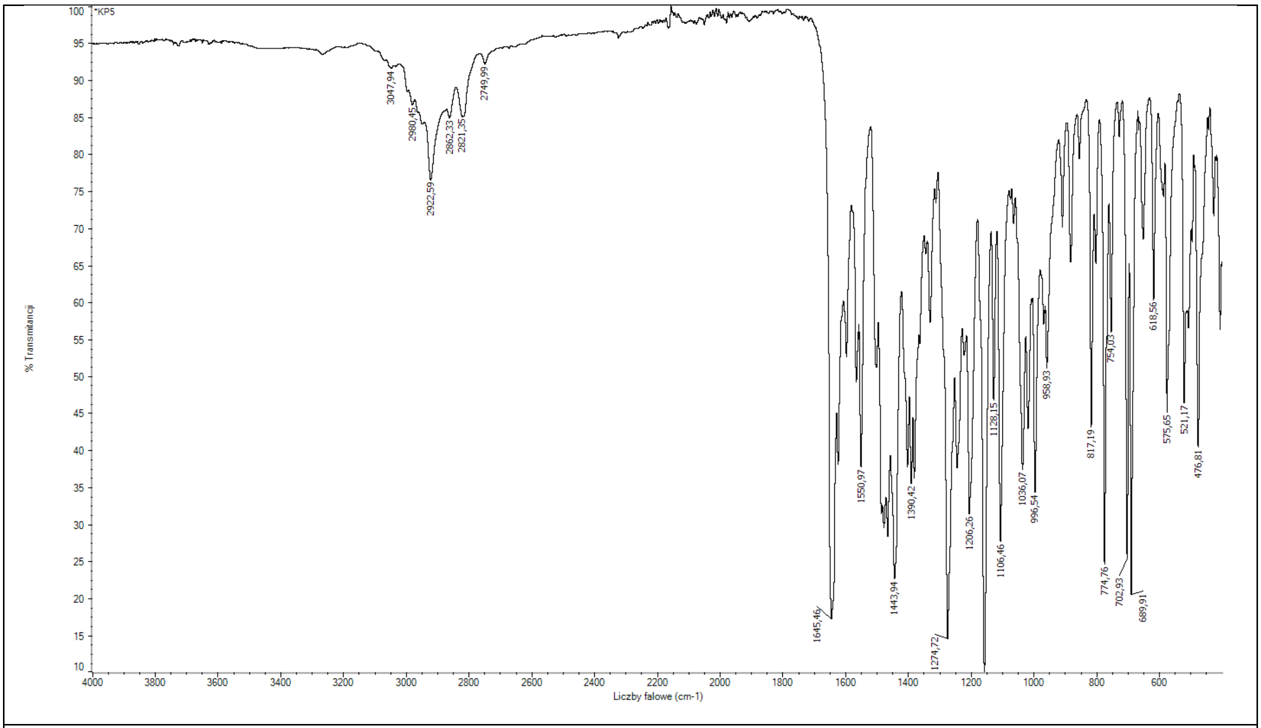
*KP5
% Transmitancji
Liczby falowe (cm-1)
3047,94
2980,45
2922,59
2862,33
2821,35
2749,99
1645,46
1550,97
1443,94
1390,42
1274,72
1206,26
1128,15
1106,46
1036,07
996,54
958,93
817,19
774,76
754,03
702,93
689,91
618,56
575,65
521,17
476,81

5a

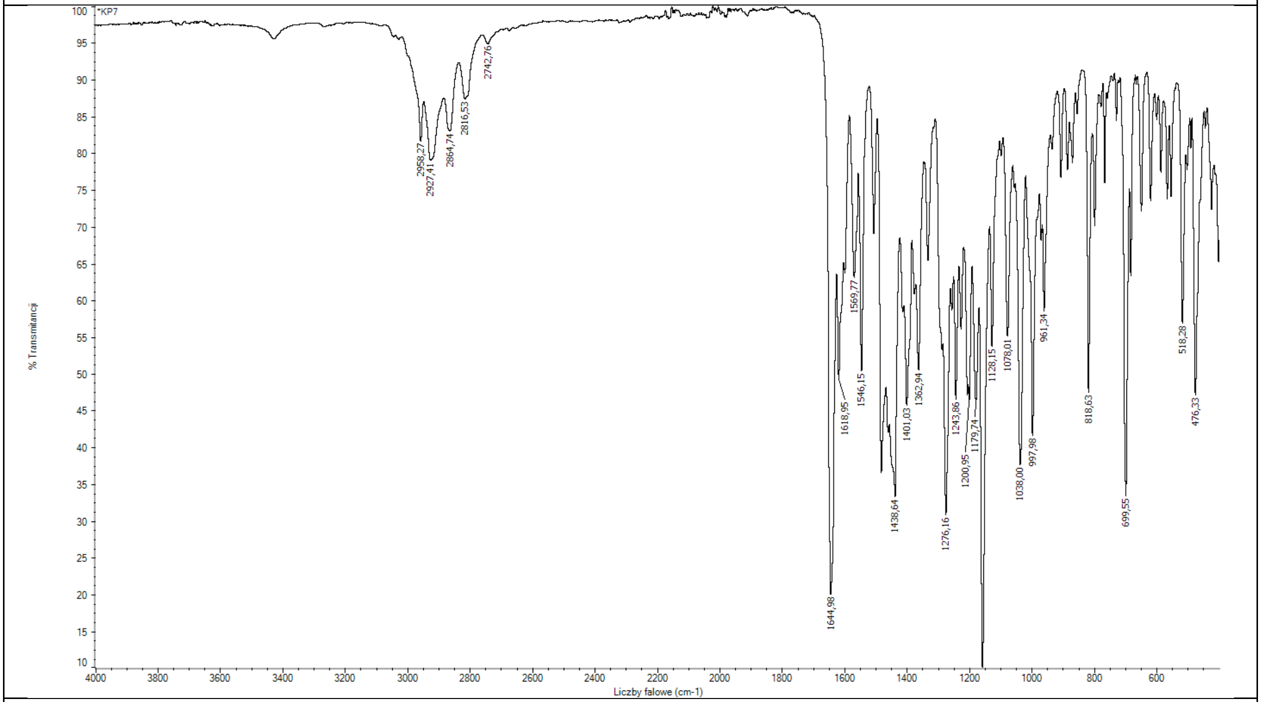
*KP7
% Transmitancji
Liczby falowe (cm-1)
2958,27
2927,41
2864,74
2816,53
2742,76
1644,98
1618,95
1569,77
1546,15
1438,64
1401,03
1362,94
1276,16
1243,86
1200,95
1179,74
1128,15
1078,01
1038,00
997,98
961,34
818,63
699,55
518,28
476,33

5b

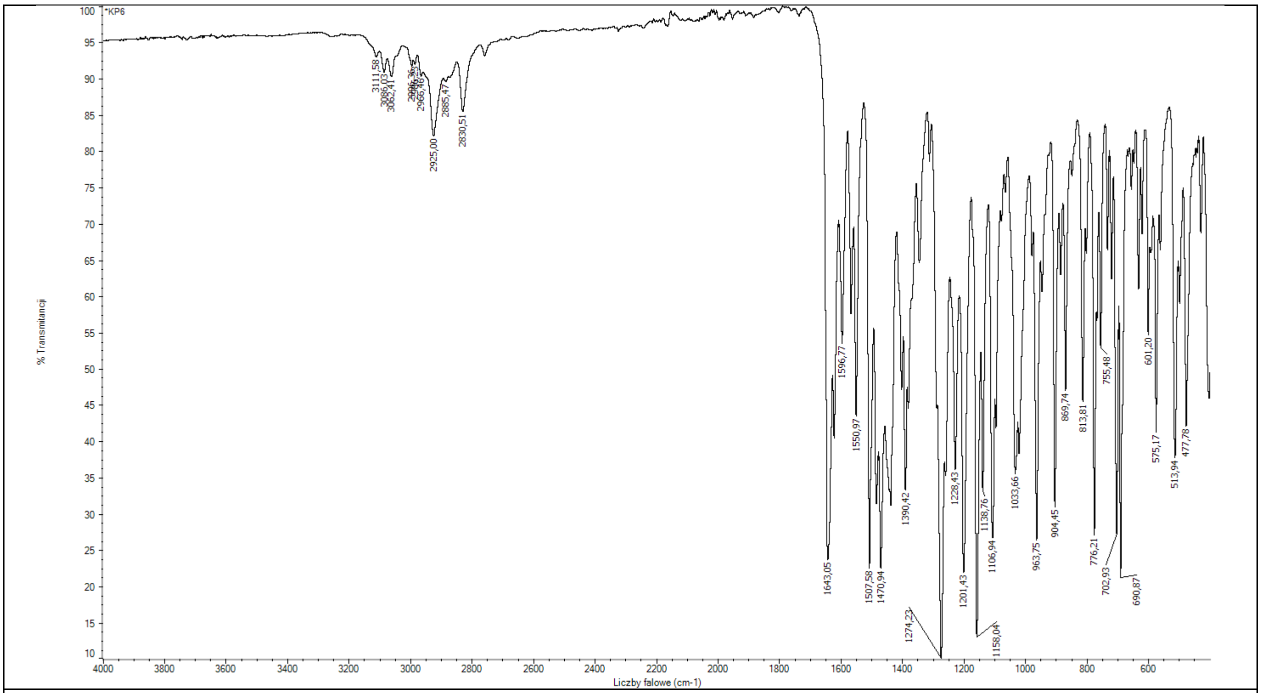
*KP6
% Transmitancji
Liczby falowe (cm-1)
3111,58
3086,02
3062,41
2996,36
2966,46
2925,00
2885,47
2830,51
1643,05
1596,77
1550,97
1507,58
1470,94
1390,42
1274,23
1228,43
1201,43
1158,04
1138,76
1106,94
1033,66
963,75
904,45
869,74
813,81
776,21
755,48
702,93
690,87
601,20
575,17
513,94
477,78

6a

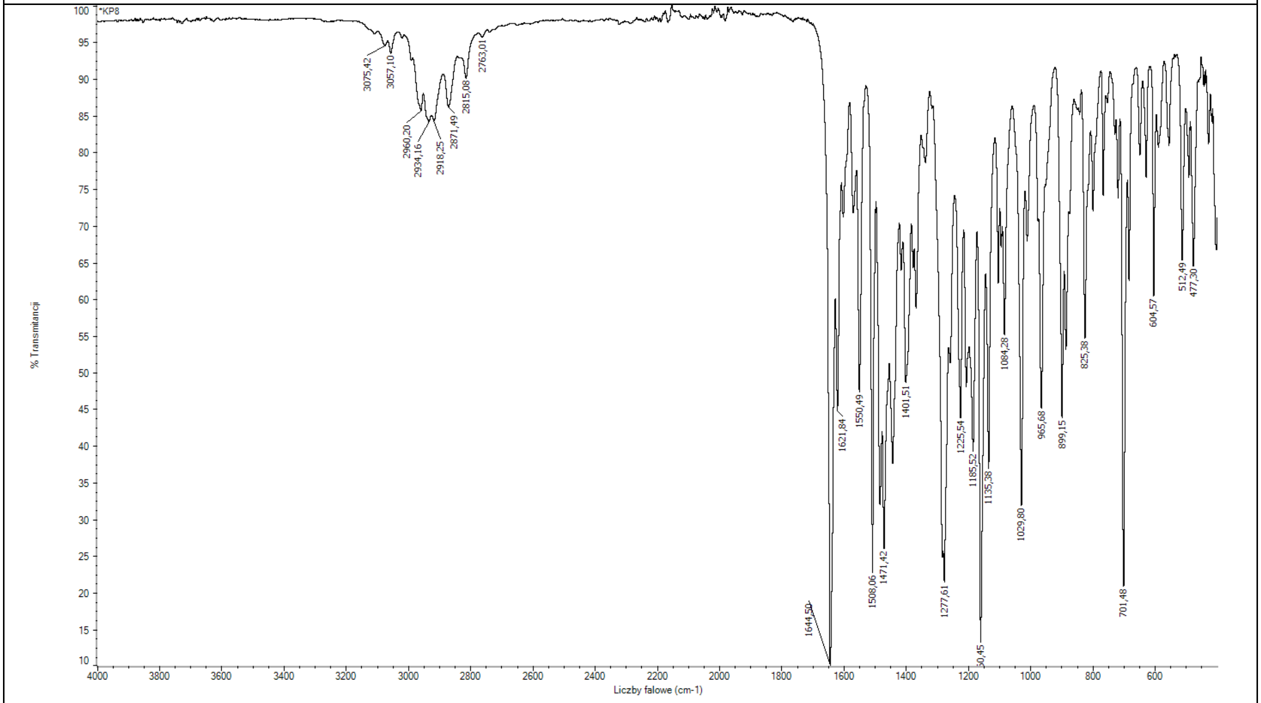
*KP8
% Transmitancji
Liczby falowe (cm-1)
3075,42
3057,10
2960,20
2934,16
2918,25
2871,49
2815,08
2763,01
1644,50
1621,84
1550,49
1508,06
1471,42
1401,51
1277,61
1225,54
1185,52
1135,38
1084,28
1029,80
965,68
899,15
825,38
701,48
604,57
512,49
477,30

6b

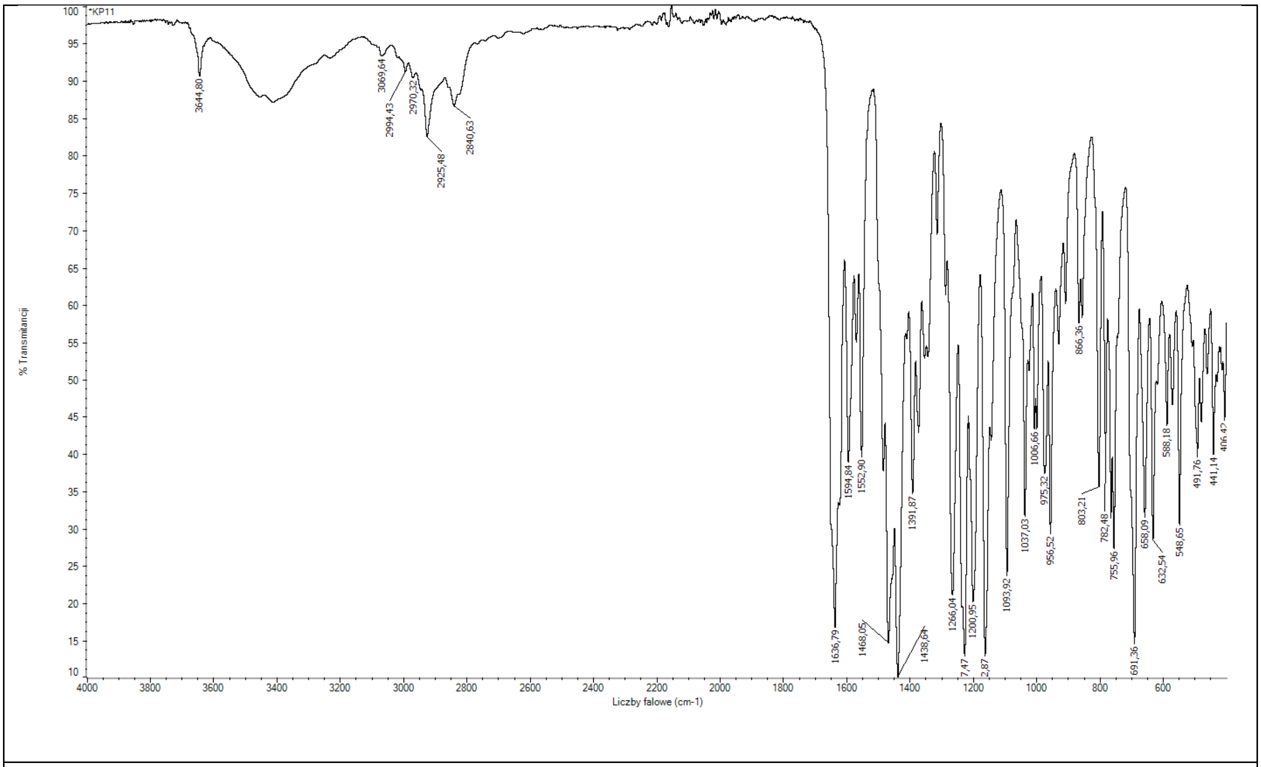
*KP11
% Transmitancji
Liczby falowe (cm-1)
3644,80
3069,64
2994,43
2970,32
2925,48
2840,63
1636,79
1594,84
1552,90
1468,05
1438,64
1391,87
1266,04
7,47
1200,95
2,87
1093,92
1037,03
1006,66
975,32
956,52
866,36
803,21
782,48
755,96
691,36
658,09
632,54
588,18
548,65
491,76
441,14
406,42

7a

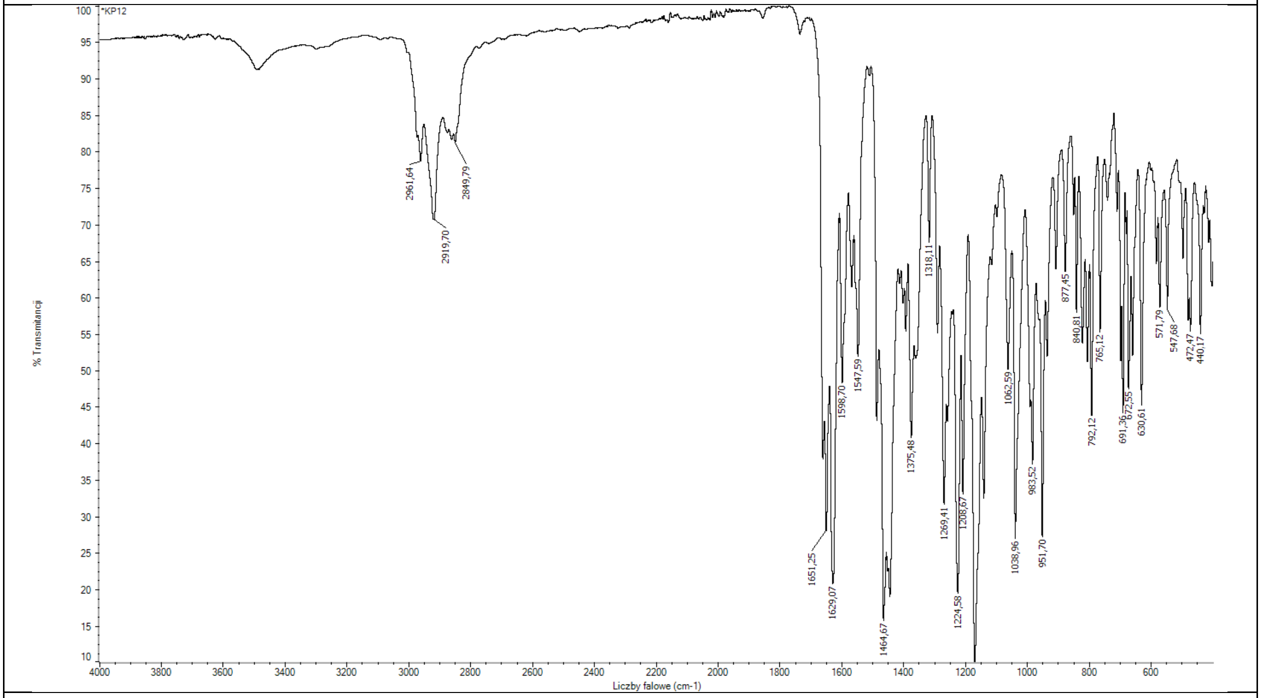
*KP12
% Transmitancji
Liczby falowe (cm-1)
2961,64
2919,70
2849,79
1651,25
1629,07
1598,70
1547,59
1464,67
1375,48
1318,11
1269,41
1224,58
1208,67
1062,59
1038,96
983,52
951,70
877,45
840,81
792,12
765,12
691,36
672,55
630,61
571,79
547,68
472,47
440,17

7b

Table S4. Mass spectra of new compounds

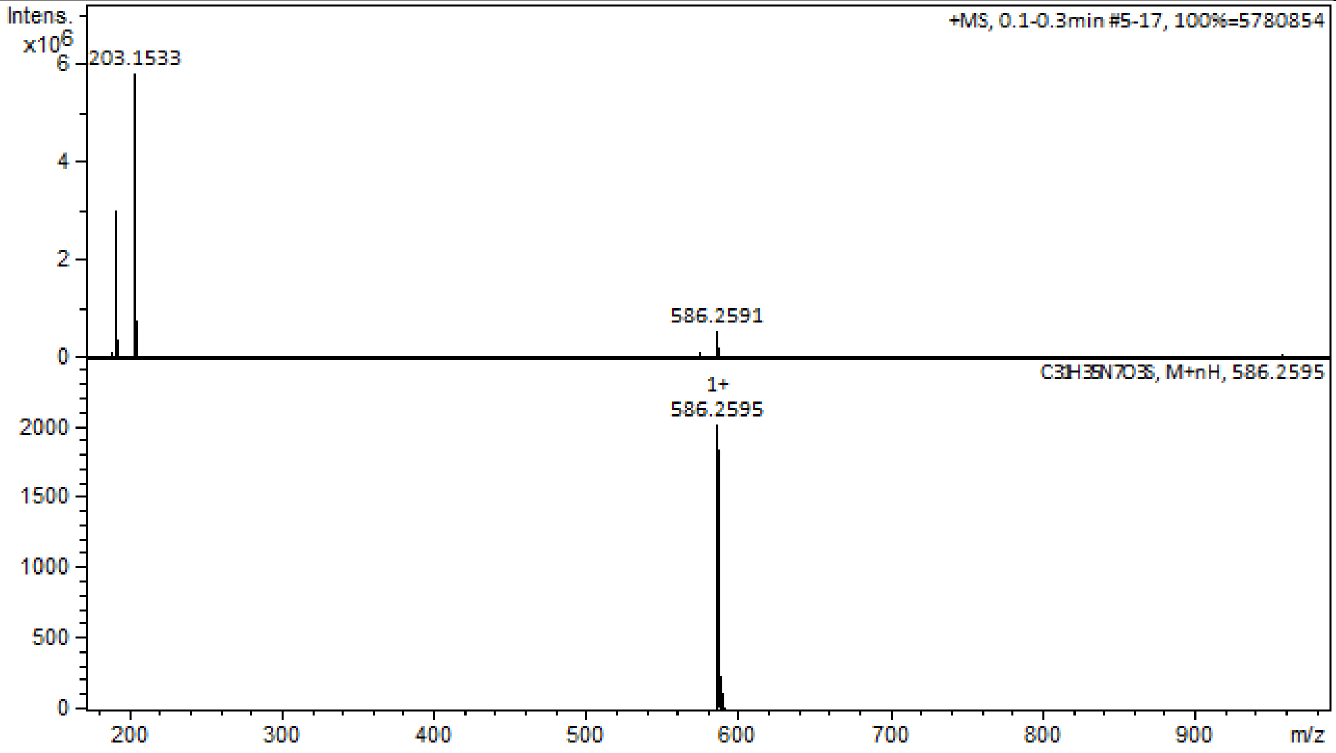

2a

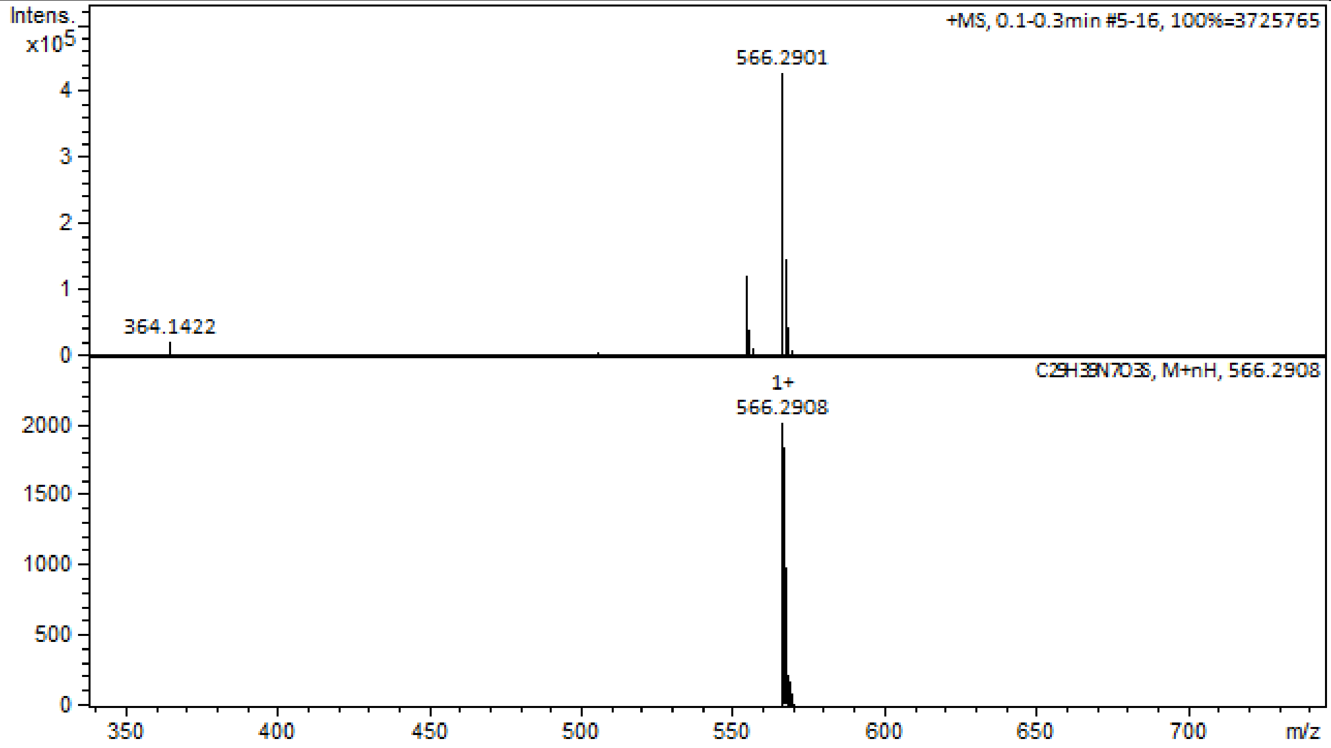

2b

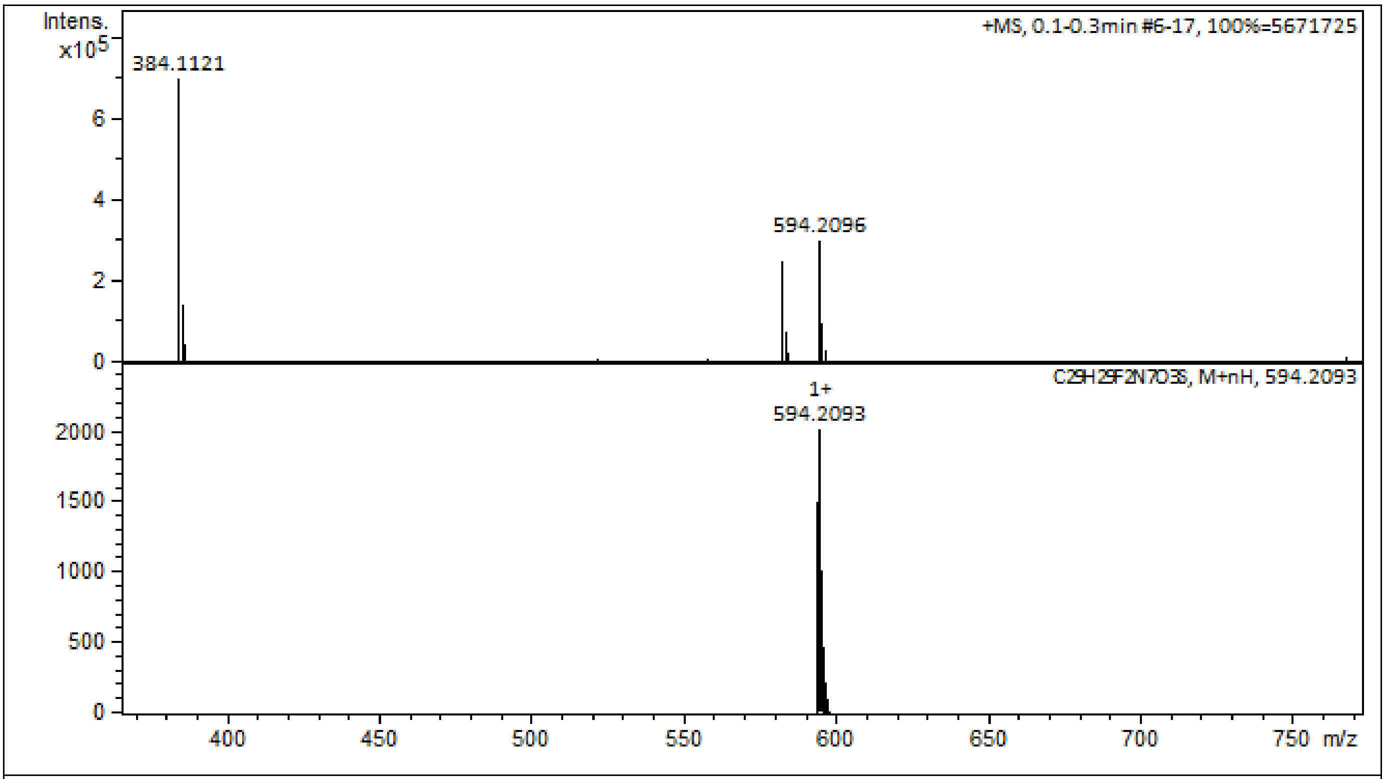

Intens.
x10^5
+MS, 0.1-0.3min #6-17, 100%=5671725
384.1121
594.2096
C29H29F2N7O3S, M+nH, 594.2093
1+
594.2093
m/z

3a

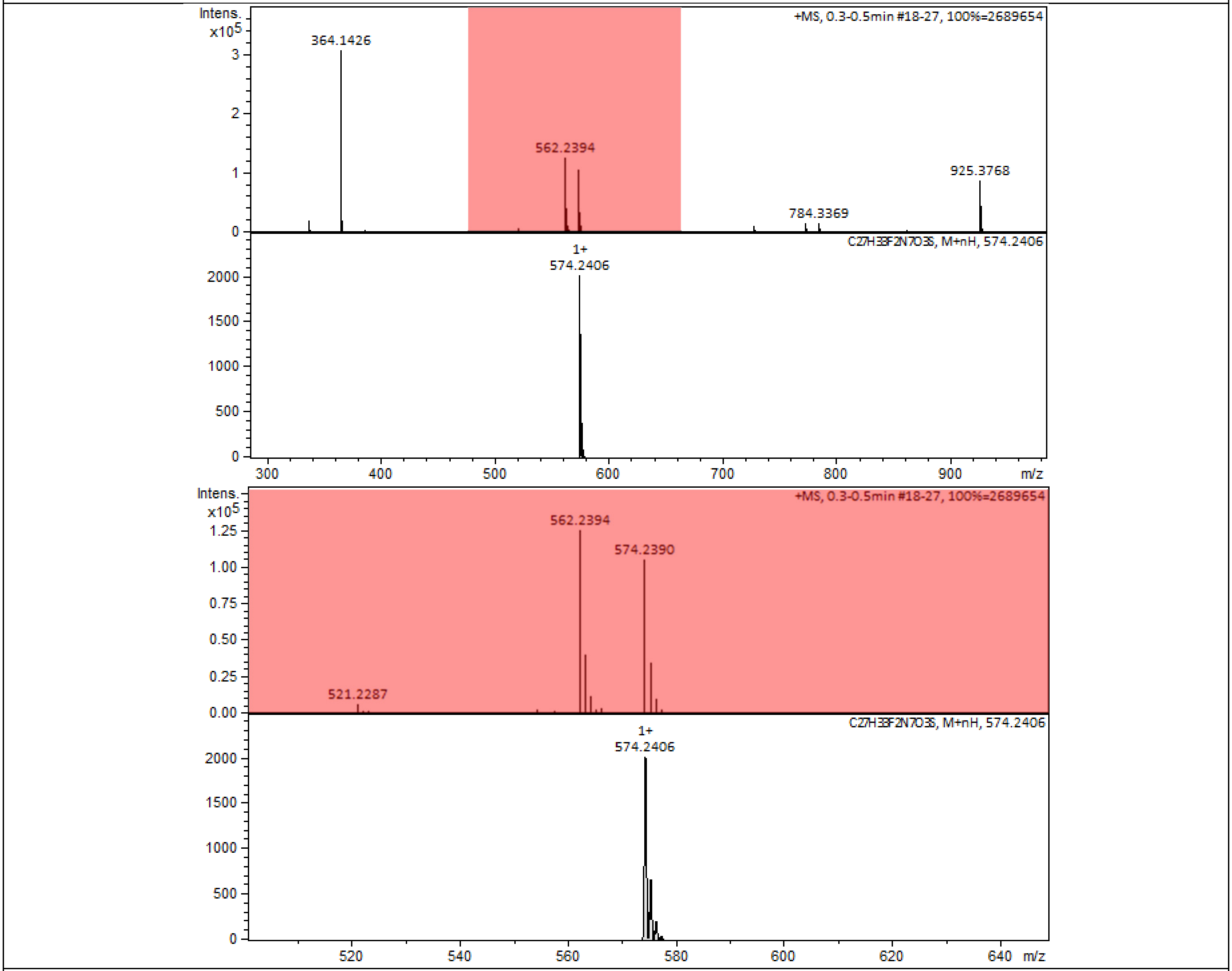

Intens.
x10^5
+MS, 0.3-0.5min #18-27, 100%=2689654
364.1426
562.2394
925.3768
784.3369
C27H33F2N7O3S, M+nH, 574.2406
1+
574.2406
m/z
Intens.
x10^5
+MS, 0.3-0.5min #18-27, 100%=2689654
562.2394
574.2390
521.2287
C27H33F2N7O3S, M+nH, 574.2406
1+
574.2406
m/z

3b

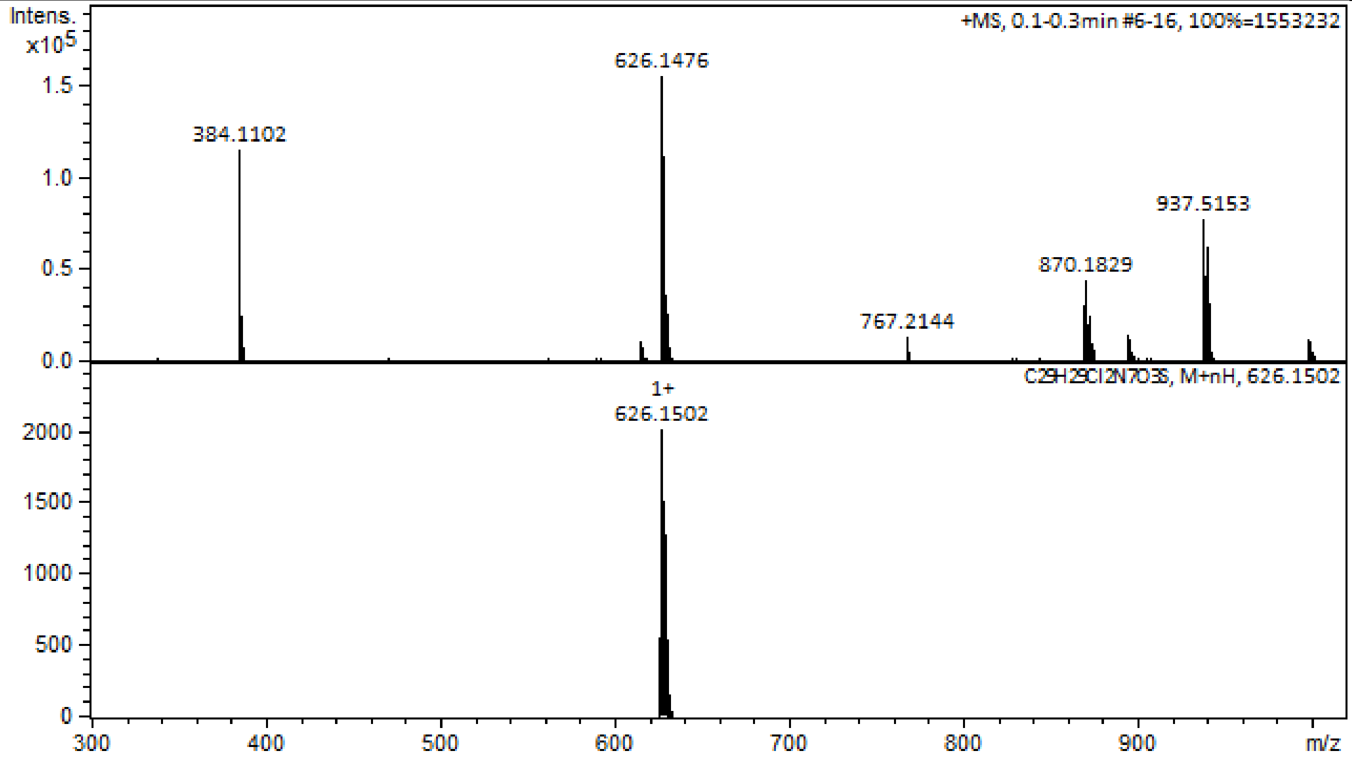
Intens.
x10^5
+MS, 0.1-0.3min #6-16, 100%=1553232
626.1476
384.1102
937.5153
870.1829
767.2144
C29H29Cl2N7O3S, M+nH, 626.1502
1+
626.1502
m/z

4a

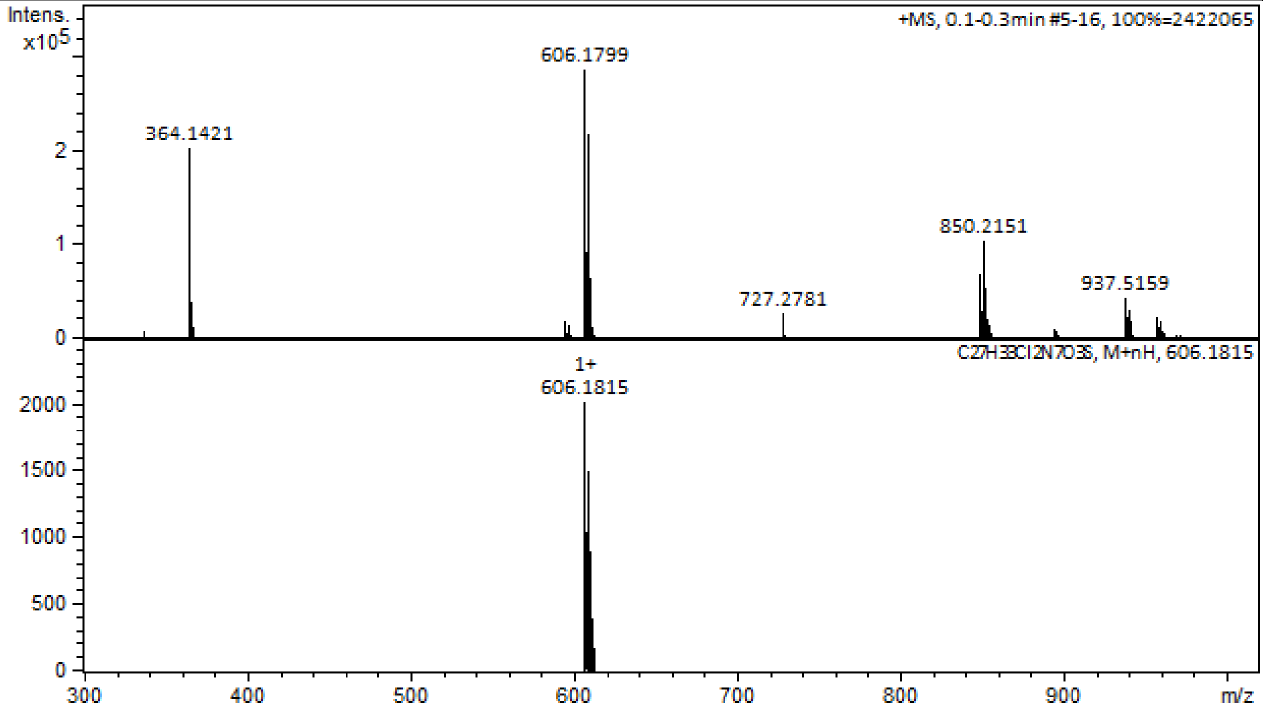
Intens.
x10^5
+MS, 0.1-0.3min #5-16, 100%=2422065
606.1799
364.1421
850.2151
937.5159
727.2781
C27H33Cl2N7O3S, M+nH, 606.1815
1+
606.1815
m/z

4b

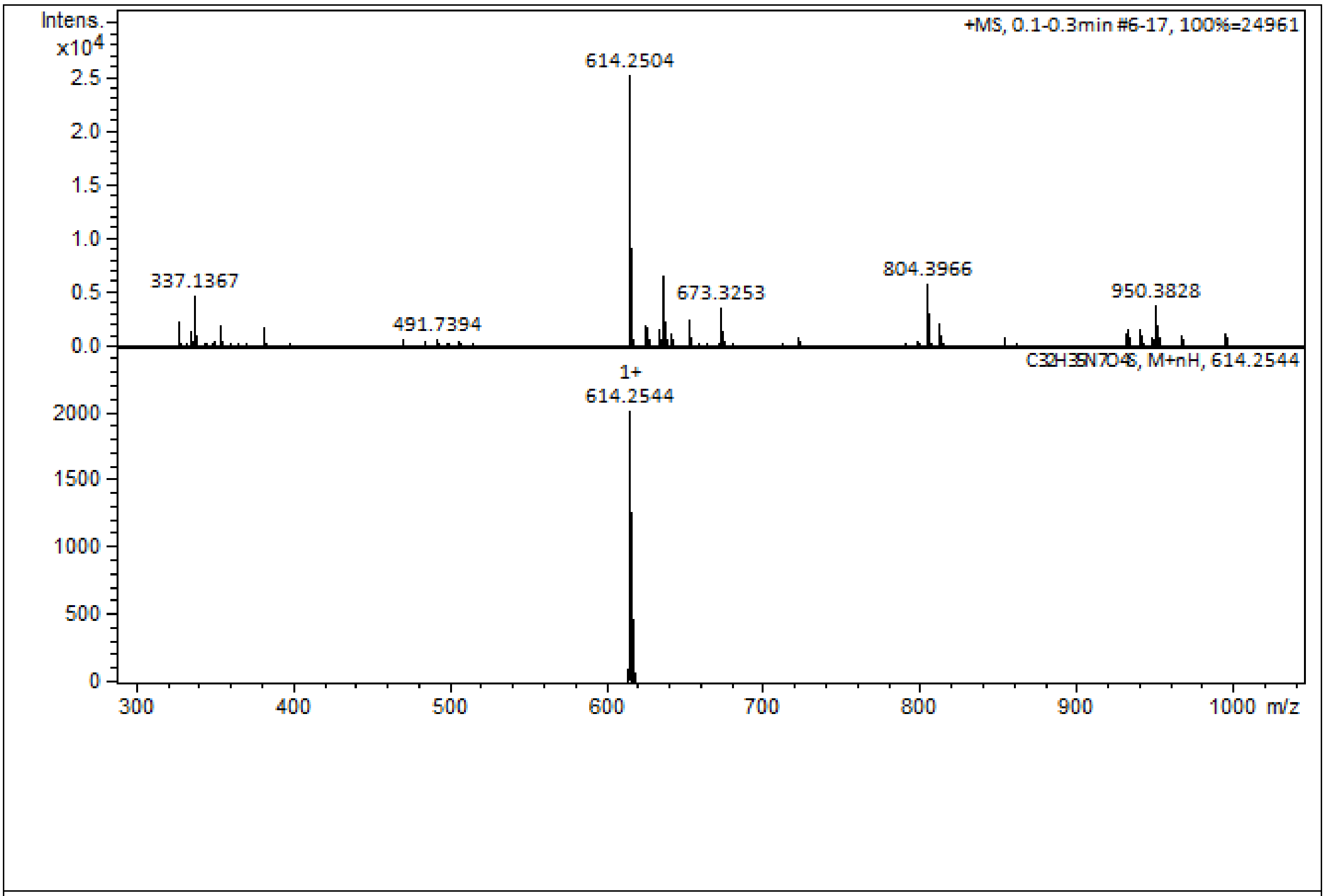
Intens.
x10^4
2.5
2.0
1.5
1.0
0.5
0.0
+MS, 0.1-0.3min #6-17, 100%=24961
614.2504
337.1367
491.7394
673.3253
804.3966
950.3828
C32H35N7O4S, M+nH, 614.2544
1+
614.2544
2000
1500
1000
500
0
300
400
500
600
700
800
900
1000
m/z

5a

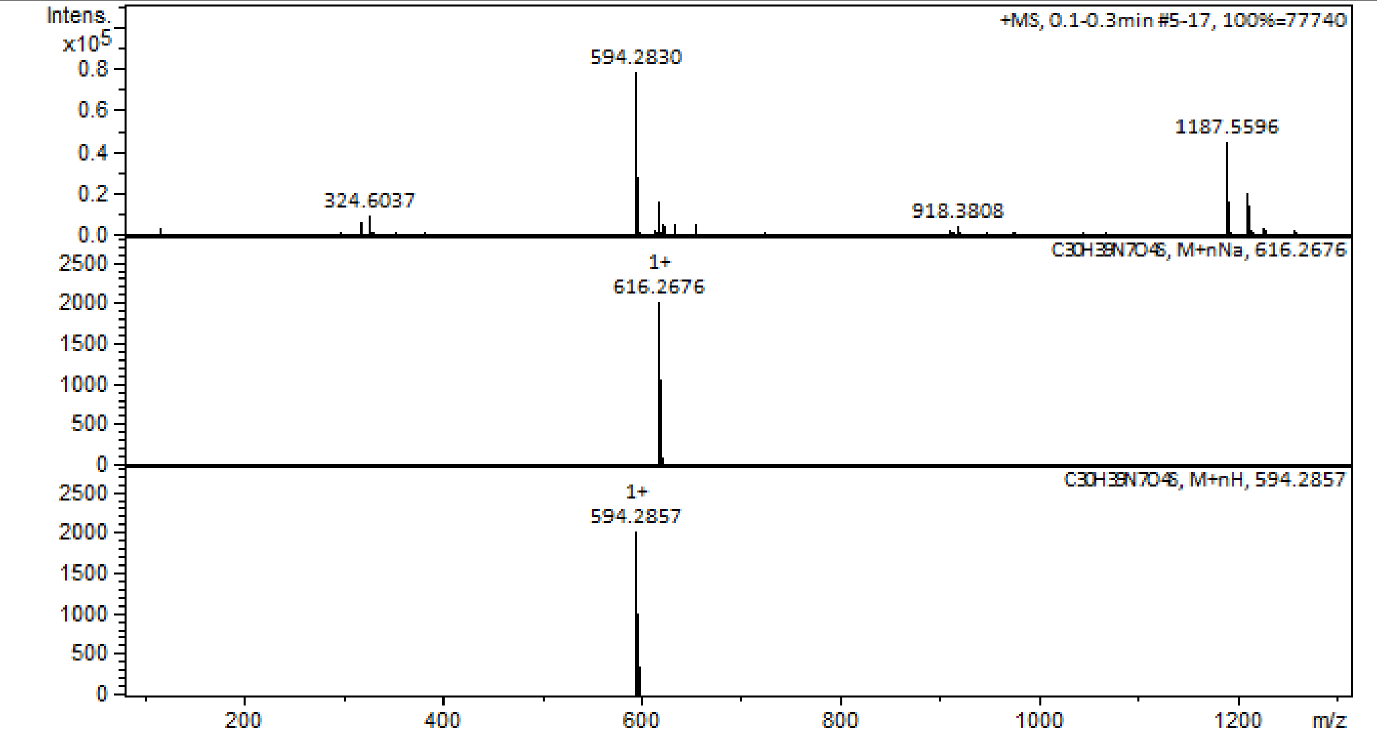
Intens.
x10^5
0.8
0.6
0.4
0.2
0.0
+MS, 0.1-0.3min #5-17, 100%=77740
594.2830
324.6037
918.3808
1187.5596
C30H39N7O4S, M+nNa, 616.2676
1+
616.2676
2500
2000
1500
1000
500
0
C30H39N7O4S, M+nH, 594.2857
1+
594.2857
2500
2000
1500
1000
500
0
200
400
600
800
1000
1200
m/z

5b

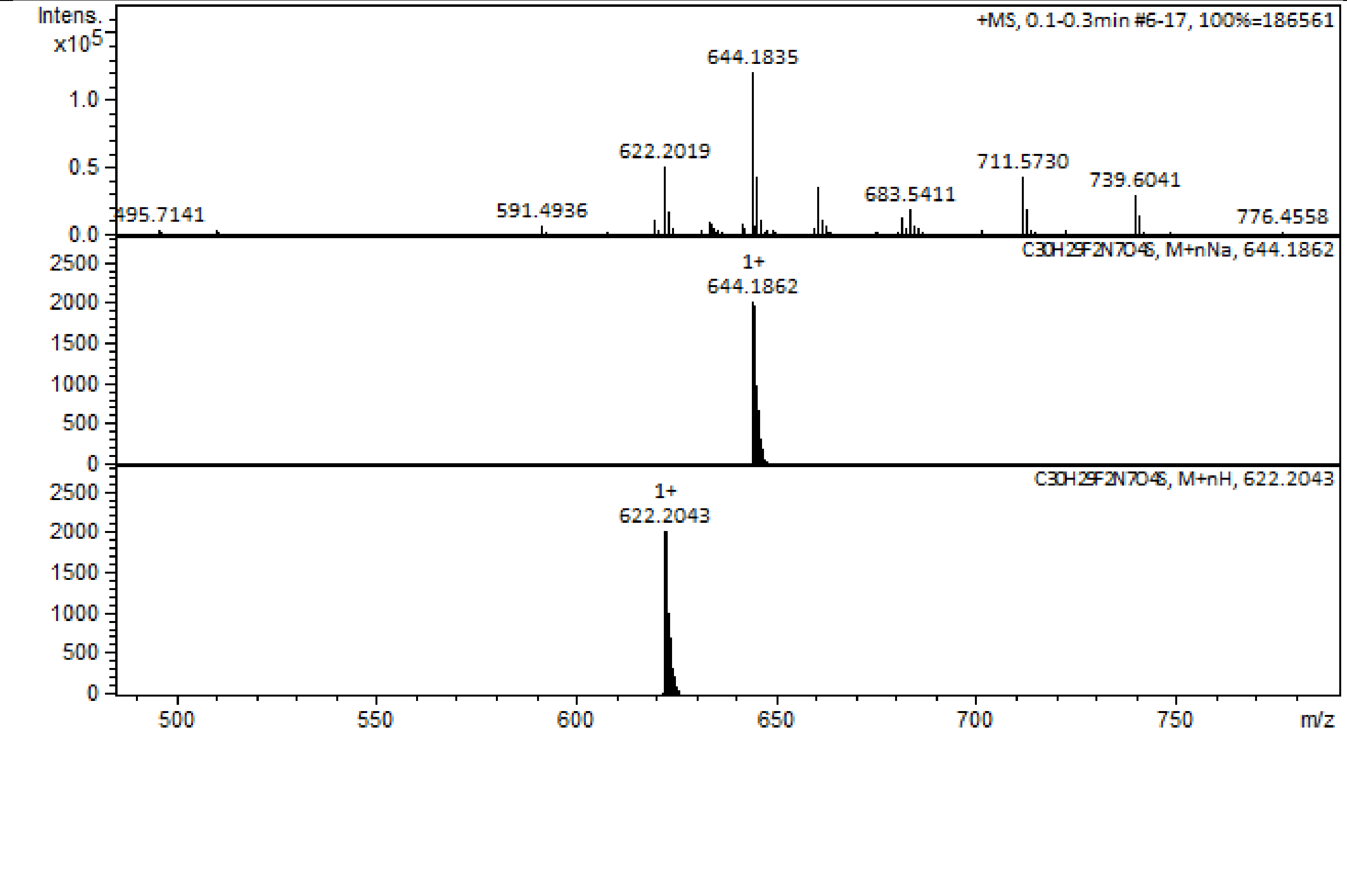
Intens.
x10^5
1.0
0.5
0.0
+MS, 0.1-0.3min #6-17, 100%=186561
644.1835
622.2019
711.5730
739.6041
683.5411
495.7141
591.4936
776.4558
C30H29F2N7O4S, M+nNa, 644.1862
1+
644.1862
2500
2000
1500
1000
500
0
C30H29F2N7O4S, M+nH, 622.2043
1+
622.2043
500
550
600
650
700
750
m/z

6a

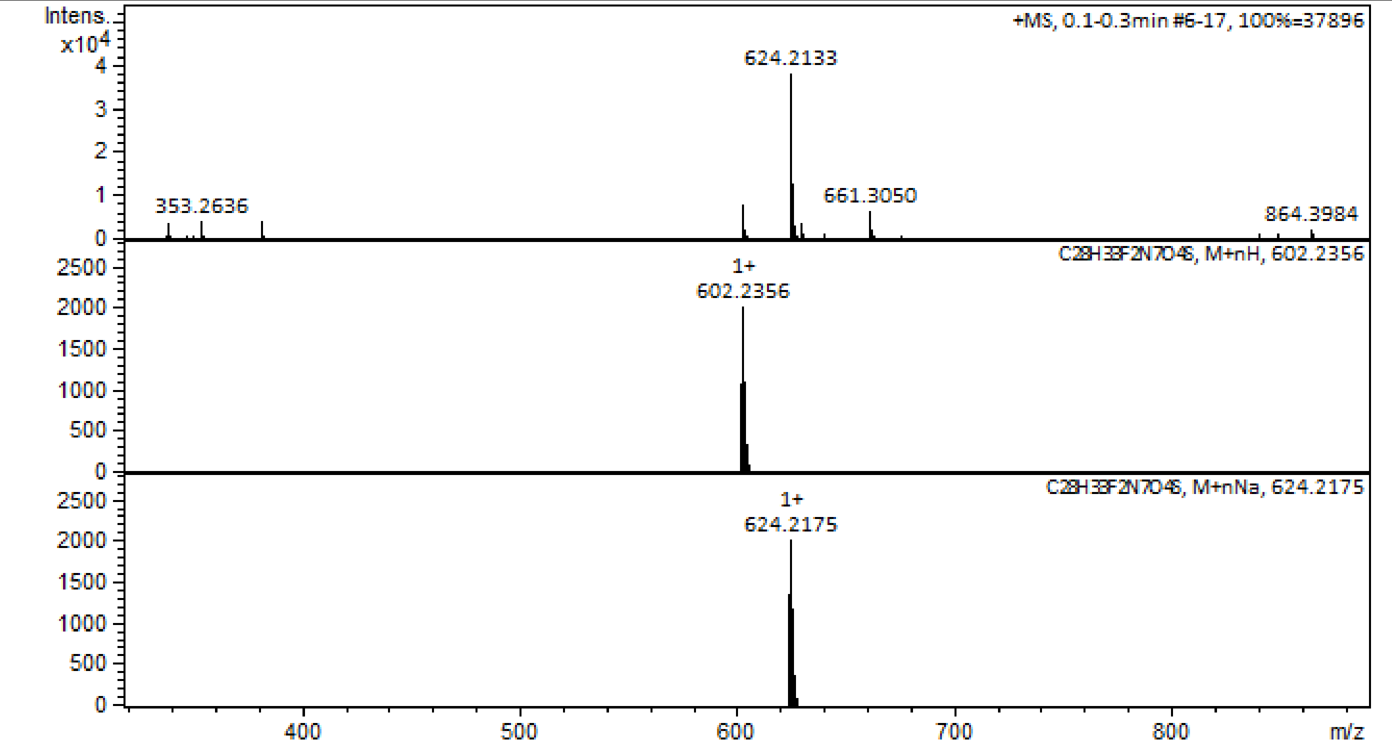
Intens.
x10^4
4
3
2
1
0
+MS, 0.1-0.3min #6-17, 100%=37896
624.2133
353.2636
661.3050
864.3984
C28H33F2N7O4S, M+nH, 602.2356
1+
602.2356
2500
2000
1500
1000
500
0
C28H33F2N7O4S, M+nNa, 624.2175
1+
624.2175
400
500
600
700
800
m/z

6b

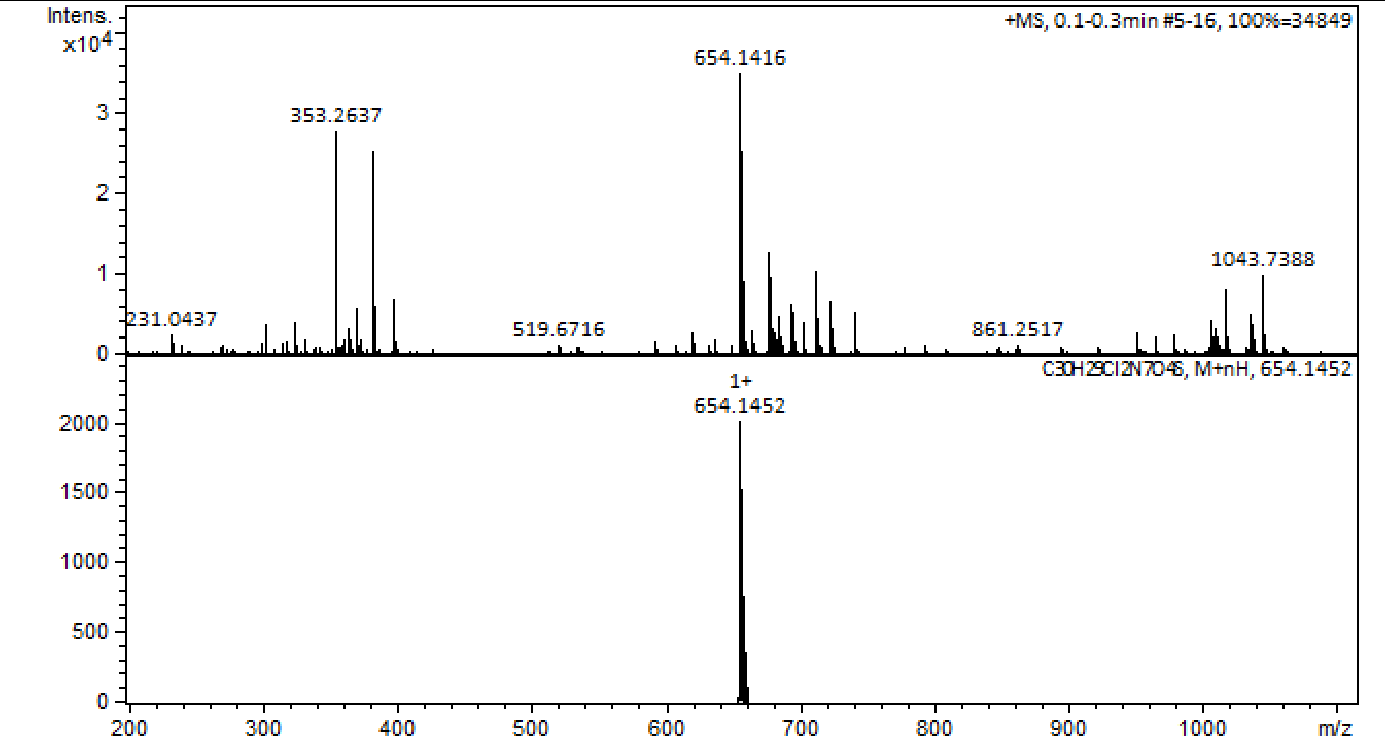

Intens.
x10^4
+MS, 0.1-0.3min #5-16, 100%=34849
654.1416
353.2637
1043.7388
231.0437
519.6716
861.2517
C30H29Cl2N7O4S, M+nH, 654.1452
1+
654.1452
m/z

7a

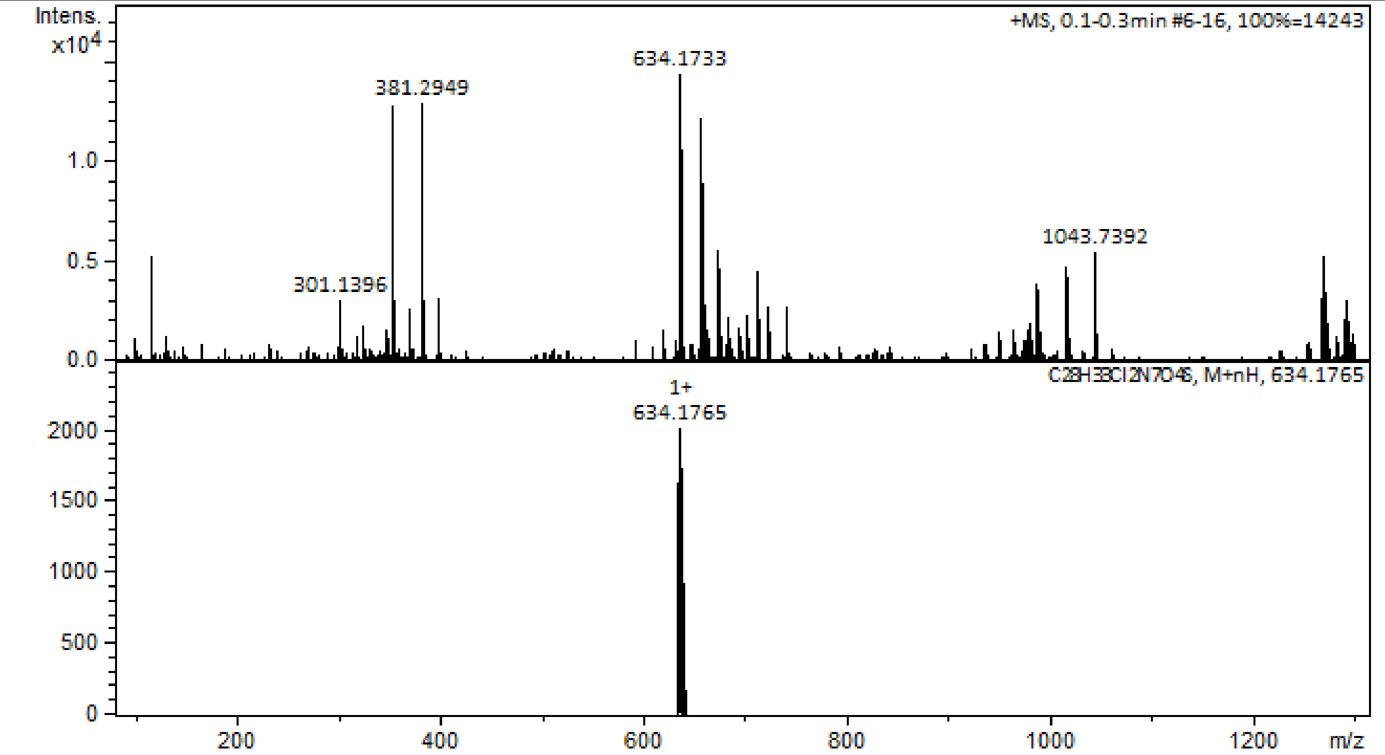

Intens.
x10^4
+MS, 0.1-0.3min #6-16, 100%=14243
634.1733
381.2949
1043.7392
301.1396
C28H33Cl2N7O4S, M+nH, 634.1765
1+
634.1765
m/z

7b

Table S5. The 2D intermolecular interactions of investigated compounds in the active site of COX

Figure S1. The intermolecular interactions of **2a** in the active centre of a) COX-1 b) COX-2

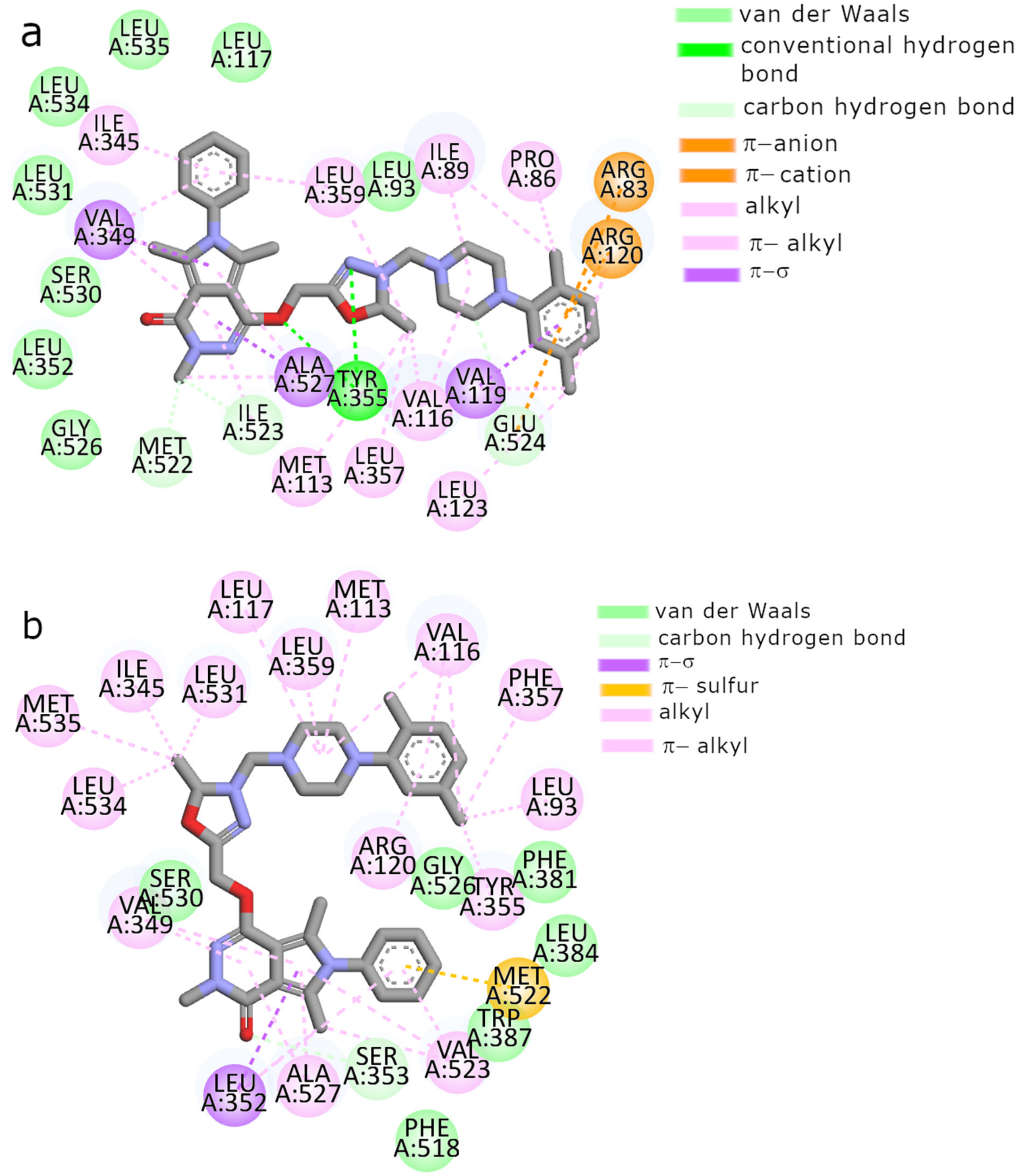

Figure S2. The intermolecular interactions of **2b** in the active centre of a) COX-1 b) COX-2

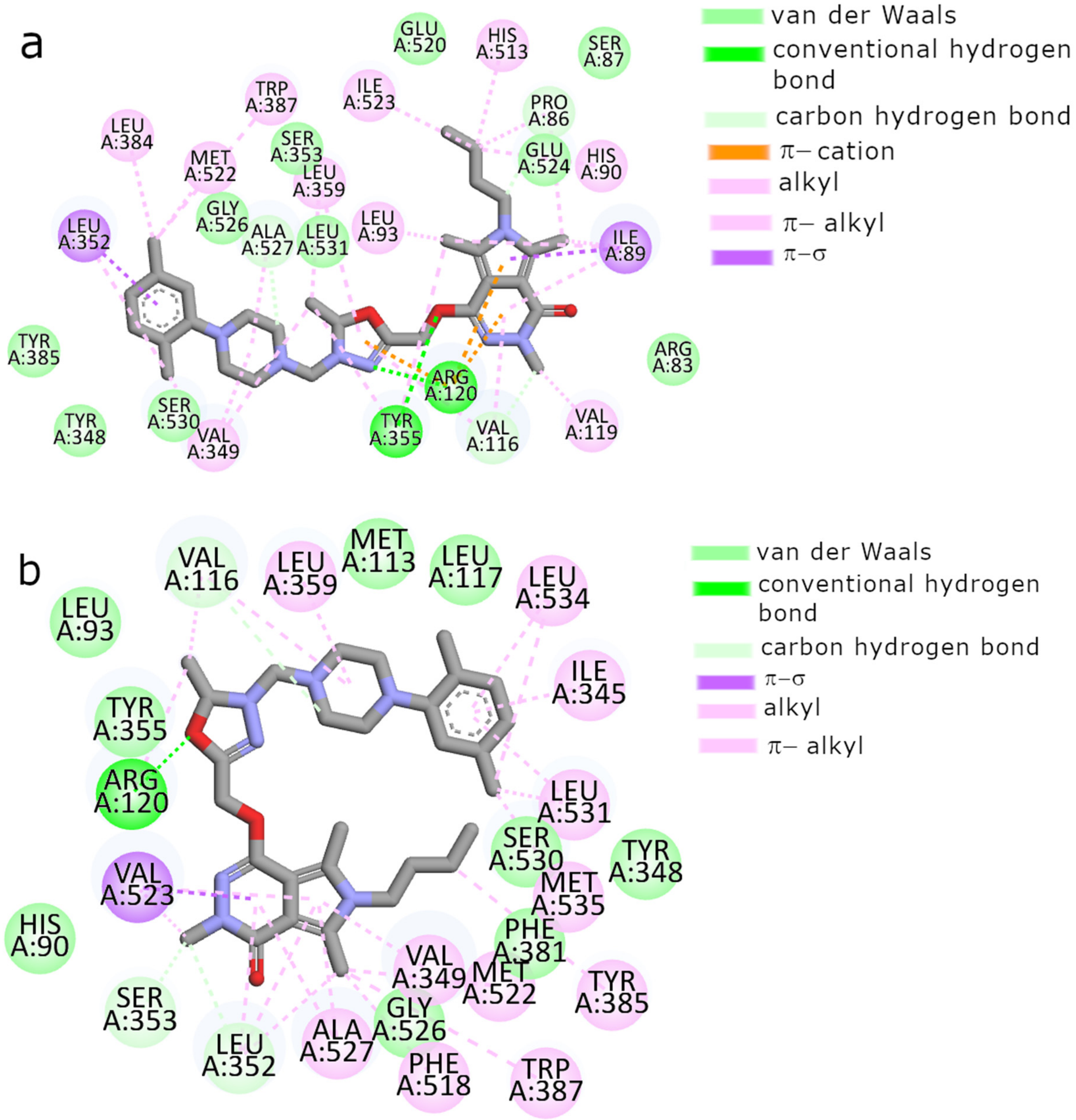

Figure S3. The intermolecular interactions of **3a** in the active centre of a) COX-1 b) COX-2

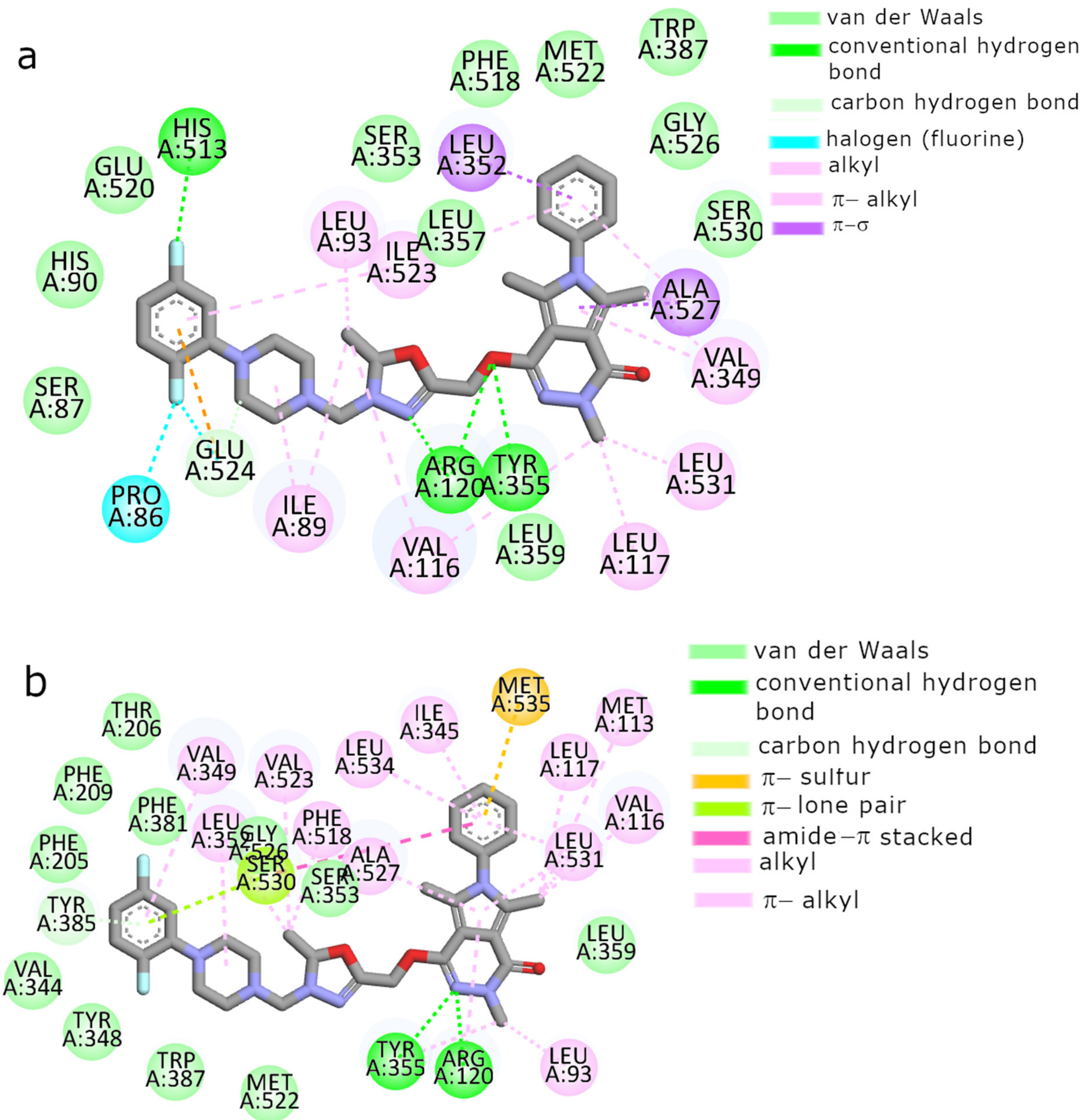

Figure S4. The intermolecular interactions of **3b** in the active centre of a) COX-1 b) COX-2

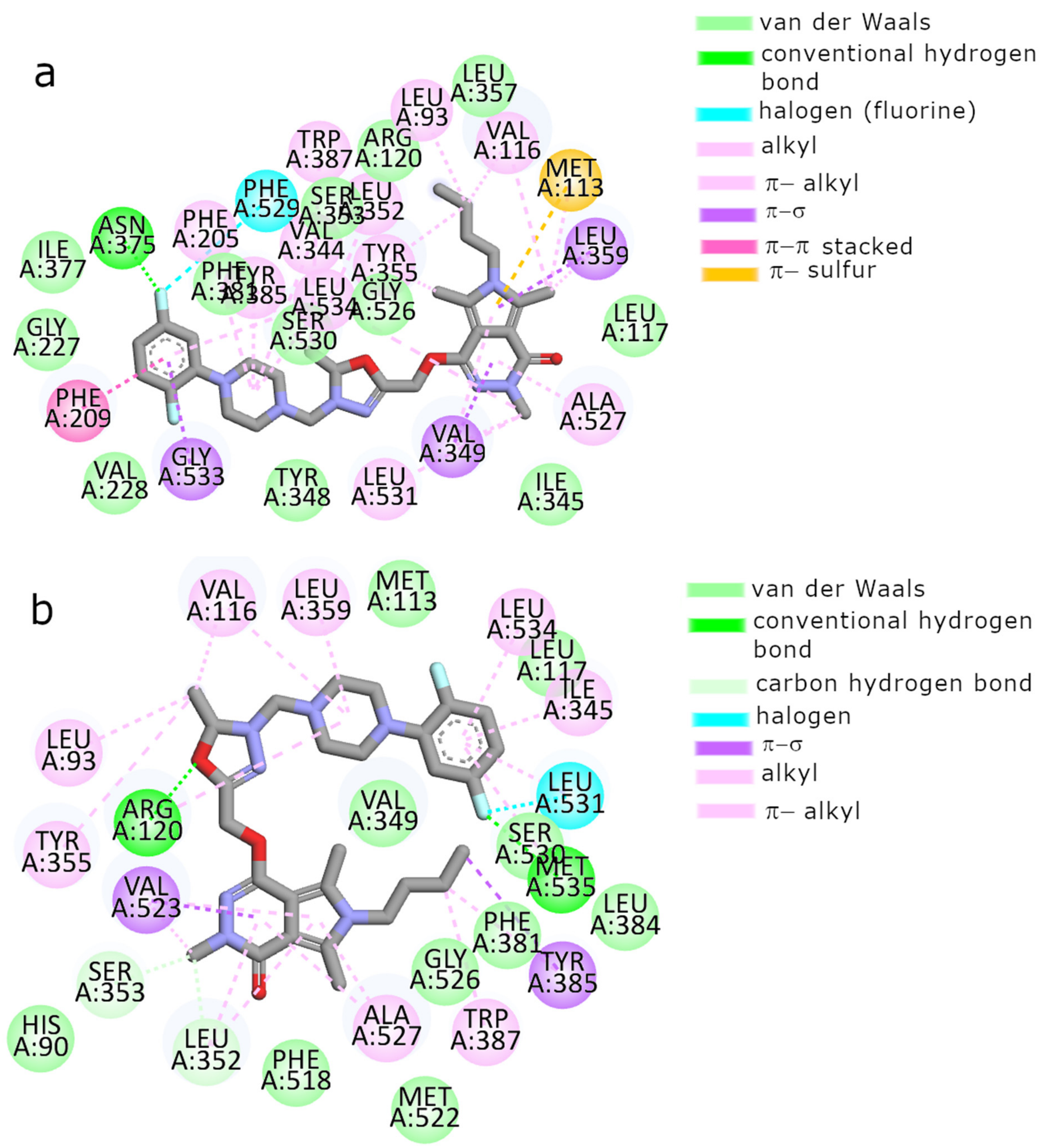

Figure S5. The intermolecular interactions of **4a** in the active centre of a) COX-1 b) COX-2

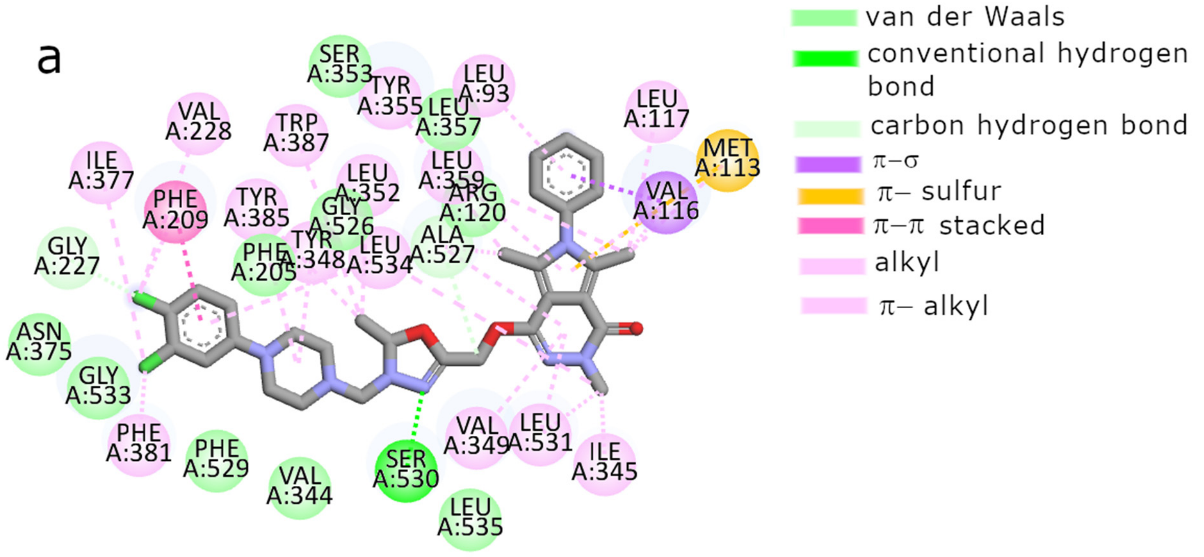

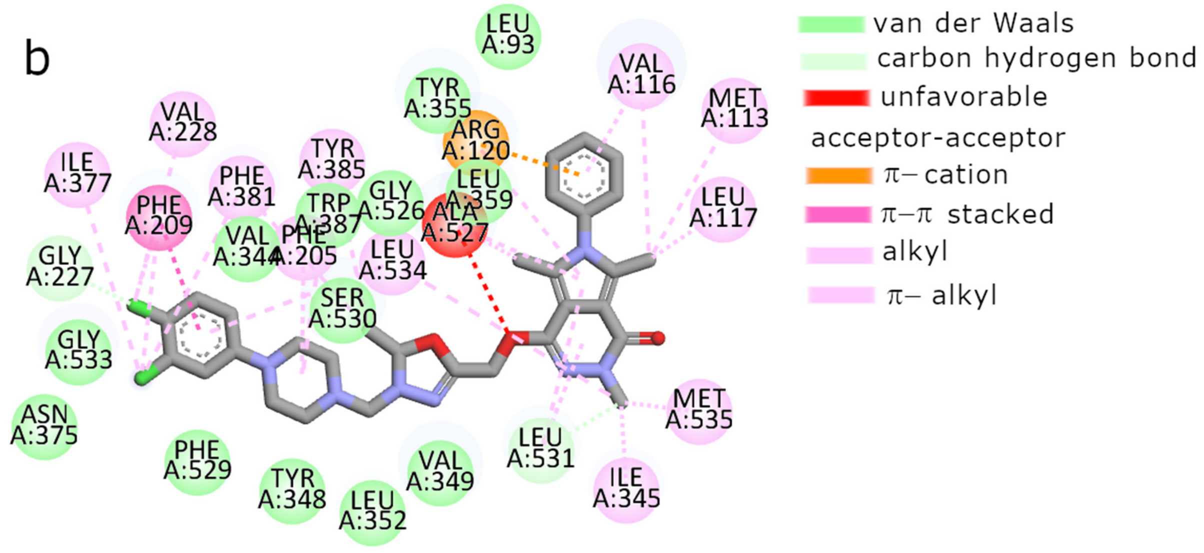

Figure S6. The intermolecular interactions of **4b** in the active centre of a) COX-1 b) COX-2

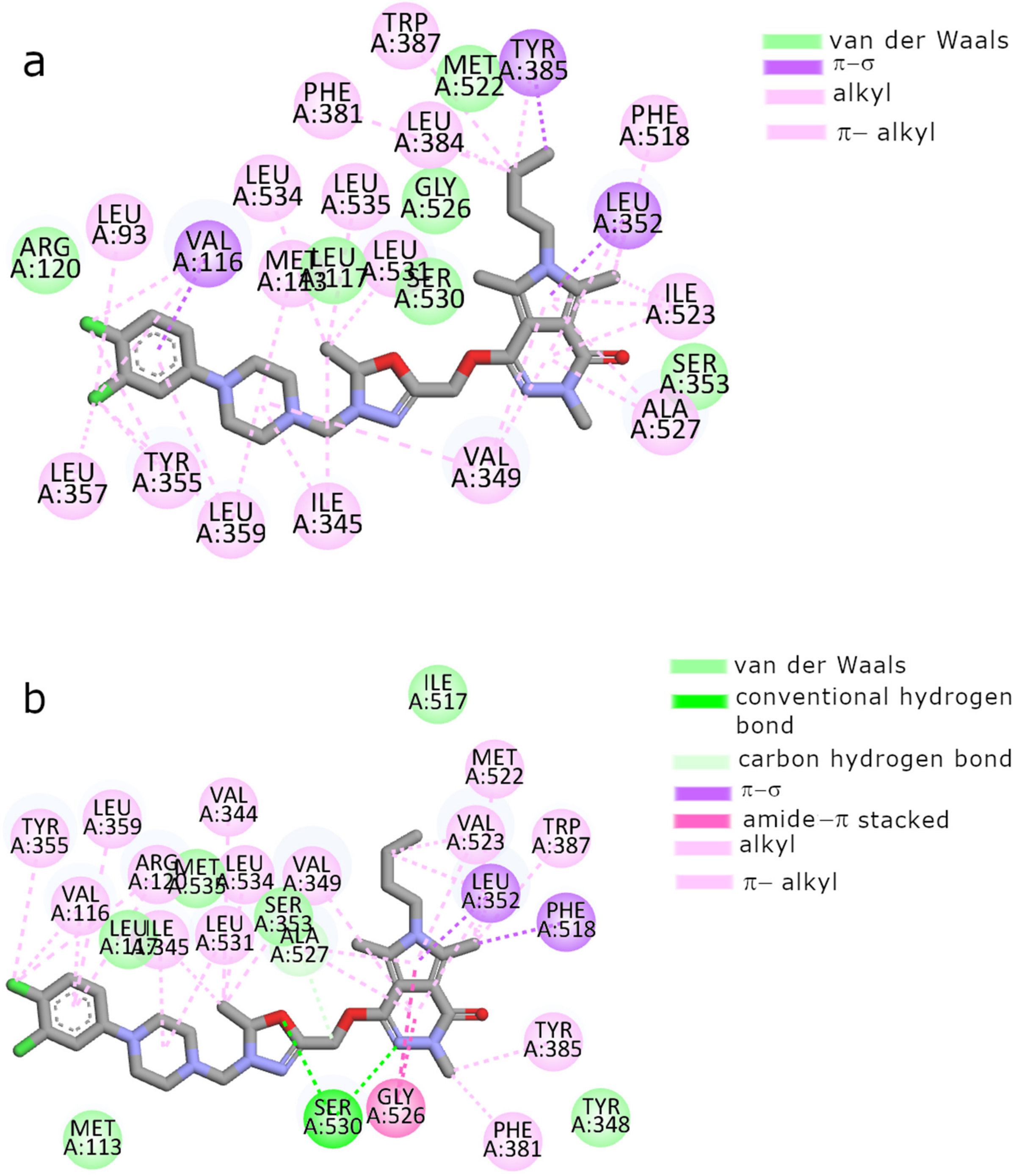

Figure S7. The intermolecular interactions of **5a** in the active centre of a) COX-1 b) COX-2

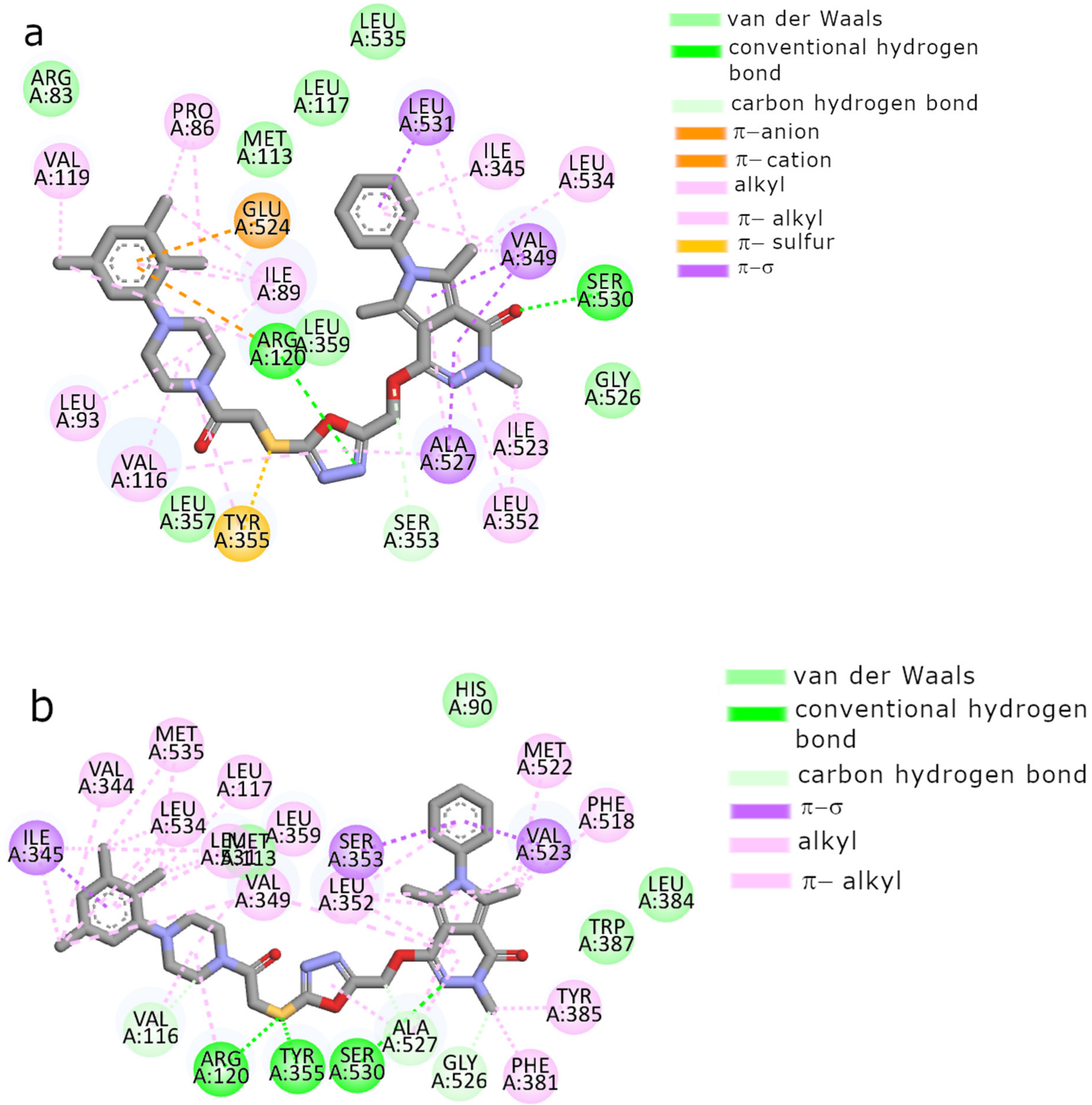

Figure S8. The intermolecular interactions of **5b** in the active centre of a) COX-1 b) COX-2

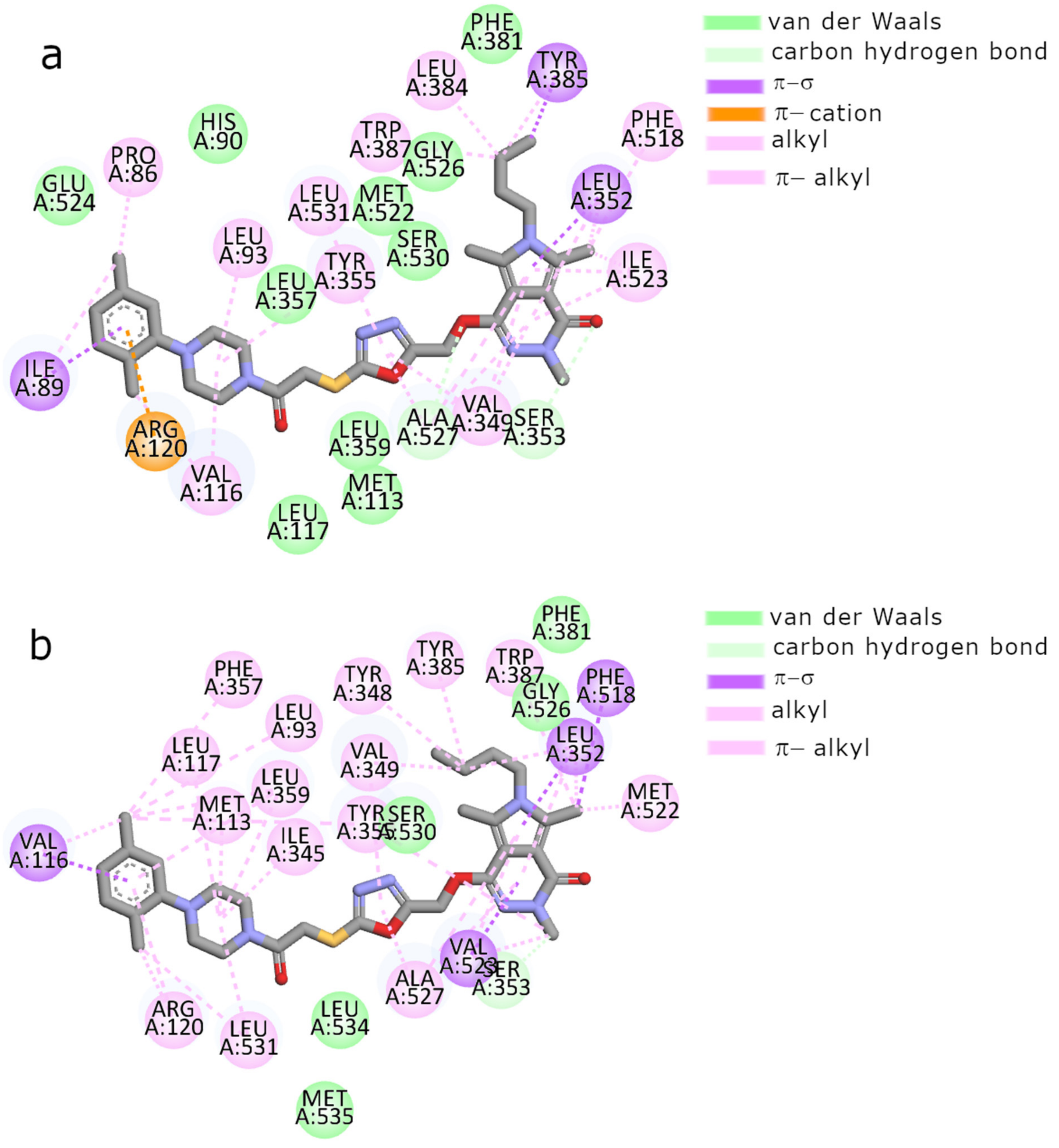

Figure S9. The intermolecular interactions of **6a** in the active centre of a) COX-1 b) COX-2

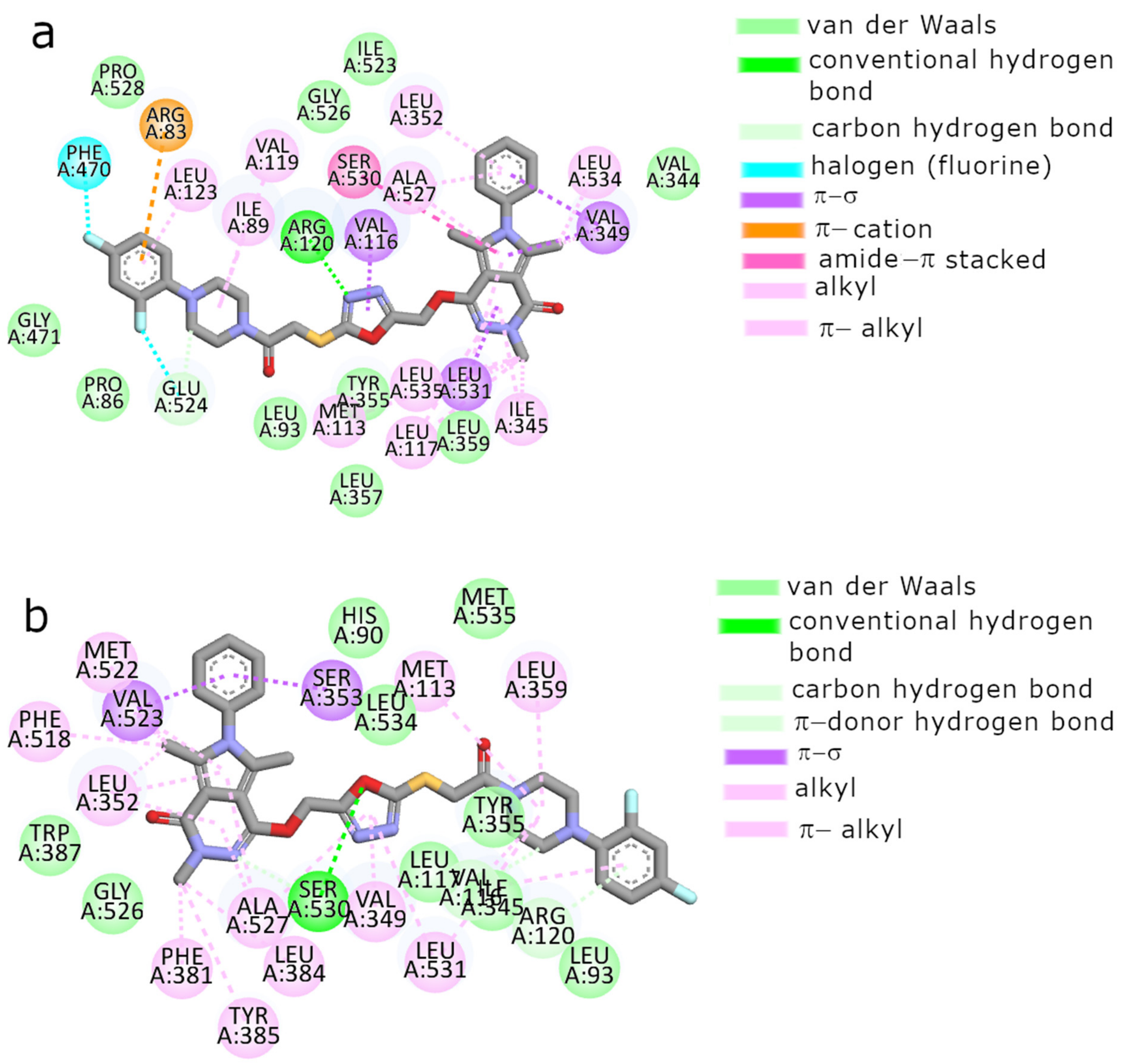

Figure S10. The intermolecular interactions of **6b** in the active centre of a) COX-1 b) COX-2

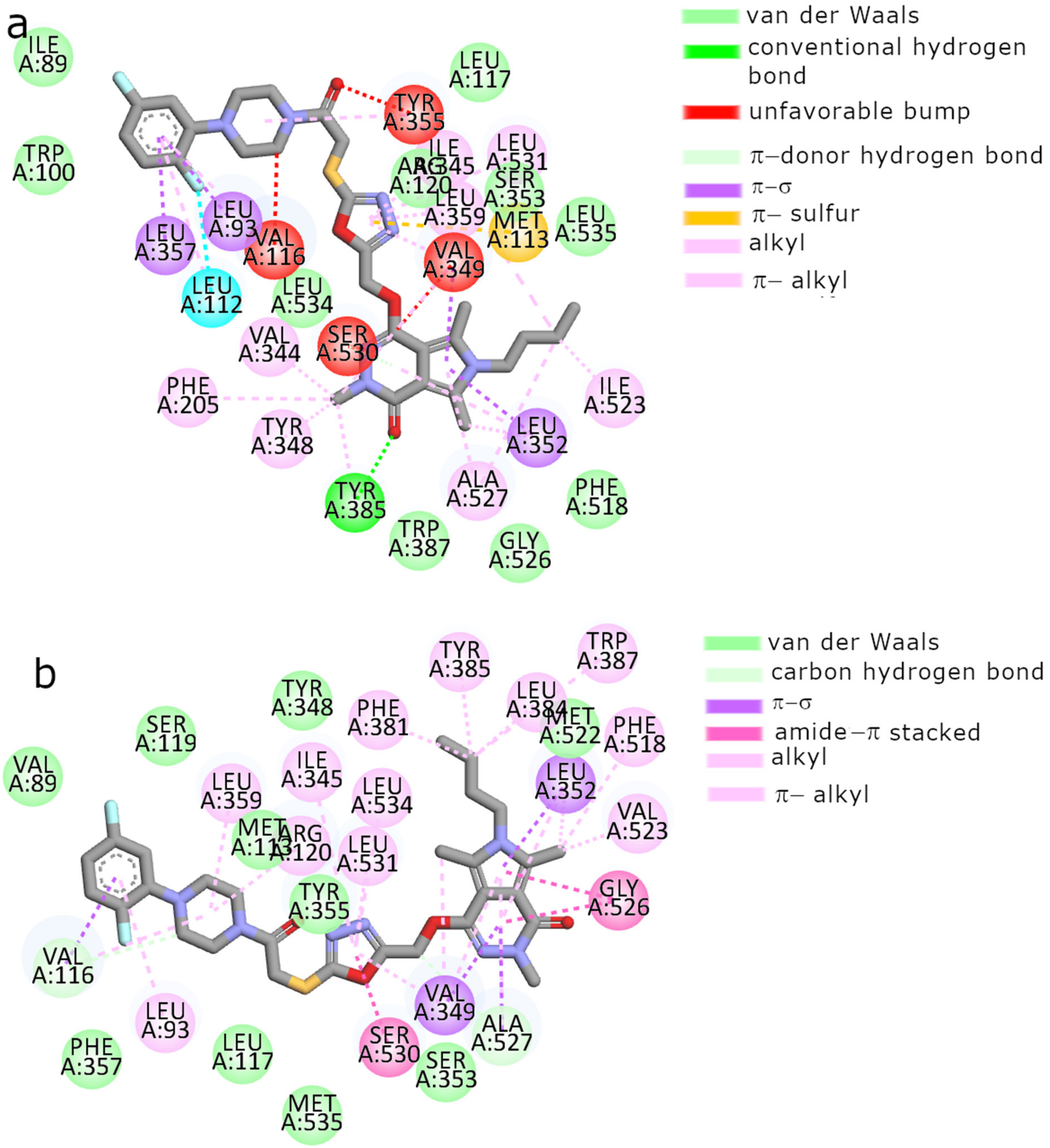

Figure S11. The intermolecular interactions of **7a** in the active centre of a) COX-1 b) COX-2

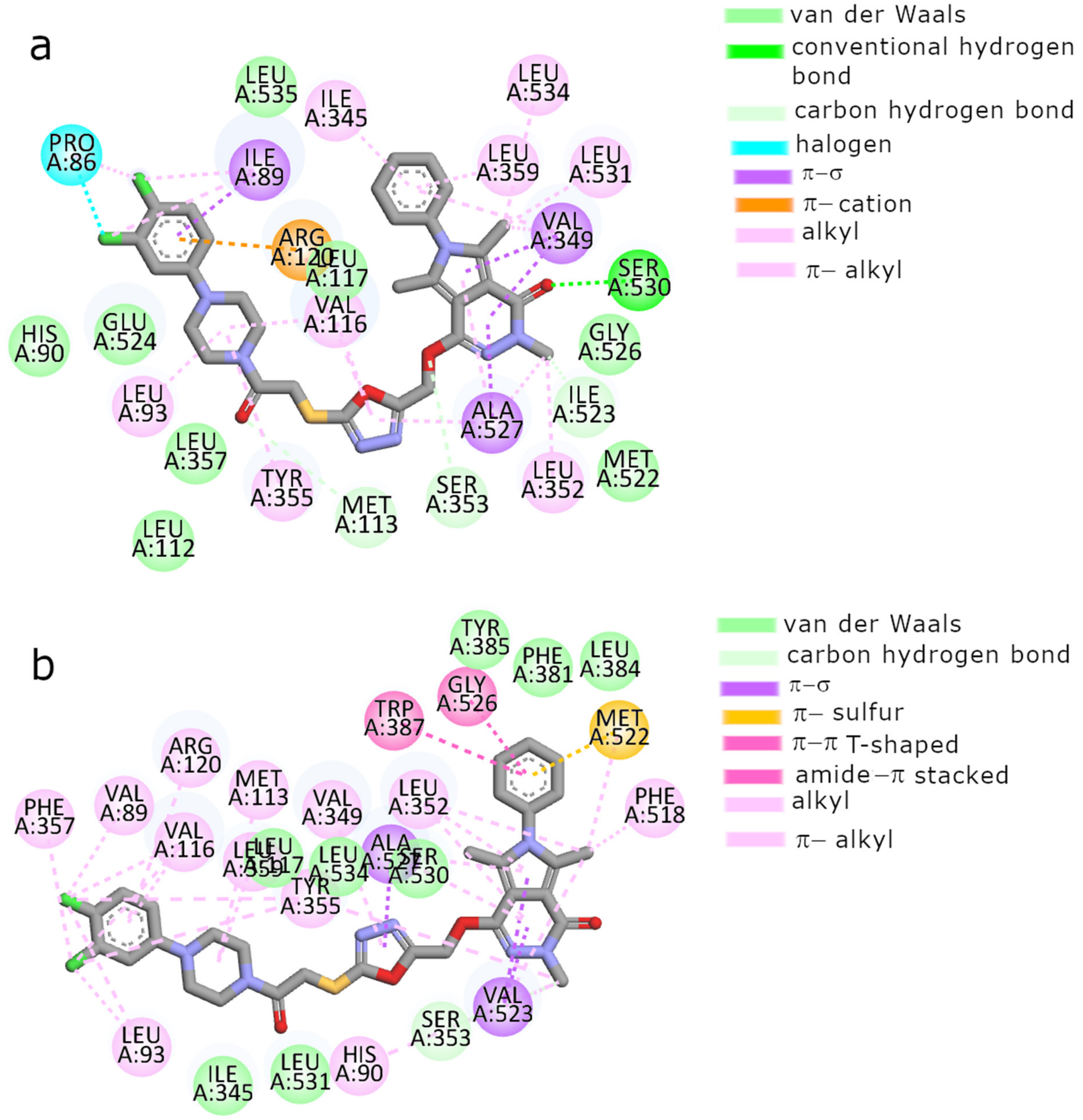

Figure S12. The intermolecular interactions of **7b** in the active centre of a) COX-1 b) COX-2

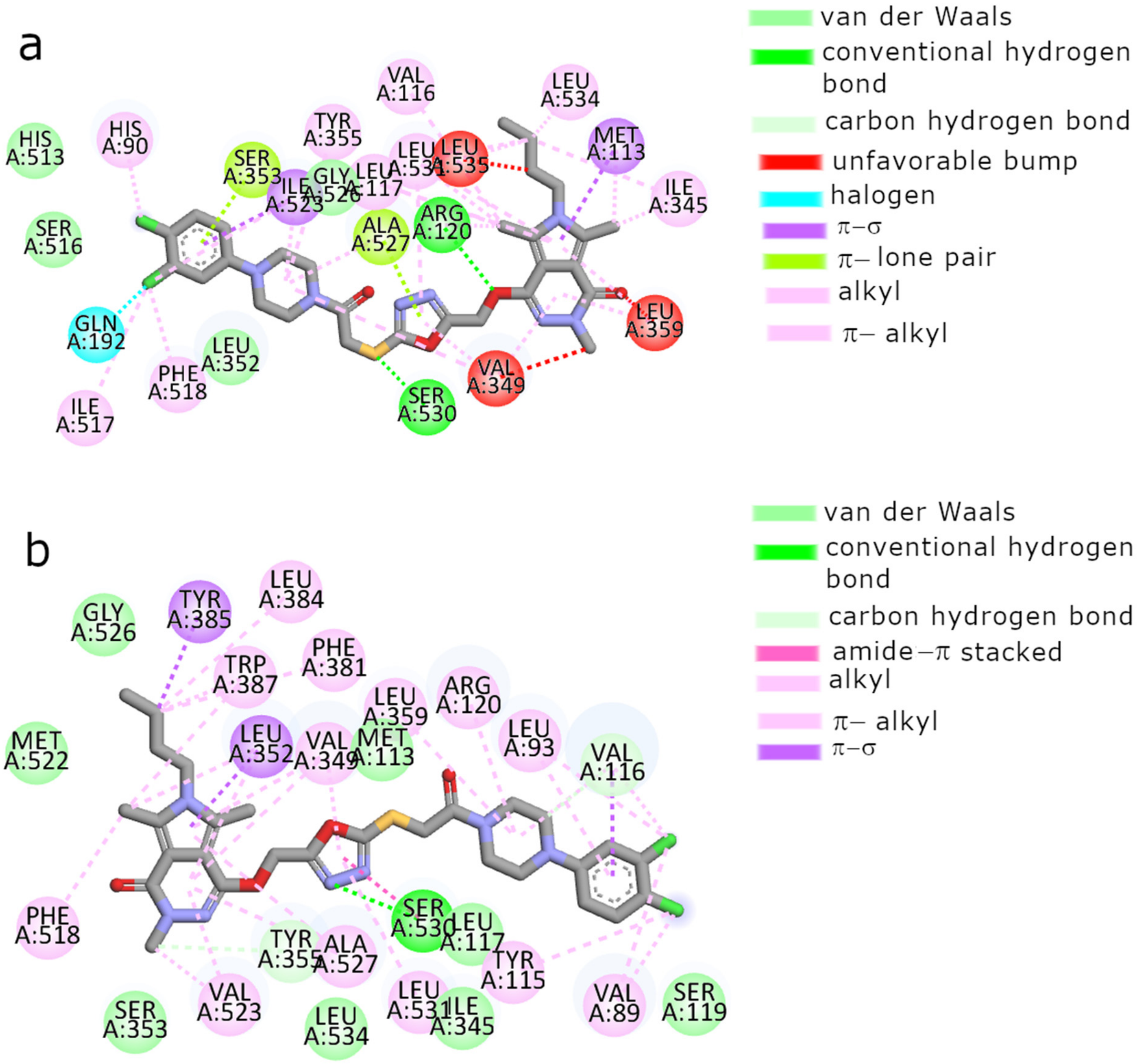

Table S6. Binding mode of investigated compounds with COX-1

Binding mode of compounds **a)** 2a, 2b, 3a, 3b, 4a, 4b; **b)** 5a, 5b, 6a, 6b, 7a, 7b; to the COX-1 obtained from molecular docking (by colour are marked amino acid residues responsible for meloxicam binding)

a

Compounds 2a 2b 3a 3b 4a 4b

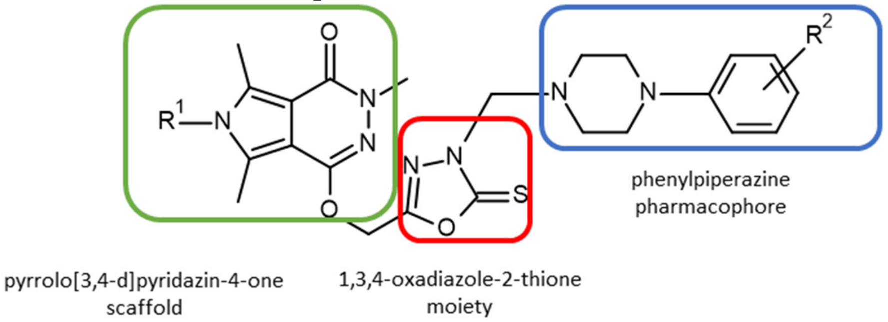

2a<br>R^1 ($-C_6H_6$) R^2 ($-CH_3$, $-CH_3$)	Leu117 Ile345 Val349 Leu352 Leu359 Met522 Gly526 Ala527 Ser530 Ile531 Leu534 Leu535	Leu93 Met113 Tyr355 Leu357 Leu359	Arg83 Pro86 Ile89 Val116 Val119 Arg120 Leu123 Glu524
2b<br>R^1 ($-C_4H_9$) R^2 ($-CH_3$, $- CH_3$)	Arg83 Pro86 Ser87 Ile89 His90 Leu93 Val116 Val119 Arg120 His513 Glu520 Ile523 Glu524	Leu93 Arg120 Tyr355 Leu359 Leu531	Tyr348 Val349 Leu352 Leu384 Tyr385 Trp387 Met522 Gly526 Ala527 Ser530
3a<br>R^1 ($-C_6H_6$) R^2 ($-F$, $-F$)	Leu117 Val349 Leu352 Leu357 Leu359 Trp387 Phe518 Met522 Gly526 Ala527 Ser530 Leu531	Ile89 Leu93 Val116 Arg120 Tyr355 Leu359	Pro86 Ser87 Ile89 His90 His513 Glu520 Ile523 Glu524
3b<br>R^1 ($-C_4H_9$) R^2 ($-F$, $-F$)	Leu93 Met113 Val116 Leu117 Arg120 Ile345 Val349 Leu352 Leu359 Tyr355 Gly526 Ala527	Val344 Tyr348 Leu352 Trp387 Ser530 Leu531 Ser353 Leu534	Phe205 Phe209 Gly227 Val228 Ile337 Asn375 Phe381 Tyr385 Ser530 Gly533
4a<br>R^1 ($-C_6H_6$) R^2 ($-Cl$, $-Cl$)	Leu93 Met113 Val116 Leu117 Arg120 Ile345 Val349 Leu357 Leu359 Ala527 Leu531 Leu534	Phe205 Val344 Tyr348 Leu352 Ser353 Leu354 Tyr355 Tyr385 Trp387 Gly526 Ser530	Phe205 Phe209 Val228 Asn375 Phe381 Tyr385 Gly533 Leu534
4b<br>R^1 ($-C_4H_9$) R^2 ($-Cl$, $-Cl$)	Val349 Leu352 Ser353 Phe381 Leu384 Tyr385 Trp387 Phe518 Met522 Ile523 Gly526 Ala527 Ser530 Leu351	Met113 Leu117 Val349 Leu531 Leu534 Leu535	Leu93 Met113 Val116 Arg120 Ile345 Tyr355 Leu357 Leu359
Meloxicam	Met113 Val116 Leu117 Arg120 Ile345 Val349 Leu352 Ser353 Tyr355 Leu359 Tyr385 Trp387 Phe518 Met522 Ile523 Gly526 Ala527 Ser530 Leu531 Leu534 Leu535		

b

Compounds 5a 5b 6a 6b 7a 7b

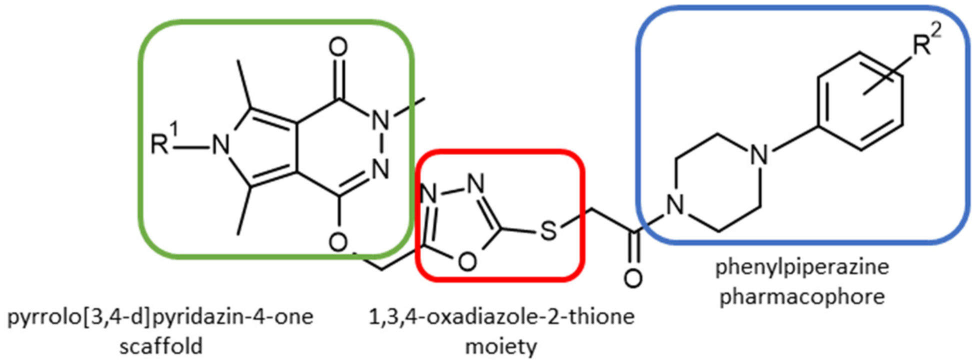

5a<br>R^1 (-C_6H_6) R^2 (-CH_3, -CH_3)	Met113 Leu117 Ile345 Val349 Leu352 Ile523 Gly526 Ala527 Ser530 Leu531 Leu534 Leu535	Arg120 Ser353 Leu359 Ala527	Arg83 Pro86 Ile89 Leu93 Met113 Val116 Val119 Arg120 Tyr355 Leu357 Glu524
5b<br>R^1 (-C_4H_9) R^2 (-CH_3, - CH_3)	Val349 Leu352 Ser353 Leu384 Phe381 Tyr385 Trp387 Phe518 Met522 Ile523 Gly526 Ser530	Val349 Tyr355 Leu359 Ala527 Leu531	Pro86 Ile89 His90 Leu93 Val116 leu117 Arg120 Leu357 Glu524
6a<br>R^1 (-C_6H_6) R^2 (-F, -F)	Ile345 Val344 Val349 Leu352 Ile523 Ala527 Ser530 Leu531 Leu534	Leu93 Met113 Val116 Leu117 Arg120 Tyr355 Leu357 Gly526 Leu535	Arg83 Ile89 Leu123 Gly471 Glu524 Pro528
6b<br>R^1 (-C_4H_9) R^2 (-F, -F)	Met113 Phe205 Val344 Val349 Leu352 Ser353 Tyr385 Trp378 Phe518 Ile523 Ala527 Ser530 Leu531 Ser535	Arg120 Ile345 Tyr355 Val349 Leu534 Leu359	Ile89 Leu93 Trp100 Leu112 Val116 Tyr355 Leu357
7a<br>R^1 (-C_6H_6) R^2 (-Cl, -Cl)	Ile345 Val349 Leu359 Met522 Ile523 Gly526 Ala527 Ser530 Leu531 Leu534	Met113 Val116 Ser353 Tyr355 Ala527	Pro86 Ile89 His90 Leu112 Val116 Leu117 Arg120 Leu357 Glu524 Leu535
7b<br>R^1 (-C_4H_9) R^2 (-Cl, -Cl)	Met113 Val116 Leu117 Arg120 Ile345 Val349 Leu359 Leu531 Leu534 Leu535	Leu117 Arg120 Val349 Tyr355 Ala527 Ser530	His90 Gln192 Leu352 Ser353 His513 Ser516 Ile517 Phe518 Ile523
Meloxicam	Met113 Val116 Leu117 Arg120 Ile345 Val349 Leu352 Ser353 Tyr355 Leu359 Tyr385 Trp387 Phe518 Met522 Ile523 Gly526 Ala527 Ser530 Leu531 Leu534 Leu535		

Table S7. Binding mode of investigated compounds with COX-2

Binding mode of compounds **a)** 2a, 2b, 3a, 3b, 4a, 4b; **b)** 5a, 5b, 6a, 6b, 7a, 7b; to the COX-2 obtained from molecular docking (by colour are marked amino acid residues responsible for meloxicam binding)

a

Compounds 2a 2b 3a 3b 4a 4b

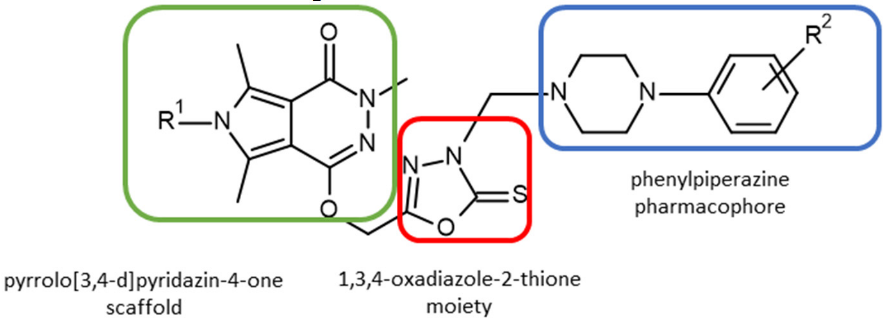

2a R^1 (-C_6H_6) R^2 (-CH_3, -CH_3)	Val349 Leu352 Ser353 Leu384 Trp387 Met522 Val523 Ala527 Ser530 Ser535	Ile345 Leu531 Leu534 Met535	Leu93 Met113 Val116 Leu117 Arg120 Tyr355 Phe357 Leu359 Phe381 Gly527
2b R^1 (-C_4H_9) R^2 (-CH_3, - CH_3)	His90 Tyr348 Val349 Leu352 Ser353 Phe381 Tyr385 Trp387 Met522 Val523 Gly526 Ala527 Ser530 Leu531	Leu93 Val116 Arg120 Tyr355	Met113 Leu117 Ile345 Leu359 Ser530 Leu531
3a R^1 (-C_6H_6) R^2 (-F, -F)	Leu93 Met113 Val116 Leu117 Arg120 Tyr355 Ala527 Leu531 Leu534 Met535 Leu359	Ser353 Phe518 Val523 Gly526 Ser530	Phe205 Thr206 Phe209 Val344 Tyr348 Val349 Leu352 Phe381 Tyr385 Trp387 Met522 Ser530
3b R^1 (-C_4H_9) R^2 (-F, -F)	His90 Val349 Ser352 Ser353 Phe381 Leu384 Tyr385 Trp387 Phe518 Met522 Val523 Gly526 Ala527 Met535	Leu93 Val116 Arg120 Tyr355	Met113 Val116 Leu117 Arg120 Ile349 Leu359 Ser530 Leu531 Leu534
4a R^1 (-C_6H_6) R^2 (-Cl, -Cl)	Leu93 Met113 Val116 Leu117 Arg120 Ile345 Tyr355 Leu359 Ala527 Leu531 Leu534 Met535	Val349 Leu352 Gly526 Ser530 Leu531	Phe205 Phe209 Gly227 Val228 Val344 Tyr348 Asn375 Ile377 Phe381 Tyr385 Trp387 Phe529 Ser530 Gly533
4b R^1 (-C_4H_9) R^2 (-Cl, -Cl)	Tyr348 Val349 Leu352 Tyr385 Trp387 Phe381 Ile517 Phe518 Met522 Val532 Gly526 Ala527 Ser530 Ser353	Val344 Val349 Ser530 Leu531 Ser353 Leu534	Met113 Leu117 Arg120 Ile345 Tyr355 Leu359 Leu531 Met535
Meloxicam	Met113 Val116 Leu117 Arg120 Ile345 Val349 Leu352 Ser353 Tyr355 Leu359 Tyr385 Trp387 Phe518 Met522 Val523 Ala527 Ser530 Leu531 Leu534 Met535		

b

Compounds 5a 5b 6a 6b 7a 7b

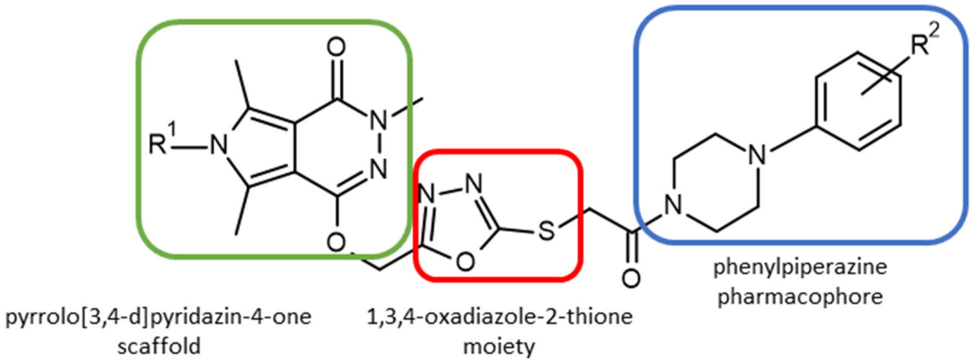

5a<br>R^1 (-C_6H_6) R^2 (-CH_3, -CH_3)	His90 Leu352 Ser353 Phe381 Leu384 Tyr385 Trp387 Phe518 Met522 Val523 Gly526 Ala527 Ser530	Arg120 Tyr355 Ala527 Ser530	Met113 Val116 Leu117 Arg120 Val344 Ile345 Leu534 Met535
5b<br>R^1 (-C_4H_9) R^2 (-CH_3, - CH_3)	Tyr348 Val349 Leu352 Phe381 Tyr385 Trp387 Val523 Phe518 Met522 Gly526 Ala527 Ser530 Ser535	Tyr355 Ala527 Ser530	Leu93 Met113 Val116 Leu117 Arg120 Ile345 Phe357 Leu359 Leu531
6a<br>R^1 (-C_6H_6) R^2 (-F, -F)	Leu352 Ser353 Phe381 Tyr385 Trp387 Met522 Val523 Gly526 Phe518 Ala527	His90 Val349 Ser530 Leu531 Leu534	Leu93 Met113 Val116 Leu117 Arg120 Ile345 Tyr355 Leu359 Met535
6b<br>R^1 (-C_4H_9) R^2 (-F, -F)	Tyr348 Val349 Leu352 Phe381 Leu384 Tyr385 Trp387 Phe518 Met522 Val523 Gly526 Ala527 Leu534	Arg120 Val349 Tyr355 Ser530 Leu531 Ser353	Val89 Leu93 Met113 Val116 Leu117 Ser119 Arg120 Phe357 Leu359
7a<br>R^1 (-C_6H_6) R^2 (-Cl, -Cl)	Phe381 Leu384 Tyr385 Trp387 Leu352 Phe518 Met522 Val523 Gly526 Ser530	His90 Val349 Tyr355 Ala527 Ser530 Ser353 Leu534	Val89 Leu93 Met113 Val116 Leu117 Arg120 Ile345 Phe357 Leu359 Leu531
7b<br>R^1 (-C_4H_9) R^2 (-Cl, -Cl)	Val349 Leu352 Ser353 Tyr355 Phe381 Leu384 Tyr385 Trp387 Peh518 Met522 Val523 Gly526 Leu534	Met113 Ile345 Leu349 Leu359 Ala527 Ser530 Leu531	Val89 Leu93 Tyr115 Val116 Leu117 Ser119 Arg120 Ile345
Meloxicam	Met113 Val116 Leu117 Arg120 Ile345 Val349 Leu352 Ser353 Tyr355 Leu359 Tyr385 Trp387 Phe518 Met522 Val523 Gly526 Ala527 Ser530 Leu531 Leu534 Leu535		
